# Two-component assembly of recognition-encoded oligomers that form stable H-bonded duplexes†
†This article is dedicated to Maria Cignoni.
‡
‡Electronic supplementary information (ESI) available: Materials and methods, synthetic procedures, full characterization of all compounds and NMR titration data. See DOI: 10.1039/c9sc04250d


**DOI:** 10.1039/c9sc04250d

**Published:** 2019-11-29

**Authors:** Luca Gabrielli, Diego Núñez-Villanueva, Christopher A. Hunter

**Affiliations:** a Department of Chemistry , University of Cambridge , Lensfield Road , Cambridge CB2 1EW , UK . Email: herchelsmith.orgchem@ch.cam.ac.uk

## Abstract

A new family of recognition-encoded oligomers that form stable duplexes in chloroform have been prepared. Monomer building blocks composed of dialdehydes functionalised with either a trifluoromethylphenol or phosphine oxide H-bond recognition unit were prepared. The dialdehydes were coupled with diamines by imine formation and then reduction to give homo-oligomers between one and three recognition units in length. Duplex formation was characterised by ^19^F and ^1^H NMR titration experiments in toluene and in chloroform. For duplexes formed between length complementary H-bond donor and acceptor homo-oligomers, an order of magnitude increase in stability was observed for every base-pair added to the duplex in chloroform. The effective molarity for the intramolecular H-bonds responsible for zipping up the duplex is 30 mM, which results in the fully assembled duplex in all cases. The uniform increase in duplex stability with oligomer length suggests that the backbone structure and geometry is likely to be compatible with the formation of extended duplexes in longer oligomers.

## Abstract 2

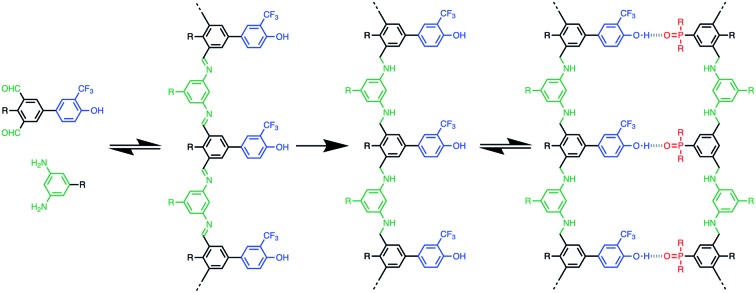
Imine chemistry was used to assemble oligomers displaying phenol and phosphine oxide side chains that selectively base-pair to give duplexes, which are stable in chloroform solution.

## Introduction

Duplex formation between complementary oligomers is one of the most important processes in biology, and has found a wide range of applications in nanotechnology. The nucleic acid duplex provides a robust architecture for encoding molecular information and for replication, transcription and translation of this information through template-directed synthesis.^1^,^2^ These properties are currently unique to nucleic acids, so it is not surprising that the first approaches to synthetic sequence-controlled macromolecules use biopolymers. A range of nucleic acid analogues that form duplexes have been prepared modifying the phosphate diester,^3^ the bases,^4^ and the sugar.^5^ Recently, DNA-templated polymerization and *in vitro* selection was used to evolve oligomers with chemically diverse side chains.^6^

**Fig. 1:**
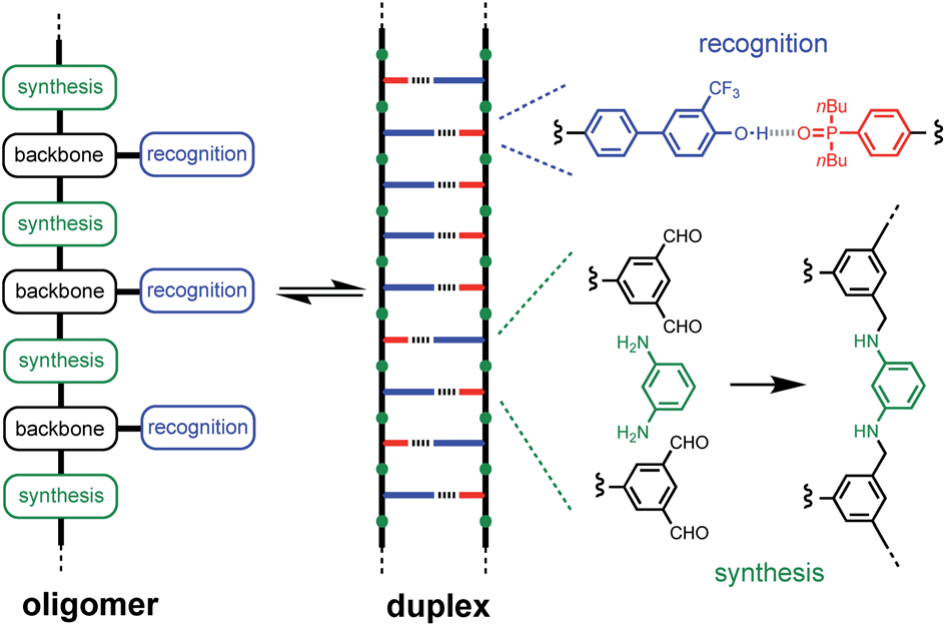
A blueprint for duplex forming molecules. There are three key design elements: the coupling chemistry used for the synthesis of oligomers (green), the recognition module which controls intermolecular binding (blue/red) and the backbone module which links these components together (black). The long-short trifluorophenol–phosphine oxide base-pairing system is shown, along with a backbone that can be assembled using reductive coupling of dialdehydes and diamines.

Synthetic oligomers that bear no relation to nucleic acids have also been shown to form duplexes *via* non-covalent interactions such as hydrogen bonding,^7^ aromatic interactions,^8^ salt bridges,^9^ and metal–ligand coordination.^10^ Sequence-selective duplex formation has been demonstrated for short sequences, showing that it is possible to read and write sequence information encoded into synthetic oligomers.^11^ We have been exploring the blueprint shown in Fig. 1 as a template for the design of duplex forming molecules. The recognition units that form the base-pairs in the duplex are displayed as side chains on the oligomers, so that the three key elements of the blueprint (the synthesis, backbone and recognition modules) can be independently optimised. A two-letter alphabet is used to encode information in an oligomer as a sequence of H-bond donor and acceptor sites. Provided the backbone does not contain any polar functional groups that could compete with H-bonding interactions between the recognition units, reliable duplex assembly can be achieved in non-polar solvents like toluene. We recently showed that the trifluoromethylphenol–phosphine oxide base-pair illustrated in Fig. 1 is sufficiently stable to allow duplex formation in more polar solvents like chloroform.^12^

**Fig. 2:**
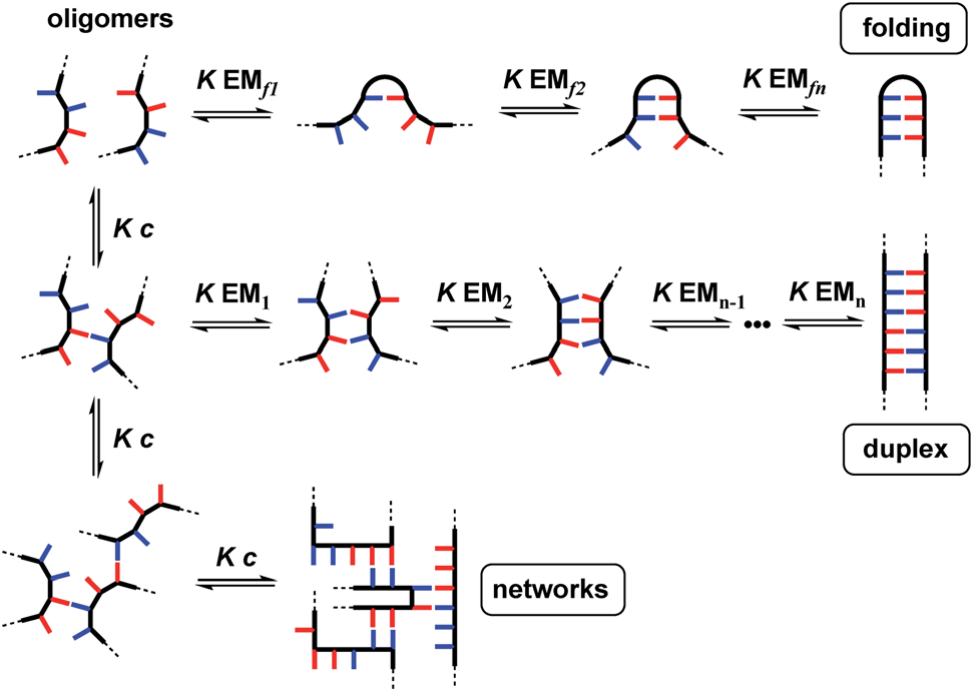
Self-assembly channels for recognition-encoded oligomers. The first base-pairing interaction can take place in an intramolecular fashion leading to the folding channel or in an intermolecular fashion. The second base-pairing interaction can take place in an intramolecular fashion to initiate duplex formation or in an intermolecular fashion leading to the networks channel. The outcome depends on the concentration, *c*, the association constant for the intermolecular base-pairing interaction, *K*, and the effective molarities for folding, EM_f1_, duplex initiation, EM_1_ and duplex propagation, EM_*n*_.

The properties of the backbone are also important, because there are different self-assembly channels that compete with duplex formation (Fig. 2). The parameters that determine the outcome of the self-assembly of recognition-encoded oligomers are the effective molarities for intramolecular folding (EM_f_), the effective molarities for duplex formation (EM_*n*_), the strength of the H-bond interaction (*K*) and the operating concentration (*c*). In the absence of geometric constraints, the values of EM are usually in the 10–100 mM range, so operating at millimolar concentrations avoids the intermolecular networks channel. If the backbone is too flexible, the folding pathway dominates due to intramolecular H-bonding interactions between donors and acceptors that are adjacent in sequence. The use of the long-short phenol–phosphine oxide base-pair illustrated in Fig. 1 reduces the probability of these 1,2-folding interactions and promotes duplex formation.^12^ It is also possible to decrease EM_f1_ by using a rigid backbone,^13^ but if the backbone is too rigid, duplex assembly fails.^14^ Duplex initiation, formation of the second base-pair, is governed by EM_1_ (see Fig. 2) and is favourable in all of the systems that we have studied to date. However, duplex propagation, formation of subsequent base-pairs (EM_2_ … EM_*n*_), is highly dependent on the conformational properties of the backbone. Thus, a semi-flexible backbone is required to ensure that the molecule can adjust to a conformation compatible with duplex formation.

Here we describe a new family of oligomers that form stable duplexes in chloroform solution. The long-short trifluorophenol–phosphine oxide base-pair illustrated in Fig. 1 is used for the recognition module, because we have previously shown that this system reduces folding and increases duplex stability.^12^ The backbone is assembled from two different components, a dialdehyde that carries the recognition units and a diamine linker (Fig. 1). Imine formation or reductive amination can be used for oligomer synthesis, and the two-component approach provides the opportunity to introduce variation in the properties of the backbone by changing the diamine without the need to synthesise new monomer building blocks. The reversibility of imine bond formation also opens up possibilities in dynamic covalent chemistry and template synthesis.^15^ Reduction of the imines will result in a backbone that contains secondary amines, which are both H-bond donors and acceptors. However, the use of aniline nitrogens ensures that there will be no competition with the base-pairing interactions between the recognition units: the H-bond acceptor parameter (*β*) for aniline is 5 compared with 10 for phosphine oxide, and the H-bond donor parameter (*α*) for aniline is 2 compared with 4 for trifluorophenol.^16^

## Results and discussion

### Synthesis

**Scheme 1:**
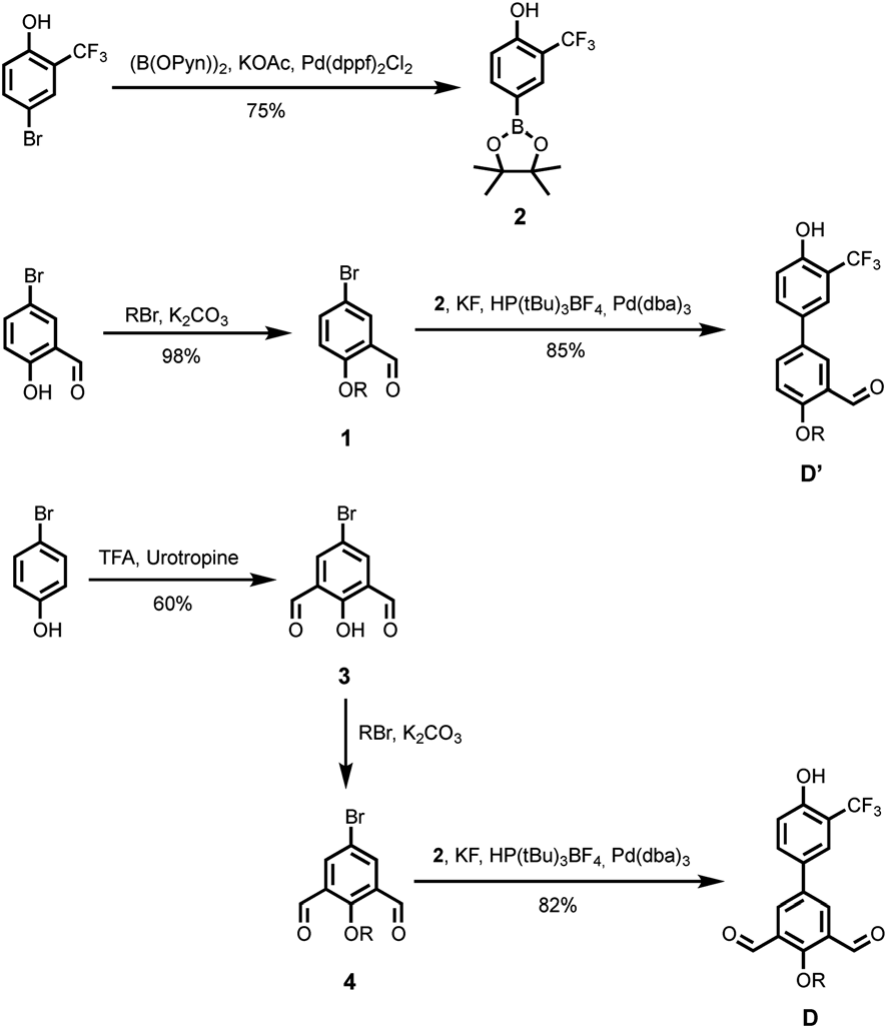
Synthesis of phenol building blocks **D′** and **D** (R = 2-ethylhexyl).

The synthetic route to the mono-aldehyde and di-aldehyde phenol building blocks is shown in Scheme 1. 5-Bromosalicylaldehyde was alkylated with racemic 2-ethylhexyl bromide to give **1**. 4-Bromo-2-(trifluoromethyl)phenol was converted to the boronic ester **2** under microwave irradiation with palladium catalysis. Suzuki–Miyaura coupling of **1** with **2** under microwave irradiation using palladium catalysis gave the mono-aldehyde phenol **D′**. 5-Bromo-2-hydroxyisophthalaldehyde **3** was synthesised *via* a Duff reaction, by reacting *p*-bromophenol with an excess of hexamethylenetetramine.^17^ Subsequent alkylation with racemic 2-ethylhexyl bromide gave **4**, which was coupled with **2** under Suzuki–Miyaura conditions to give the di-aldehyde phenol **D**.

**Scheme 2:**
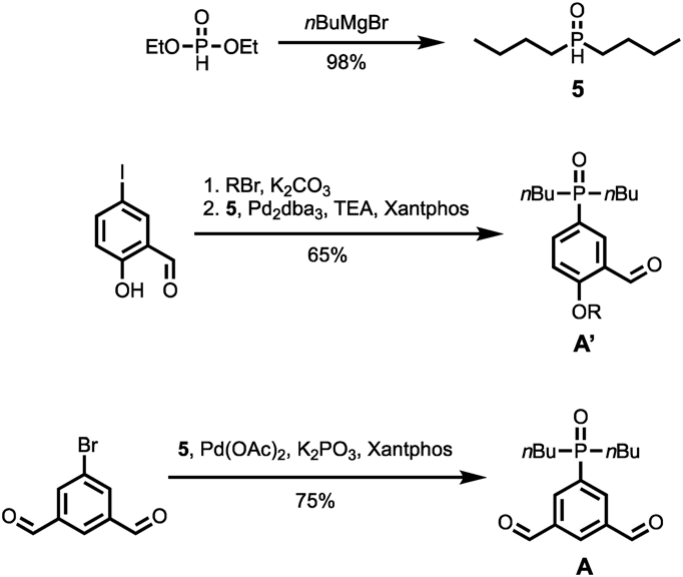
Synthesis of phosphine oxide building blocks **A′** and **A** (R = 2-ethylhexyl).

The synthetic route to the mono-aldehyde and di-aldehyde phosphine oxide building blocks is shown in Scheme 2. Treatment of diethyl phosphite with butylmagnesium chloride gave **5**. The mono-aldehyde phosphine oxide **A′** was synthesised from 5-iodosalicylaldehyde by alkylating with racemic 2-ethylhexyl bromide and then coupling with **5** using palladium and XantPhos under microwave irradiation. Similarly, the di-aldehyde phosphine oxide **A** was synthesised from 5-bromo-isophthalaldehyde by coupling with **5** using palladium and XantPhos under microwave irradiation.

**Scheme 3:**
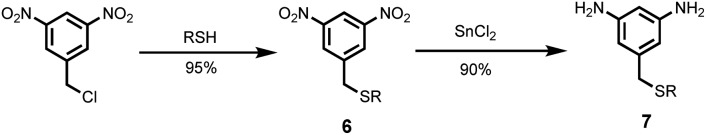
Synthesis of diamine linker **7** (R = 2-ethylhexyl).

Two different 1,3-phenylenediamines were used as linkers for the synthesis of oligomers: 5-(trifluoromethyl)-1,3-phenylenediamine, which is commercially available; and compound **7**, which was prepared by treatment of 1-(chloromethyl)-3,5-dinitrobenzene with racemic 2-ethylhexyl thiol to give **6**, and subsequent reduction with tin(ii) chloride to give **7** (Scheme 3).

**Scheme 4:**
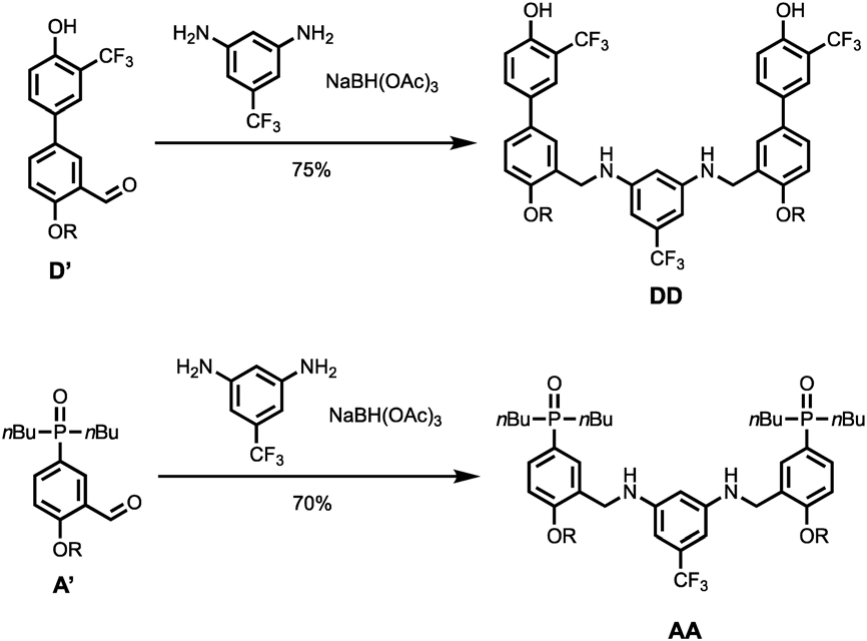
Synthesis of **DD** and **AA** (R = 2-ethylhexyl).

Scheme 4 shows the synthesis of 2-mers **DD** and **AA**. Reductive amination of **D′** or **A′** with 5-(trifluoromethyl)-1,3-phenylenediamine and NaBH(OAc)_3_ gave **DD** and **AA** respectively in good yield.

**Scheme 5:**
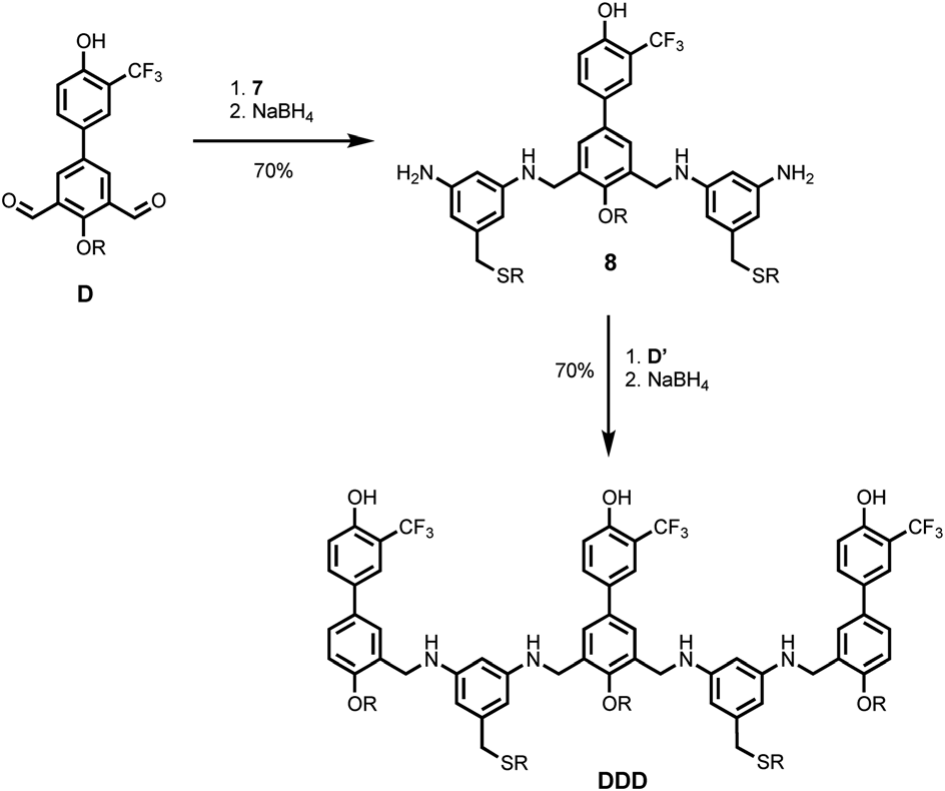
Synthesis of **DDD** (R = 2-ethylhexyl).

**Scheme 6:**
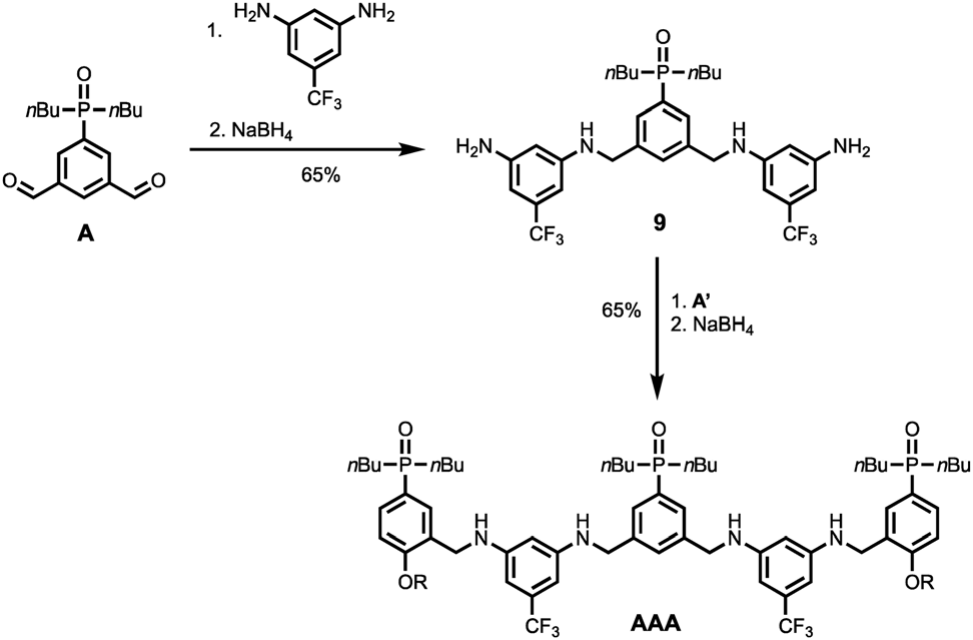
Synthesis of **AAA** (R = 2-ethylhexyl).

Schemes 5 and 6 show the synthesis of 3-mers **DDD** and **AAA**. The phosphine oxide building blocks carry additional solubilising groups on the phosphorus, so 5-(trifluoromethyl)-1,3-phenylenediamine was used as the linker for synthesis of the acceptor 3-mer. In all cases, racemic 2-ethylhexyl solubilising groups were used, because the diastereoisomeric mixture improves solubility. However, the donor 3-mer required additional solubilising groups, so **7**, the diamine equipped with a solubilising group, was used as the linker for this oligomer. This group is too remote from the recognition groups to affect duplex formation. Treatment of the di-aldehyde building block (**D** or **A**) with nine equivalents of the corresponding 1,3-phenylenediamine gave the di-imine, which was reduced with NaBH_4_ to give **8** (Scheme 5) or **9** (Scheme 6). Treatment of **8** with mono-aldehyde **D′** followed by reduction with NaBH_4_ gave 3-mer **DDD**, and treatment of **9** with mono-aldehyde **A′** followed by reduction with NaBH_4_ gave 3-mer **AAA**.

### NMR binding studies

Complexation of length-complementary oligomers was studied using ^1^H and ^19^F NMR titration experiments. The association constant *K* for formation of the **A·D** complex, which makes a single intermolecular hydrogen bond, was measured by titrating **A** into **D** in toluene. Addition of **A** caused an upfield change in the ^19^F NMR chemical shift of the signal due to the **D** CF_3_ group and a downfield change in the ^1^H-NMR chemical shift of the signal due to the **D** OH group. The titration data fit well to a 1 : 1 binding isotherm, giving an association constant of 3 × 10^3^ M^–1^ (Table 1). Although the aldehyde substituents on **A** and **D** differ from the reductive amination products, these groups have no effect on the H-bonding properties of the phosphine oxide and phenol recognition groups, and the association constant measured in toluene is identical to the value measured previously for the corresponding monomers with dialkylamino substituents.

**Table 1:** Association constants (*K*), effective molarities (EM), limiting NMR chemical shifts (*δ*_free_ and *δ*_bound_) and complexation-induced changes in chemical shift (Δ*δ*) for the formation of duplexes at 298 K^*a*^

Solvent	Complex	log *K* (M^–1^)	EM (mM)	^19^F-NMR^*b*^ (ppm)			^1^H-NMR^*b*^ (ppm)		
				*δ*_free_	*δ*_bound_	Δ*δ*	*δ*_free_	*δ*_bound_	Δ*δ*
CDCl_3_	**D·A**	2.3 ± 0.1	—	–61.1	–62.8	–1.7	5.7	11.3	5.6
	**DD·AA**	3.5 ± 0.1	35	–61.0	–62.3	–1.2	5.5	10.3	4.8
	**DDD·AAA**	4.4 ± 0.1	35	–61.0	–62.2	–1.2			
				–61.1	–62.2	–1.0			
Toluene	**D·A**	3.5 ± 0.1	—	–61.8	–62.3	–0.5	4.9	11.6	6.6
	**DD·AA**	5.7 ± 0.1	31	–61.5	–61.8	–0.3			

^*a*^Each titration was repeated twice and the average value of the association constant from the ^19^F NMR titrations is reported with errors at the 95% confidence limit.

^*b*^Data for the signals due to the OH and CF_3_ groups on the phenol recognition units.

Addition of **AA** into **DD** caused an upfield shift of the ^19^F NMR signal due to the CF_3_ group on the phenol recognition unit of **DD** and a small downfield shift of the signal due to the CF_3_ group on the diamine linker unit. The data fit well to a 1 : 1 binding isotherm, giving an association constant of 5 × 10^5^ M^–1^. The association constant measured for **AA·DD** is two orders of magnitude higher than the value measured for **A·D**, which indicates that **AA·DD** forms a fully-assembled duplex with cooperative formation of two H-bonds. The association constants were used to determine the effective molarity (EM) for formation of the second intramolecular H-bond in the **AA·DD** duplex (eqn (1)).^18^

1 *K*_N_ = 2*K*_1_^*N*^EM^*N*–1^

where *K*_N_ is the association constant for duplex formation between two oligomers with *N* interaction sites, *K*_1_ is the association constant for formation a single intermolecular H-bond in **A·D**, and two is the statistical factor 2 that takes into account the parallel–antiparallel degeneracy of the duplex.

For the **AA·DD** duplex, EM is 31 mM, which is consistent with the values of EM that we have measured for duplex forming oligomers with different backbones and base-pairing systems (10–100 mM).12b,^18^ The chelate cooperativity associated with duplex formation is expressed as the product *K*_1_ EM, which is equal to 80, implying that the doubly H-bonded closed duplex is almost exclusively populated (98%).12*a* The complex formed by the two 3-mers **AAA** and **DDD** was too stable in toluene for measurement of the association constant by NMR titration, so all of the binding studies were repeated in chloroform.

**Fig. 3:**
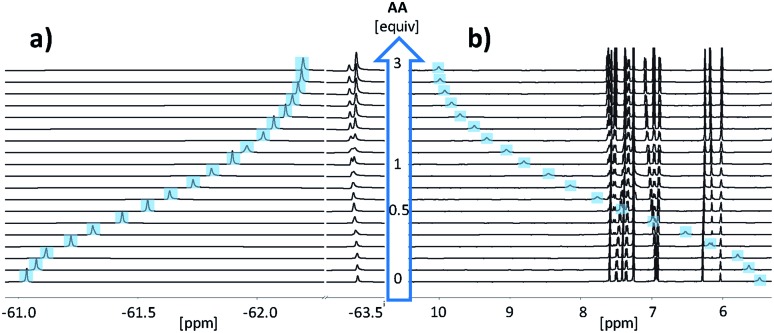
Partial NMR spectra for titration of **AA** into **DD** (2.3 mM) in *d*-chloroform at 298 K. (a) 470 MHz ^19^F NMR spectra with the signal due to the CF_3_ group on the phenol recognition unit of **DD** highlighted in blue. (b) 500 MHz ^1^H-NMR spectra with the signal due to the OH group on the phenol recognition unit of **DD** highlighted in blue.

Fig. 3 shows the NMR spectra for titration of **AA** to **DD** in chloroform. Addition of **AA** caused a large upfield change in the ^19^F NMR chemical shift of the signal due to the **DD** CF_3_ group and a large downfield change in the ^1^H-NMR chemical shift of the signal due to the **DD** OH group. The titration data fit well to a 1 : 1 binding isotherm for all three length complementary complexes, and the results are collected in Table 1.

The association constants are significantly lower than the values measured in toluene, but the association constant for **AA·DD** is still an order of magnitude higher than the value measured for **A·D**, indicating cooperative assembly of the **AA·DD** duplex. Similarly, the association constant for **AAA·DDD** is an order of magnitude higher than the value measured for **AA·DD**, indicating that all three H-bonds are formed in the **AAA·DDD** duplex. For all three complexes, the values of Δ*δ* for the OH and CF_3_ groups on the trifluorophenol recognition units are similar, which confirms that the donor recognition units are fully H-bonded in all of the complexes (Table 1). Using eqn (1) to determine EM gives a value of 35 mM for both duplexes in chloroform, which is similar to the value in toluene. However, the reduction in the association constant for H-bond formation in chloroform means that the chelate cooperativity associated with duplex formation (*K*_1_ EM) is reduced by an order of magnitude to 7.

The fact that the EM for formation of the **AAA·DDD** duplex is the same as the value for formation of the **AA·DD** duplex is an important result, which suggests that it should be possible to assemble stable duplexes using longer oligomers. As we have shown previously, duplex initiation is relatively insensitive to the conformational properties of the backbone, so the value of EM_1_ in Fig. 1 is usually high enough to give cooperative formation of two H-bonds. However, the subsequent values of EM_2_, EM_3_*etc* leading to duplex propagation can be much lower in systems with a backbone that is incompatible with formation of an extended duplex.^14^,^18^ The results in Table 1 show similar values of EM for duplex initiation and propagation, which indicates that this backbone will support formation of longer duplexes.^14^,^18^,^19^

**Fig. 4:**
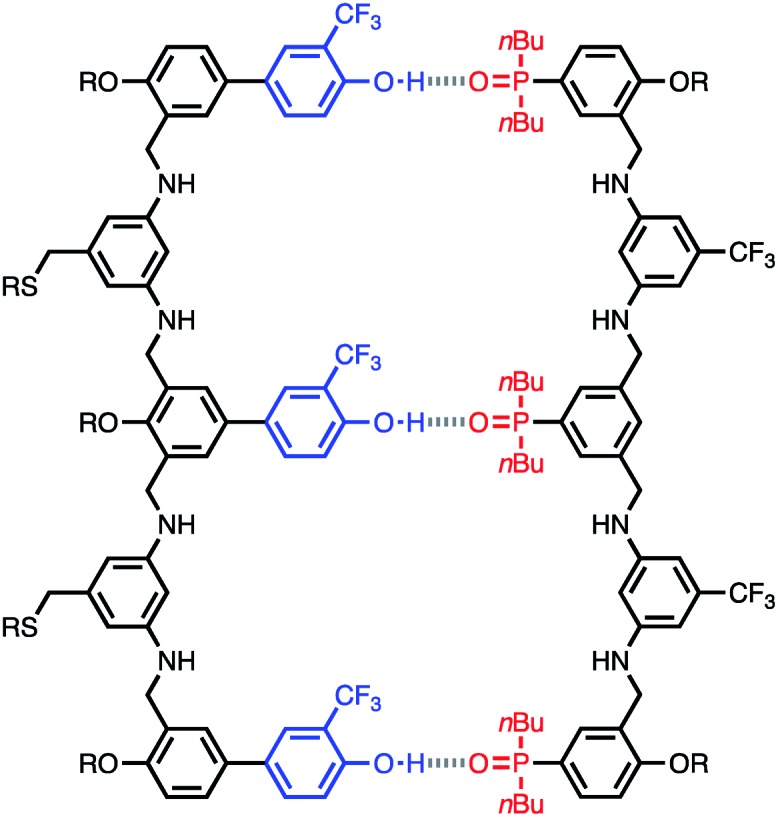
Structure of the **AAA·DDD** duplex.

The structure of the **AAA·DDD** duplex is illustrated in Fig. 4. Using the values of *K*_1_ and EM measured in toluene in eqn (1), the association constant for assembly of this complex in toluene can be estimated as 9 × 10^7^ M^–1^, which is too high to measure using NMR titration, consistent with the experimental observation.

### Thermal denaturation experiments

**Fig. 5:**
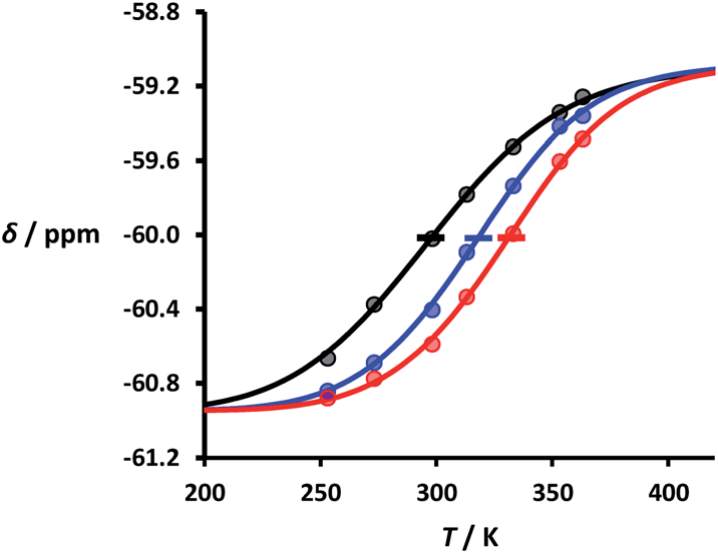
Experimental ^19^F NMR chemical shift plotted as a function of temperature for 1 : 1 mixtures (2 mM) of **A·D** (black), **AA·DD** (blue), and **AAA·DDD** (red) in 1,1,2,2-tetrachloroethane. The lines are the best fit to eqn (2) (total rmsd < 0.01 ppm), and the transition melting temperatures are indicated with a bar. The optimised values of *T*_m,*N*_, and 
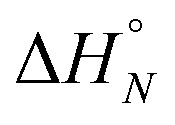
 are 298 K and –37 kJ mol^–1^ for **A·D**, 318 K and –51 kJ mol^–1^ for **AA·DD**, and 331 K and –55 kJ mol^–1^ for **AAA·DDD**.

Thermal denaturation experiments were carried out to extract thermodynamic parameters for duplex assembly. ^19^F NMR spectra of 1 : 1 solutions of length-complementary oligomers at 2 mM concentrations in 1,1,2,2-tetrachloroethane were recorded at different temperatures between 253 and 363 K. The changes in the chemical shifts of the ^19^F NMR signals due to the phenol CF_3_ groups are indicative of an increase in the population of H-bonded complexes at lower temperatures and disruption of the duplex at higher temperatures (Fig. 5). These melting data were fit to a two-state model, assuming that only duplex and denatured single strands are present, that the enthalpy and entropy changes for the formation of the *N*-mer duplex (
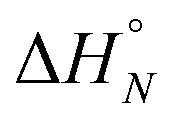
 and 
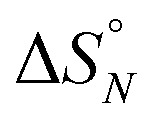
) are temperature independent, and that the change in heat capacity between free and bound states is zero (eqn (2)).^18^2
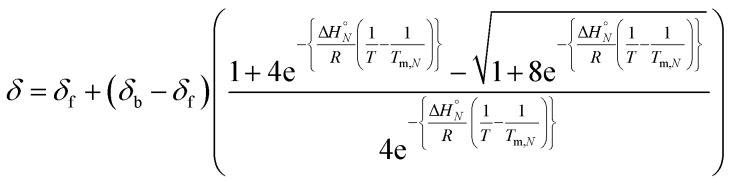
where *δ* is the observed chemical shift at temperature *T*, *δ*_f_ and *δ*_b_ are the chemical shifts of the single strand and duplex states respectively, and *T*_m,*N*_ is the transition melting temperature for the *N*-mer duplex.

Fig. 5 shows the best fit of eqn (2) to the experimental melting data. The values of the transition melting temperatures increase from 298 to 331 K as the length of the duplex increases, and the enthalpy change on duplex formation becomes progressively more favourable with increasing numbers of H-bonds. These observations are indicative of cooperative H-bonding interactions along the duplex.

## Conclusions

A new family of recognition-encoded oligomers that form stable duplexes in chloroform have been prepared. The modular synthesis uses dialdehydes functionalised with either a trifluorophenol or phosphine oxide recognition unit and diamines to obtain oligomers by imine formation and then reduction. A series of homo-oligomers were synthesised, and duplex formation was characterised by NMR titration experiments. When length complementary oligo-trifluorophenols and oligo-phosphine oxides were combined, an order of magnitude increase in stability was observed for every base-pair added to the duplex. The effective molarity for the intramolecular H-bonds responsible for zipping up the duplex is about 30 mM in toluene and in chloroform. The uniform increase in duplex stability with oligomer length suggests that the backbone structure and geometry is likely to be compatible with the formation of extended duplexes in longer oligomers. This two-component backbone is more versatile than previous designs, because it provides an opportunity for varying the diamine component without the need to resynthesise complex monomer building blocks. The properties of mixed sequence oligomers and template-directed synthesis using dynamic imine chemistry are currently under investigation.

## Conflicts of interest

There are no conflicts to declare.

## Supplementary Material

Supplementary information
