# Supplementary material for: Two-component assembly of recognition-encoded oligomers that form stable H-bonded duplexes
Source: Chem Sci. 2019 Nov 29;11(2):561–6. doi: 10.1039/c9sc04250d (PMC7069511; doi:10.1039/c9sc04250d)
Supplement: Supplementary file 1 [file SC-011-C9SC04250D-s001.pdf]

Electronic Supplementary Material (ESI) for Chemical Science.
This journal is © The Royal Society of Chemistry 2019

Supporting Information

Two-component assembly of recognition-encoded oligomers that form stable H-bonded duplexes

Luca Gabrielli, Diego Nűñez-Villanueva and Christopher A. Hunter*

Department of Chemistry, University of Cambridge, Lensfield Road, Cambridge CB2 1EW, UK

Table of Contents

1. Experimental procedures........S2
2. Synthesis and characterization of monomer **D'**........S3
3. Synthesis and characterization of monomer **D**........S17
4. Synthesis and characterization of monomer **A'**........S20
5. Synthesis and characterization of monomer **A**........S26
6. Synthesis and characterization of dimer **DD**........S30
7. Synthesis and characterization of dimer **AA**........S35
8. Synthesis and characterization of Aniline **7**........S40
9. Synthesis and characterization of trimer **DDD**........S45
10. Synthesis and characterization of trimer **AAA**........S54
11. NMR binding studies........S64
12. References........S74

1. Experimental Procedures

General: The reagents and materials used in the synthesis of the compounds described below were bought from commercial sources, without prior purification. UV irradiations were performed using an UVP lamp model UVGL-58 (1x365 nm tube, 6 watt) and an UVP lamp model UVL-28 (2x365 nm tubes, 8 watt). Thin layer chromatography was carried out using with silica gel 60F (Merck) on aluminium. Flash chromatography was carried out on an automated system (Combiflash Companion, Combiflash Rf+ or Combiflash Rf Lumen) using prepacked cartridges of silica (25µ or 50µ PuriFlash® Columns). All NMR spectroscopy was carried out on a Bruker AVI250, AVI400, DPX400, AVIII400 spectrometer using the residual solvent as the internal standard. All chemical shifts (δ) are quoted in ppm and coupling constants given in Hz. Splitting patterns are given as follows: s (singlet), d (doublet), t (triplet), q (quadruplet), m (multiplet). FT-IR spectra were measured on a PerkinElmer Spectrum 100 or One spectrometer equipped with an ATR cell. Melting points were measured in a Mettler Toledo MP50 Melting Point System. Optical activity was measured in an AA-10 or an Anton Paar (MCP 100) at 589 nm. ES+ was carried out on a Waters LCT-TOF spectrometer or a Waters Xevo G2-S bench top QTOF machine.

2. Synthesis of monomer D'

Monomer **D'** was prepared as reported in the following scheme:

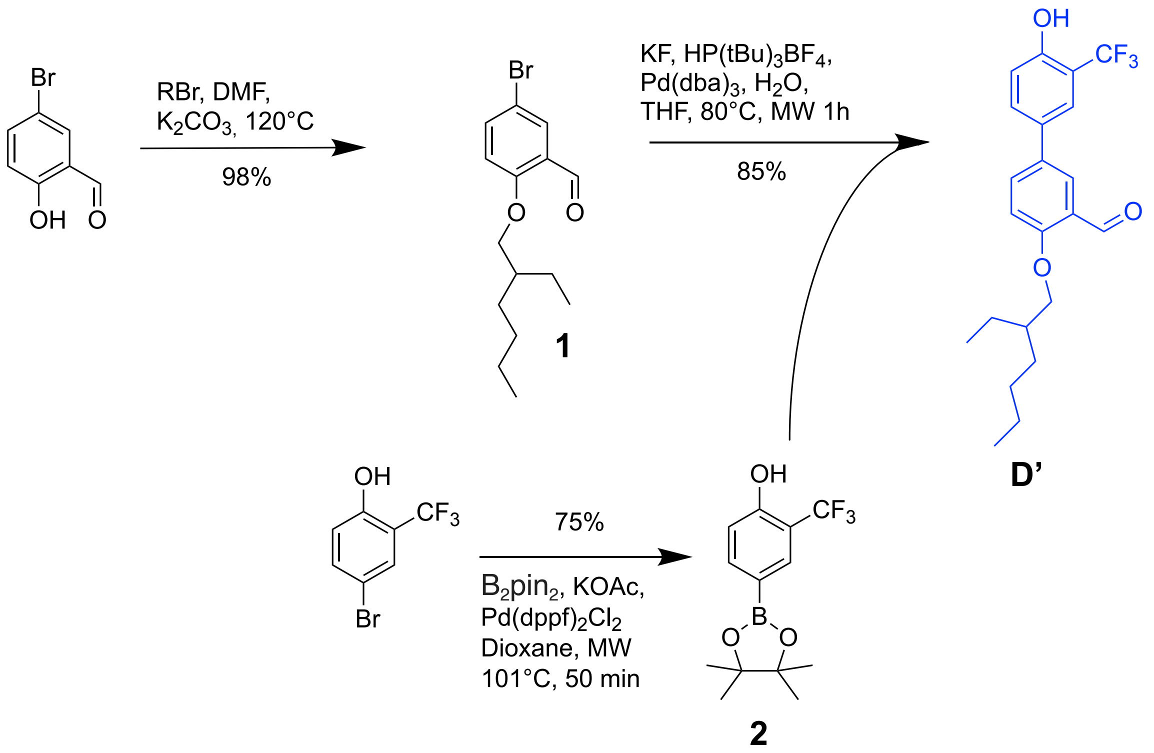

2.1 Synthesis of 1.

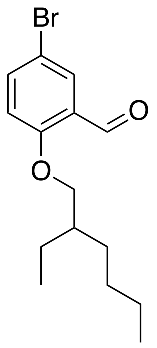

1

5-bromo-2-hydroxybenzaldehyde (1 g, 4.975 mmol, 1 equiv) and 3-(bromomethyl)heptane (3.54 mL, 19.899 mmol, 4 equiv) were dissolved in dry DMF (30 mL); K_2CO_3 (2.75 g, 19.899 mmol, 4 equiv) anhydrous was added and the mixture was stirred at 120°C for 5 hours. Then the reaction mixture was extracted 3 times with EtOAc and LiCl 5% solution. The organic layers were collected, dried over $MgSO_4$, concentrated under vacuum and purified by combiflash, giving 1.5 g of a colourless oil (98% yield).

^{1}H NMR (400 MHz, $CDCl_3$) δ 10.43 (s, 1H), 7.92 (d, *J* = 2.6 Hz, 1H), 7.61 (dd, *J* = 8.9, 2.7 Hz, 1H), 6.91 (d, *J* = 8.9 Hz, 1H), 3.97 (d, *J* = 6.5 Hz, 2H), 1.87 – 1.74 (m, 1H), 1.56 – 1.41 (m, 4H), 1.38 – 1.27 (m, 4H), 0.96 (t, *J* = 7.5 Hz, 3H), 0.94 – 0.88 (m, 3H).
^{13}C NMR (101 MHz, $CDCl_3$) δ 188.31, 160.60, 138.26, 130.73, 126.21, 114.53, 113.18, 71.26, 39.35, 30.55, 29.05, 23.94, 22.97, 14.04, 11.14.
HRMS (ES+): Calculated for $C_{15}H_{22}{}^{79}BrO_2$ 313.0798 a.m.u., found 313.0785 a.m.u.
FT-IR (ATR): $ν_{max}$ / cm^{-1} 2958, 2928, 2859, 1725, 1682, 1590, 1482, 1461, 1384, 1268, 1240, 1176, 1122.

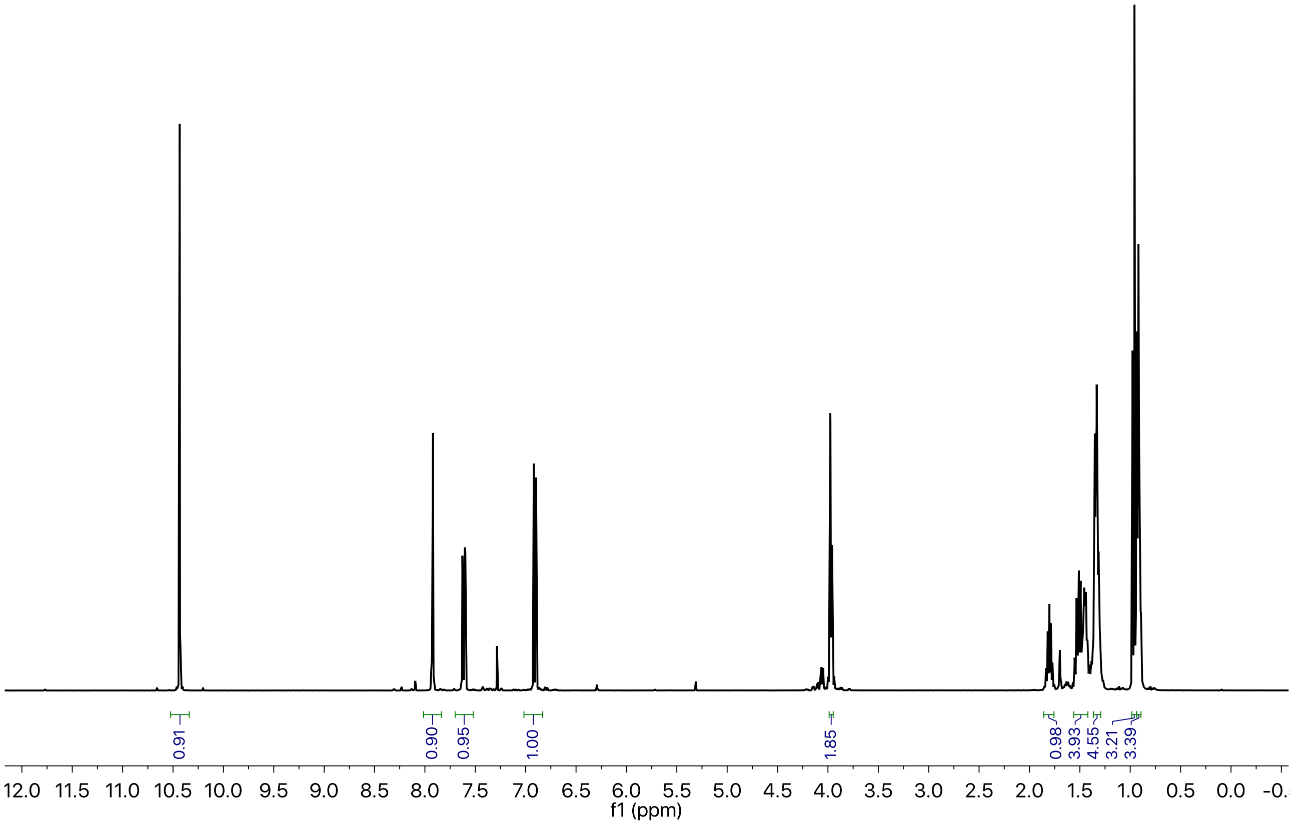

^{1}H-NMR spectrum

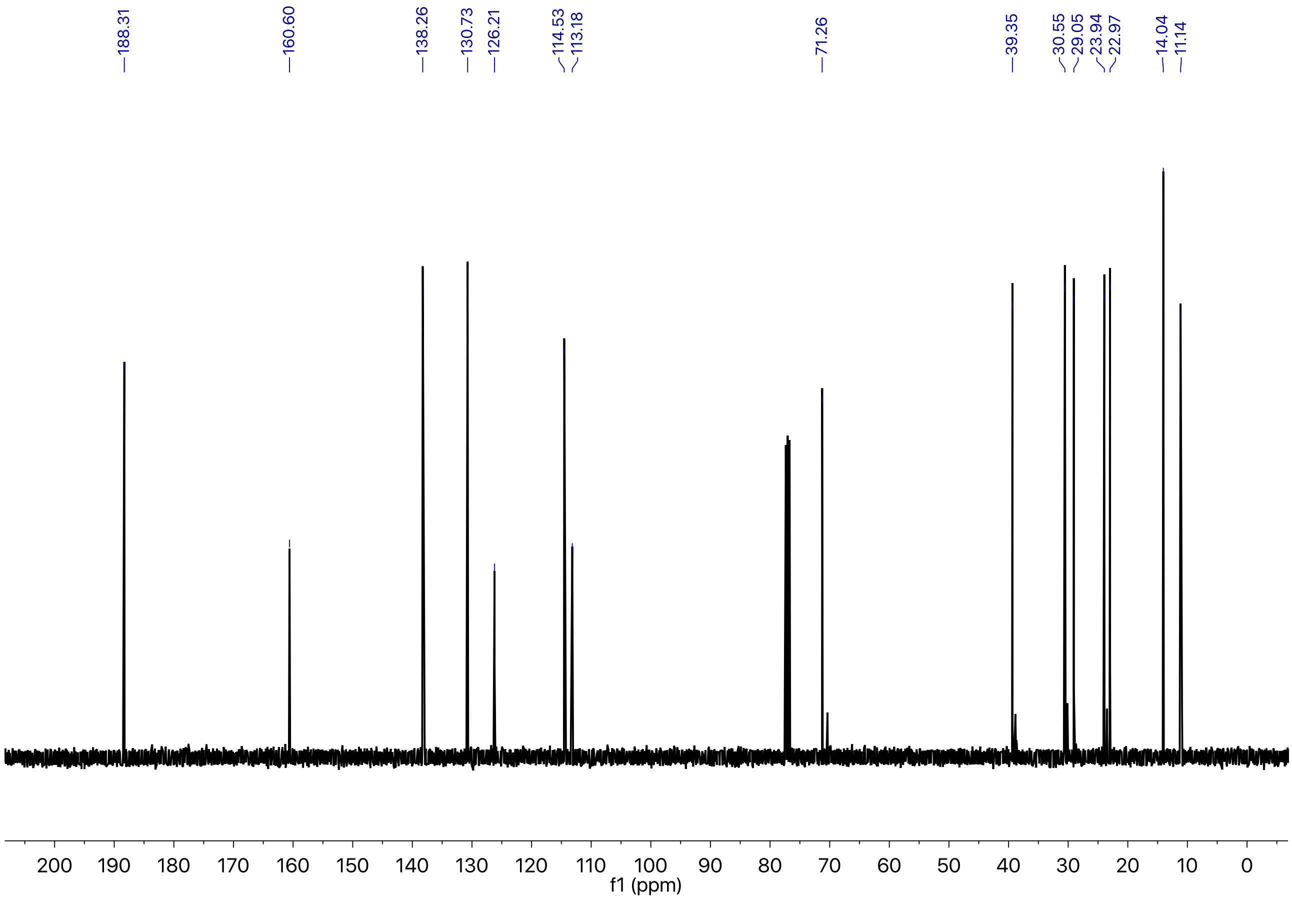

^{13}C-NMR spectrum

2.2 Synthesis of 2.

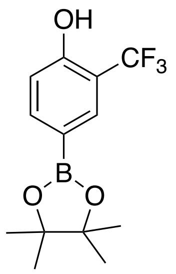

2

4-bromo-2-(trifluoromethyl)phenol (0.5 g, 2.075 mmol, 1 equiv), KOAc (0.611 g, 6.225 mmol, 3 equiv) and Bis(pinacolato)diboron (1.05 g, 4.149 mmol, 2 equiv) were added to dioxane (7 mL); the reaction mixture was degassed bubbling N_2 for 30 min, then Pd(dppf)$_2$Cl$_2$ (0.152 g, 0.2075 mmol, 0.1 equiv) was added and the reaction was refluxed under stirring in N_2 atmosphere overnight. Then the solution was extracted in H_2O/EtOAc, the organic layers were collected, dried over $MgSO_4$, concentrated under vacuum and purified by combiflash, giving 0.450 g of a white solid (75% yield).

^{1}H NMR (400 MHz, $CDCl_3$) δ 8.00 (s, 1H, H3), 7.82 (d, *J* = 7.9 Hz, 1H, H5), 7.39 (bs, 1H, O*H*), 6.92 (d, *J* = 8.1 Hz, 1H, H6), 1.36 (s, 12H, 4C*H*$_3$).
^{13}C NMR (101 MHz, $CDCl_3$) δ 156.84 (q, J^3_{CF} = 1.9 Hz, C1), 139.89 (1C, C5), 133.86 (q, J^3_{CF} = 4.7 Hz, C3), 124,98 (1C, C4) 124.02 (q, J^1_{CF} = 272.3 Hz, *C*F$_3$), 116.46 (q, J^2_{CF} = 30.6 Hz, C2), 116.72 (1C, C6), 84.26 (2C, C_{quat}), 24.72 (8C, *C*H$_3$).
^{11}B NMR (128 MHz, $CDCl_3$) δ 30.42.
^{19}F NMR (376 MHz, $CDCl_3$) δ -61.81.
HRMS (ESI-TOF) (m/z): $[M + H]^+$ calcd for C13H17O3^{11}BF3, 289,1223; found 289,1213.
FT-IR (ATR): vmax 2958, 2930, 2872, 1474, 1458, 1389, 1360, 1326, 1272, 1215, 1164, 1144, 1119, 1098, 1079, 1020, 857, 675 cm^{-1}.

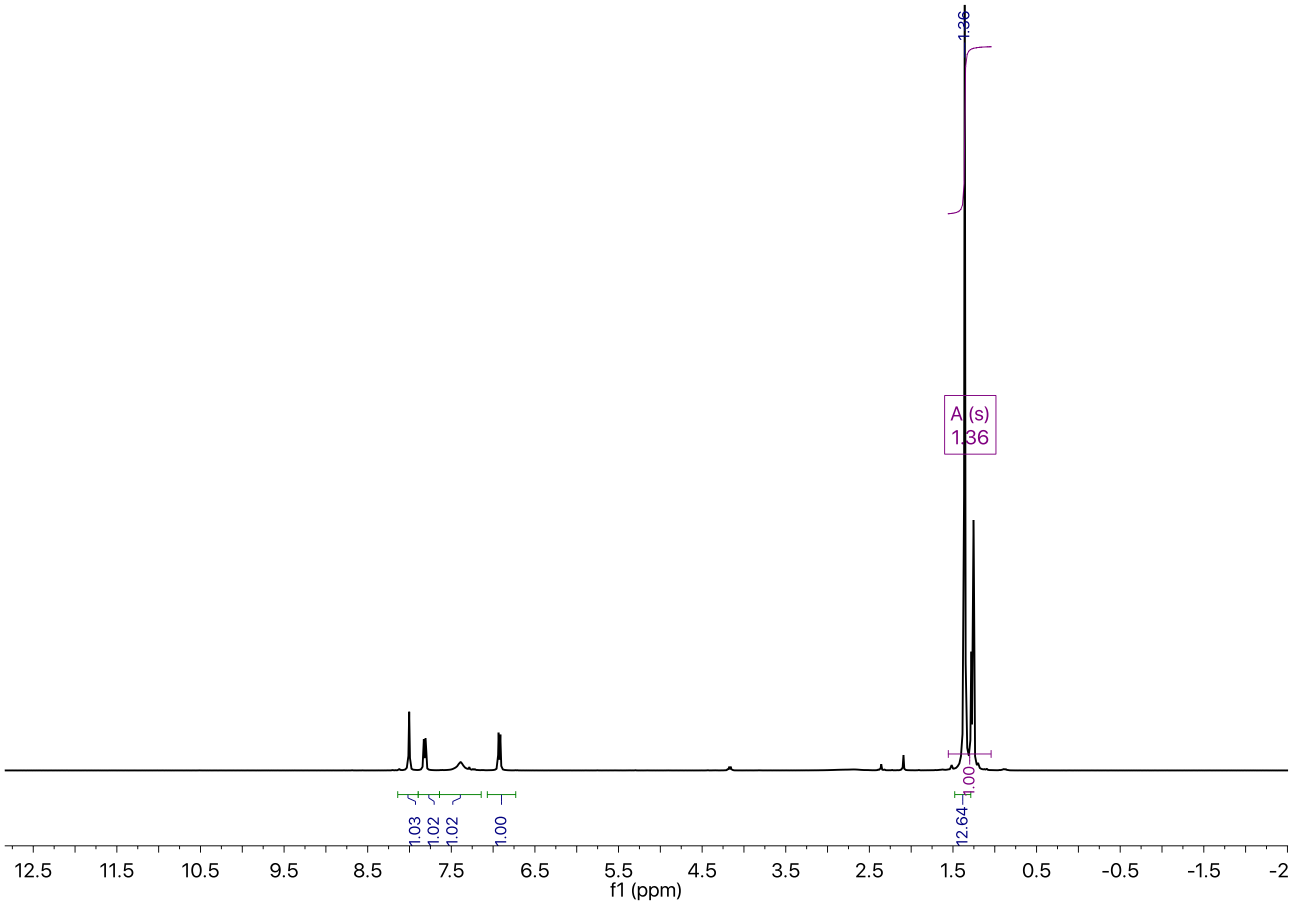

^{1}H-NMR spectrum

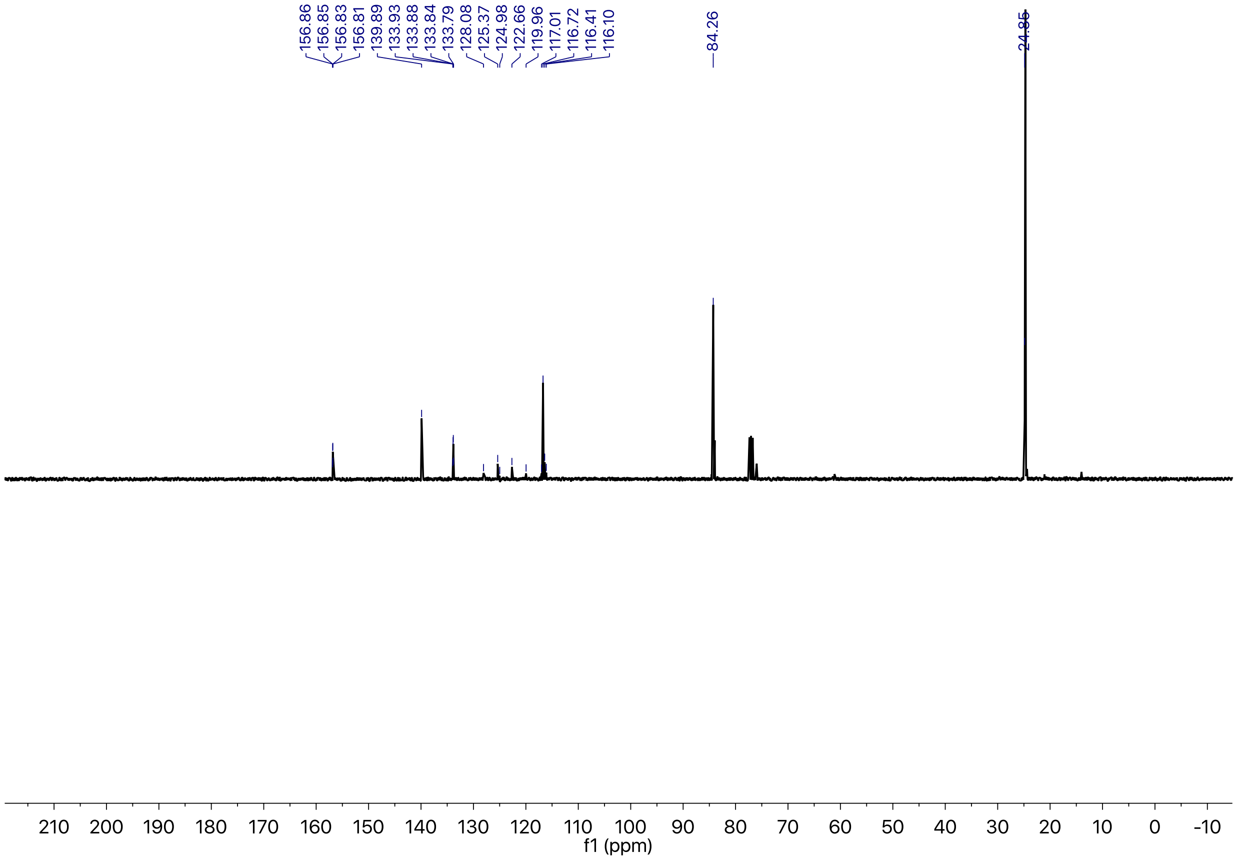

^{13}C-NMR spectrum

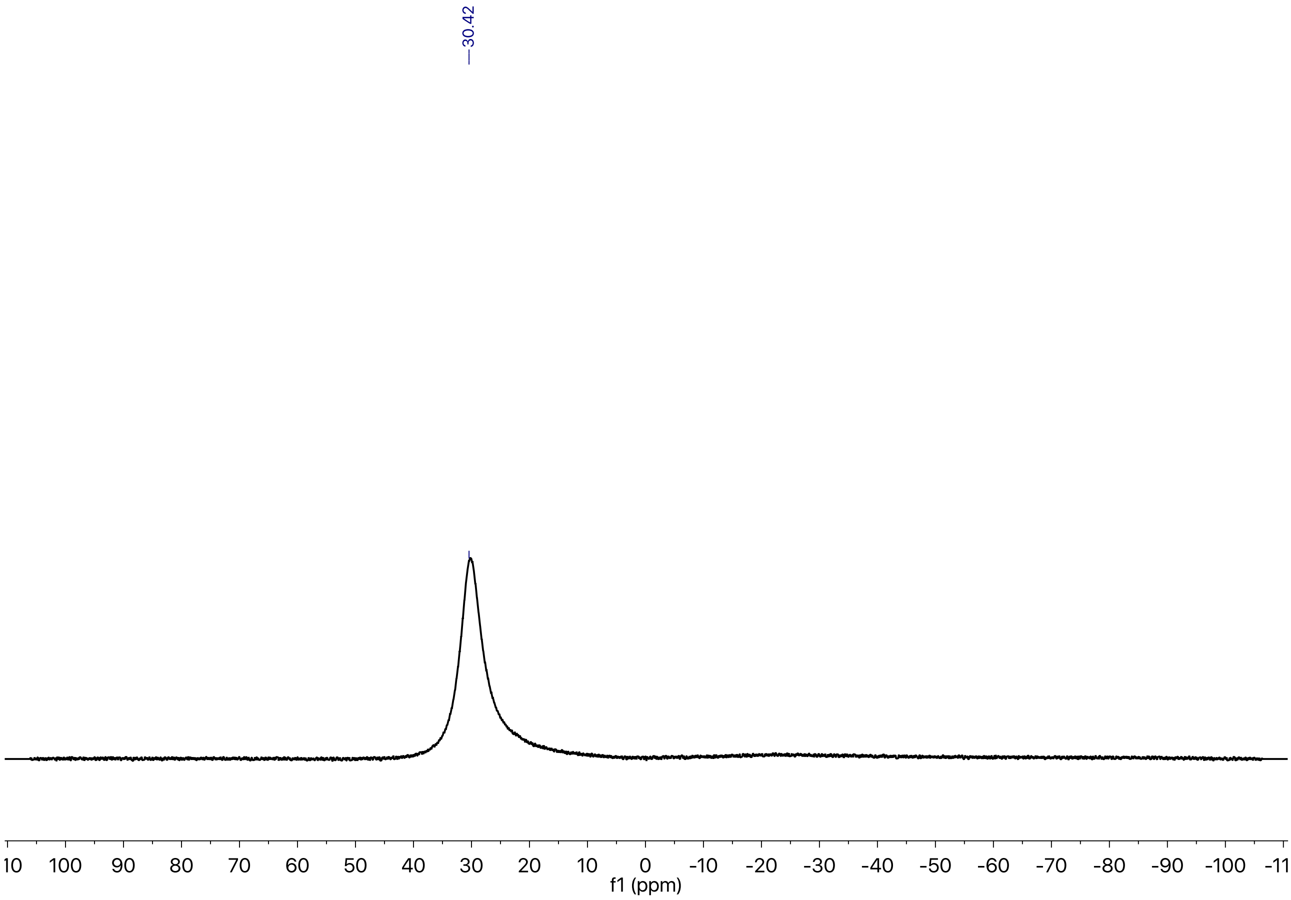

^{11}B-NMR spectrum

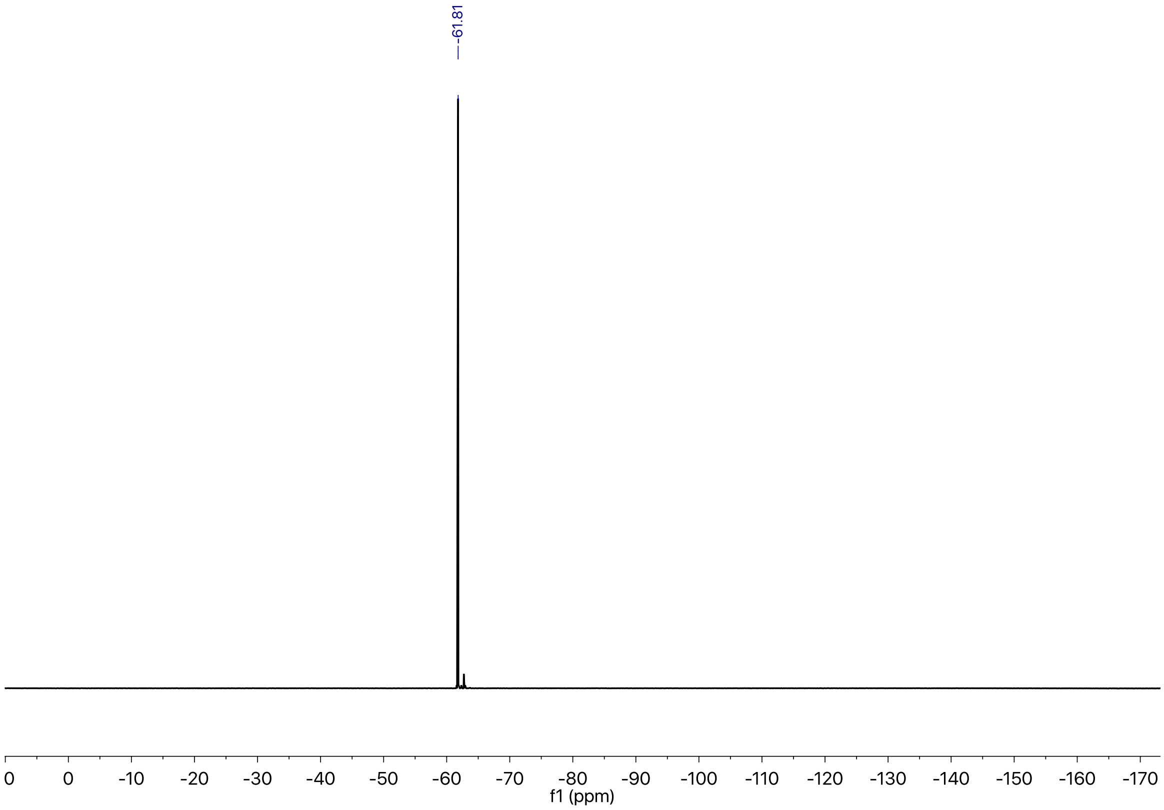

^{19}F-NMR spectrum

2.3 Synthesis of D'.

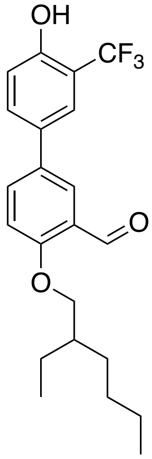

D'

Modifying a reported procedure,[1] in a sealed vial compound **1** (400 mg, 1.277 mmol, 1.2 equiv), compound **2** (306 mg, 1.064 mmol, 1 equiv), KF (250 mg, 4.256 mmol, 4 equiv) and $HP(tBu)_3BF_4$ (10 mg, 0.035 mmol, 0.03 equiv) were suspended in freshly degassed (3 freeze pump thaw cycles) THF (3 mL) and H_2O (1 mL). Then $Pd_2(dba)_3$ (16 mg, 0.0175 mmol, 0.016 equiv) was added and the reaction was stirred under N_2 atmosphere at 80°C under MW irradiation (normal absorbance) for 1 hour and 7 minutes. Then it was extracted in H_2O/EtOAc, the organic phases were collected, dried with $MgSO_4$, concentrated under vacuum and purified by combiflash chromatography with PE:EtOAc (from 9:1 to 8:2). Fractions containing the product were concentrated and then purified again *via* combiflash chromatography, eluting with 100% DCM, giving the pure desired monomer **D'** (355 mg, 85% yields).

^{1}H NMR (400 MHz, $CDCl_3$) δ 10.57 (s, 1H, OH), 8.03 (d, *J* = 2.6 Hz, 1H Ph), 7.74 (dd, *J* = 8.6, 2.6 Hz, 1H Ph), 7.71 (d, *J* = 2.3 Hz, 1H Ph), 7.63 (dd, *J* = 8.5, 2.3 Hz, 1H Ph), 7.13 – 7.03 (m, 2H Ph), 4.05 (dd, *J* = 5.5, 1.1 Hz, 2H, C*H₂*), 1.89 – 1.76 (m, 1H), 1.64 – 1.09 (m, 8), 1.03 – 0.82 (m, 9H).
^{13}C NMR (126 MHz, $CDCl_3$) δ 193.0, 161.4, 154.4, 134.6, 130.8, 125.95, 124.8 (q, J^3_{CF} = 4.9Hz, 1C), 124.51, 123,9 (q, J^1_{CF} = 272.3 Hz, *C*F_3), 122.79, 118.62, 117.1 (q, J^2_{CF} = 30.6 Hz, 1C), 116.9, 110.7, 71.13, 38.3, 30.5, 29,0, 23.8, 20.5, 15.5, 8.9.
^{19}F NMR (376 MHz, $CDCl_3$) δ -61.17.
m.p.: 110.1-111.7 °C
HRMS (ES+): Calculated for $C_{22}H_{25}F_3O_3$ 394.1756 a.m.u., found 394.1747 a.m.u.
FT-IR (ATR): ν_{max} / cm^{-1} 3162, 2944, 2874, 1665, 1610, 1493, 1437, 1335, 1291, 1261, 1237, 1180, 1120, 1051, 814.

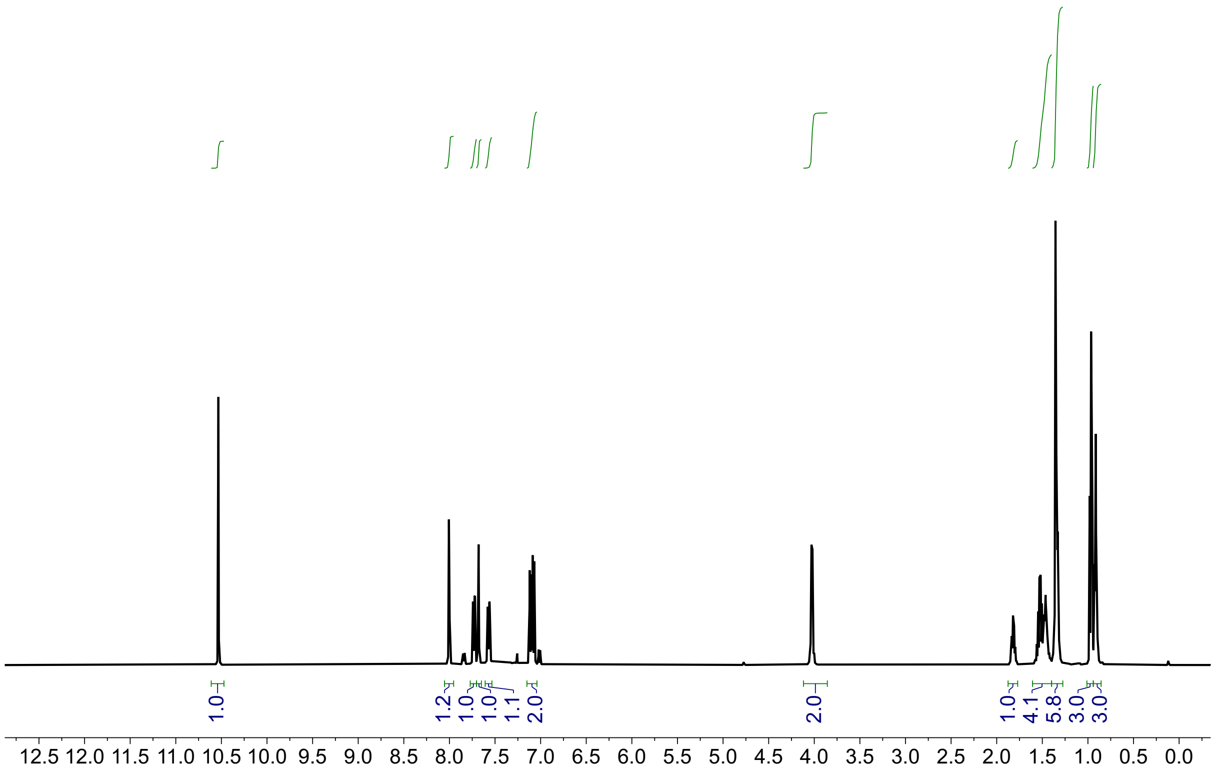

^{1}H-NMR spectrum

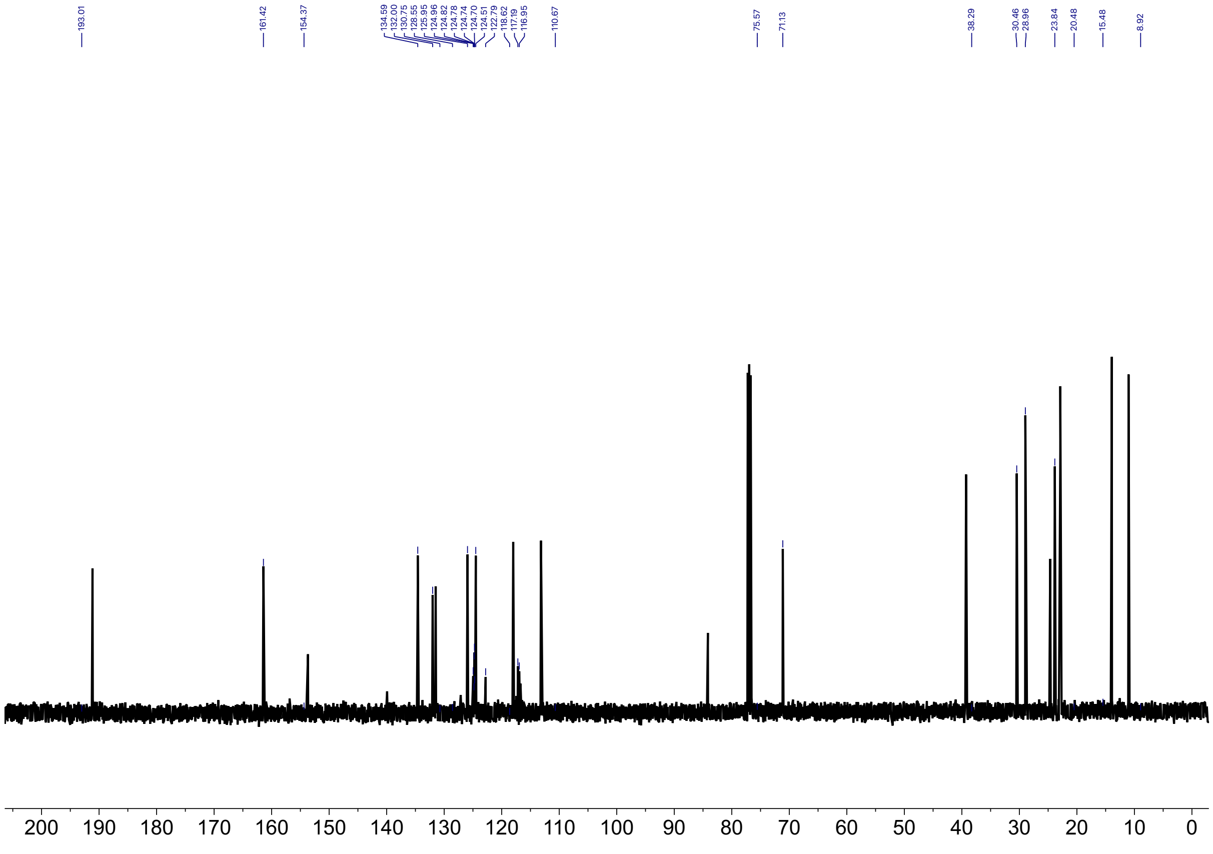

^{13}C-NMR spectrum

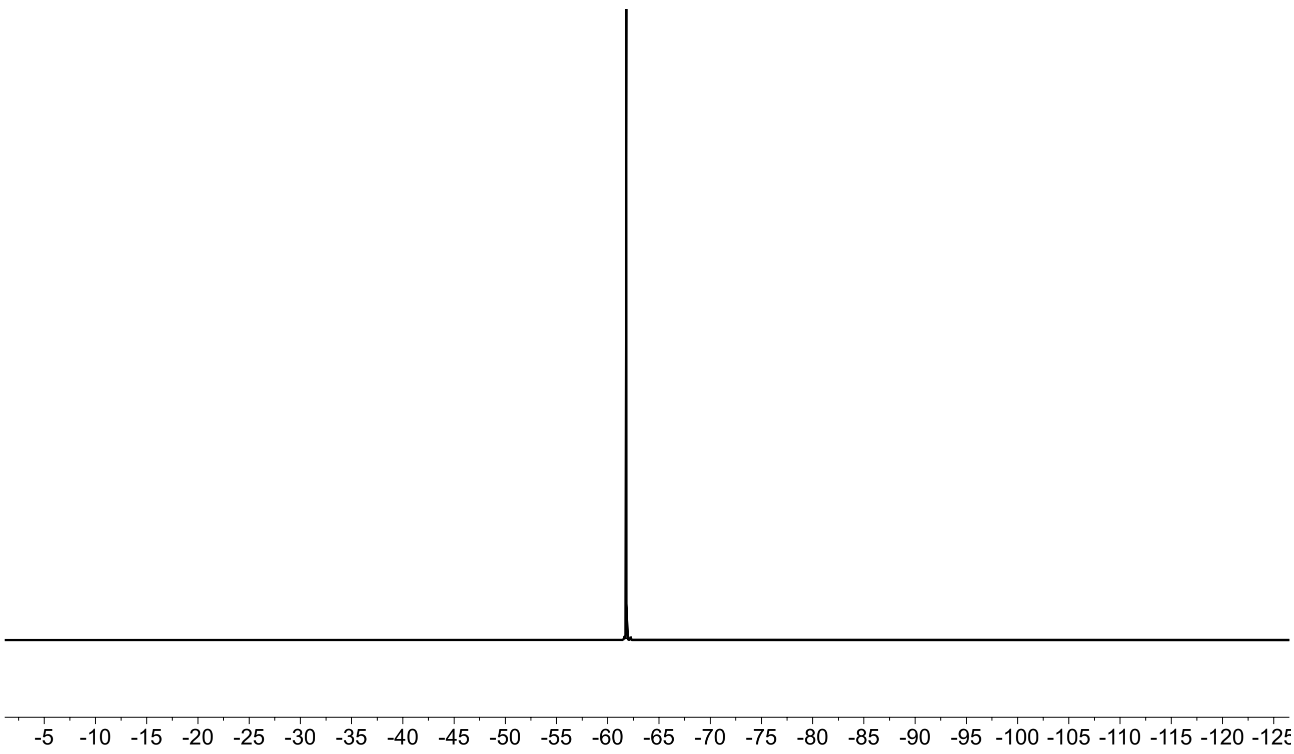

^{19}F-NMR spectrum

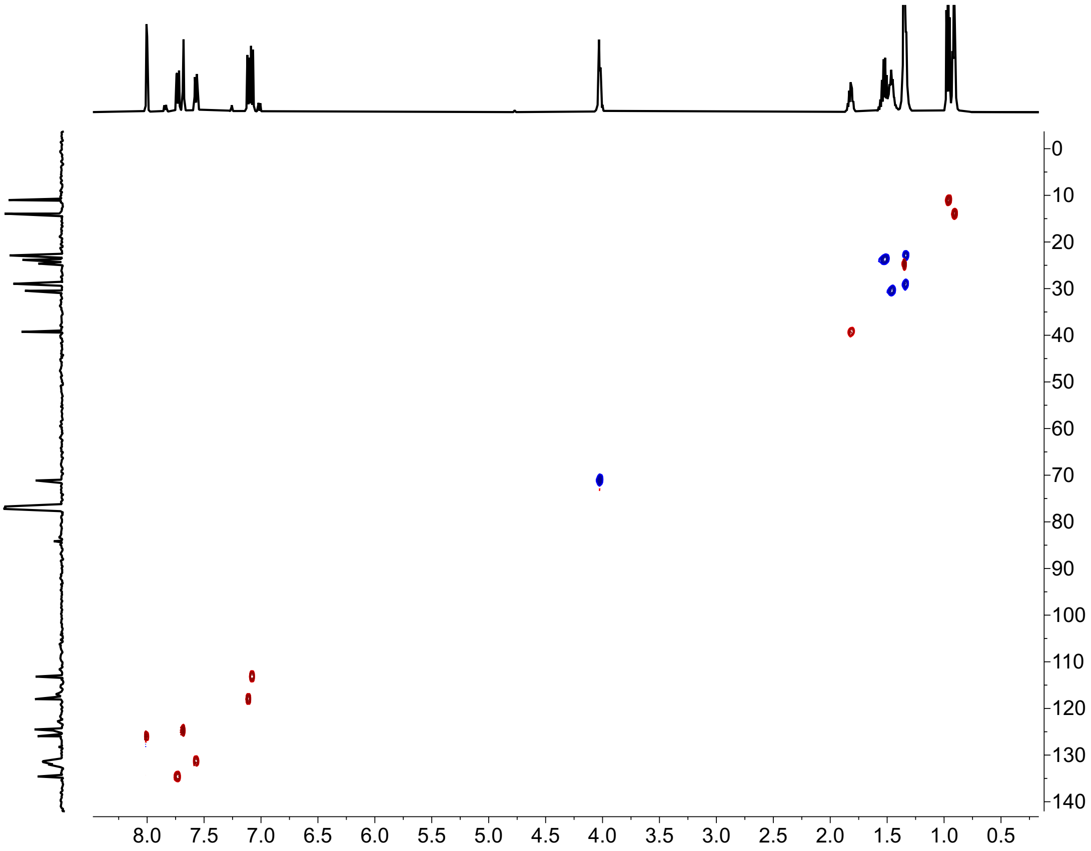

HSQC-NMR spectrum

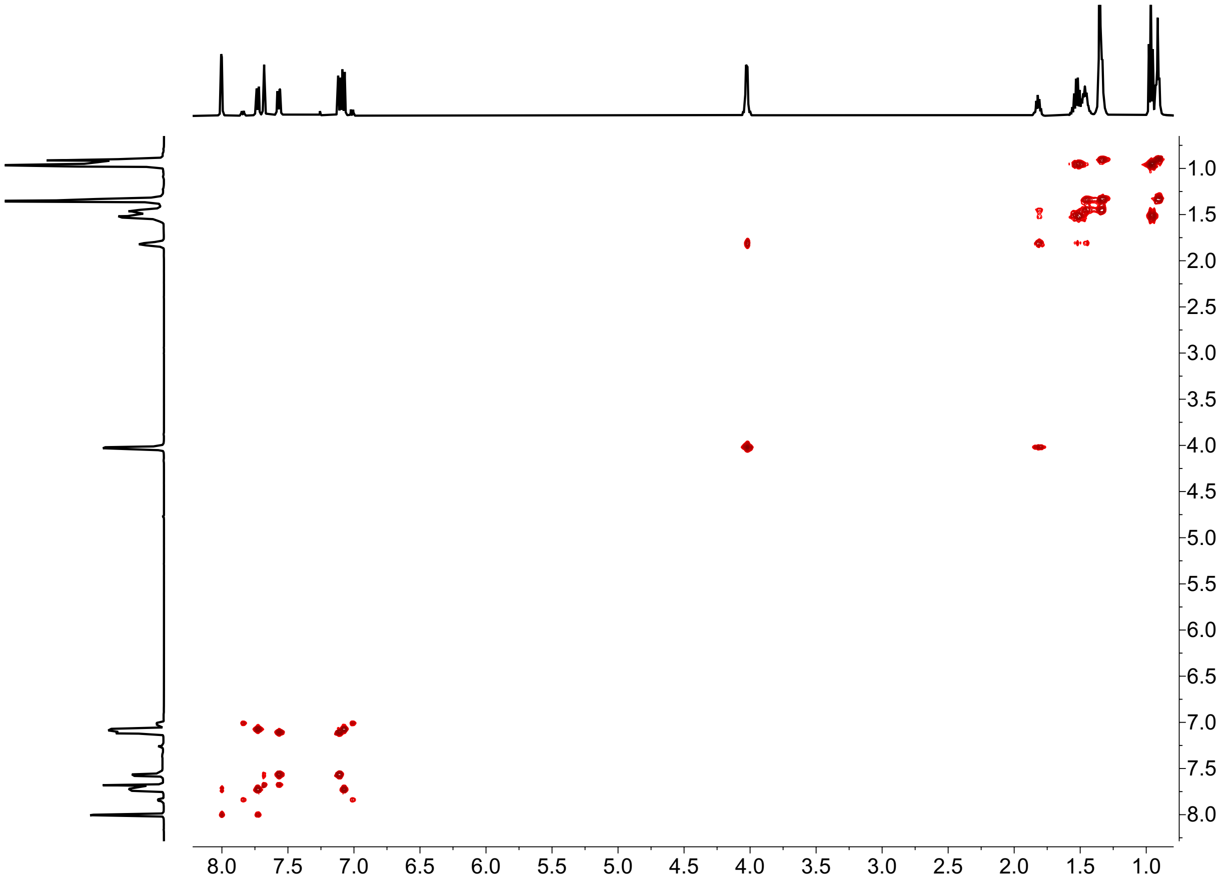

COSY-NMR spectrum

3. Synthesis of monomer D

Monomer **D** was prepared as reported in the following scheme:

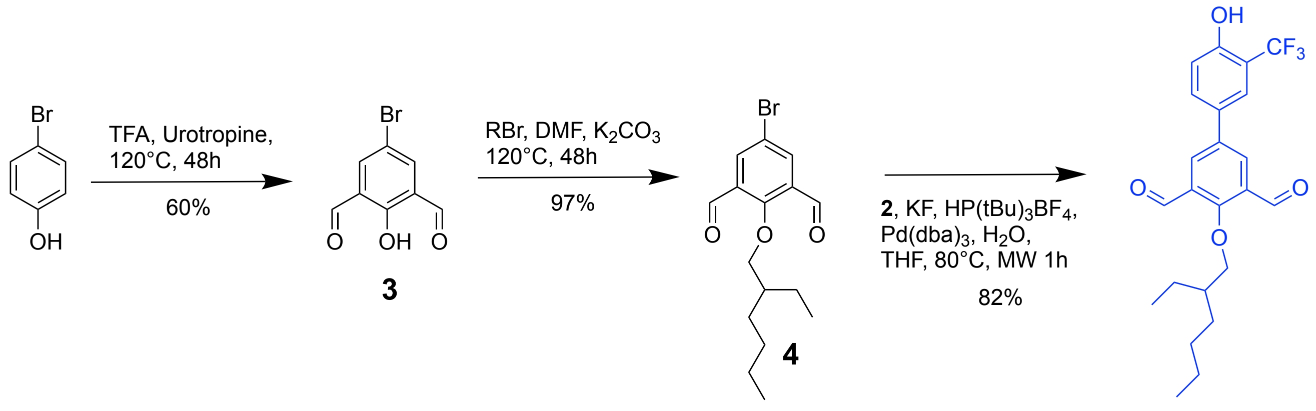

D

3.1 Synthesis of 3.

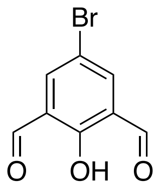

3

According to a reported procedure,[2] hexamethylenetetramine (16 g, 115.60 mmol, 4 equiv) was dissolved in TFA (50 mL) under stirring. 4- Bromophenol (3 g, 17.34 mmol, 1 equiv) was added in one portion and the solution was stirred at 120°C under N_2 for 24 hours. Then other 4- Bromophenol (8 g, 57.80 mmol, 2 equiv) was added and the solution was stirred at 120°C for 48 hours. Then 1N HCl solution (400 mL) was added and the solution stirred at 80°C for 4 hours. Then the solution was cooled to room temperature and the resulting precipitate was collected on a Büchner funnel and washed with HCl 1N. The obtained orange precipitate was purified *via* combiflash (PE:EtOAc 8:2) giving the desired product as a yellow solid (2.3 g, 60% yield). Spectroscopic data are in agreement with those reported in literature.[5]

^{1}H NMR (400 MHz, Chloroform-*d*) δ 11.56 (s, 1H, O*H*), 10.20 (s, 2H, C*H*O), 8.07 (s, 2H, C*H*).
^{13}C NMR (101 MHz, CDCl3) δ 190.63, 162.27, 139.76, 124.61, 112.16.

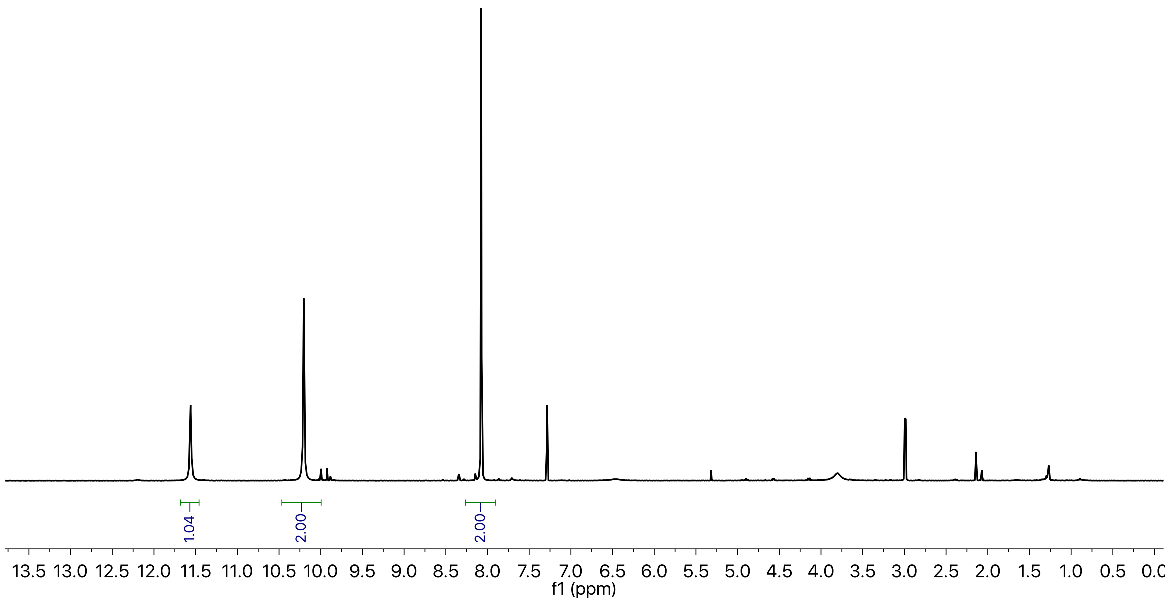

^{1}H-NMR spectrum

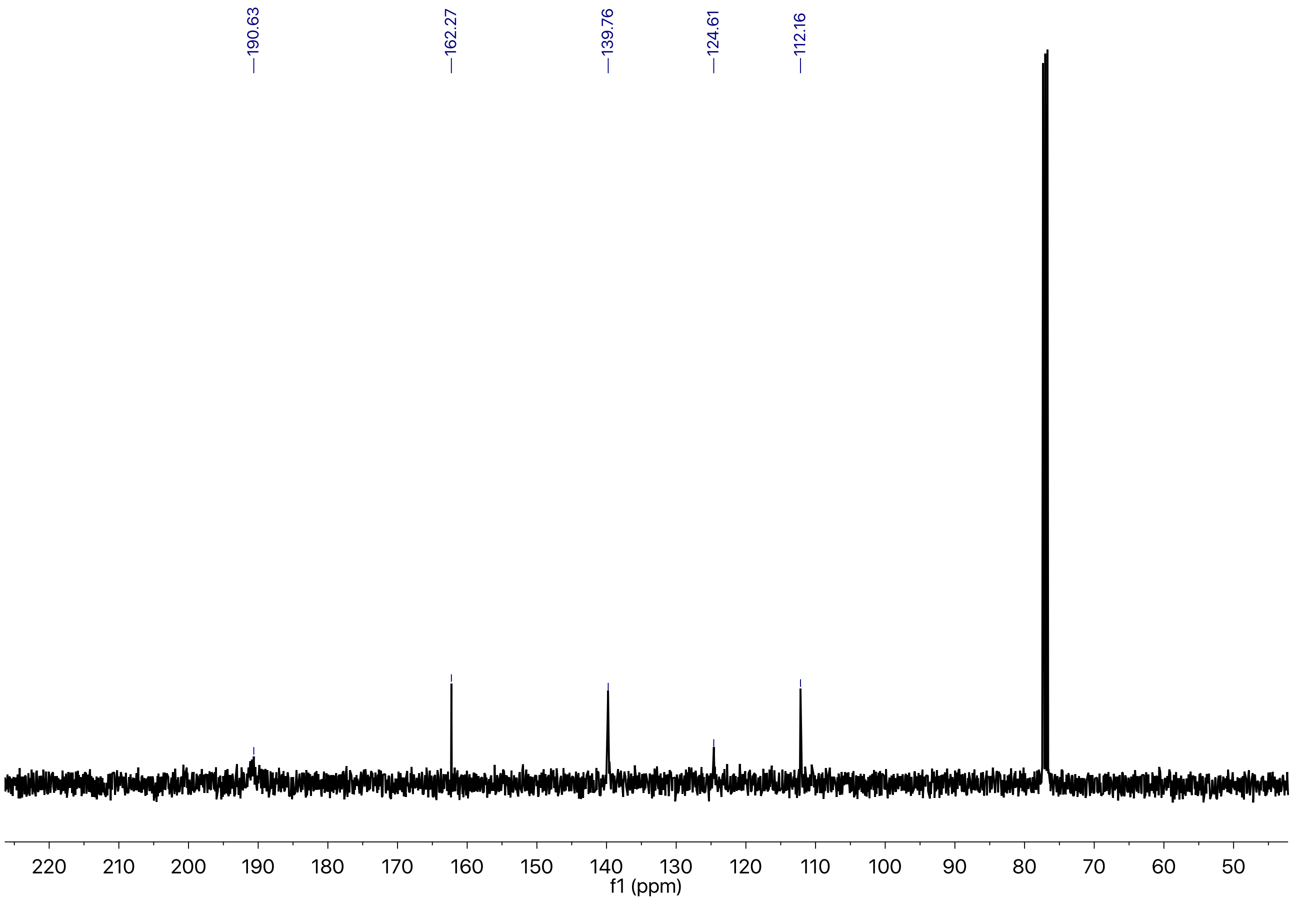

^{13}C-NMR spectrum

3.2 Synthesis of 4.

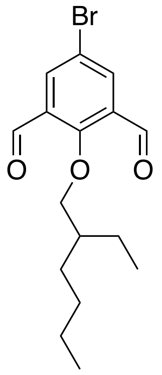

4

Compound **3** (300 mg, 1.310 mmol, 1 equiv) and 3-(bromomethyl)heptane (932 μL, 5.240 mmol, 4 equiv) were dissolved in dry DMF (5 mL); K_2CO_3 anhydrous (724 mg, 5.240 mmol, 4 equiv) was added and the mixture was stirred at 120°C for 48 hours. Then the reaction mixture was extracted 3 times with EtOAc and LiCl 5% solution. The organic layers were collected, dried over $MgSO_4$, concentrated under vacuum and purified by combiflash chromatography, giving 433 mg of a colourless oil (97% yield).

^{1}H NMR (400 MHz, $CDCl_3$) δ 10.31 (s, 2H, C*H*O), 8.13 (s, 2H, C*H* Ph), 4.00 (d, *J* = 5.9 Hz, 2H, C*H₂*O), 1.91 – 1.80 (m, 1H), 1.63 – 1.41 (m, 4H), 1.36 – 1.22 (m, 4H), 0.94 (t, *J* = 7.5 Hz, 3H), 0.91 – 0.86 (m, 3H).
^{13}C NMR (101 MHz, $CDCl_3$) δ 186.98, 163.60, 137.11, 131.62, 118.11, 83.80, 40.66, 30.04, 28.96, 23.50, 22.94, 14.00, 11.03.
HRMS (ESI-TOF) (m/z): $[M + H]^+$ calcd for $C_{16}H_{21}O_3{}^{79}Br_1$, 340,0674; found 340,0661.
FT-IR (ATR): vmax 2959, 2928, 2861, 1741, 1689, 1573, 1441, 1404, 1375, 1259, 1236, 1212, 1147, 963, 945, 897, 872, 713, 623, 544 cm^{-1}.

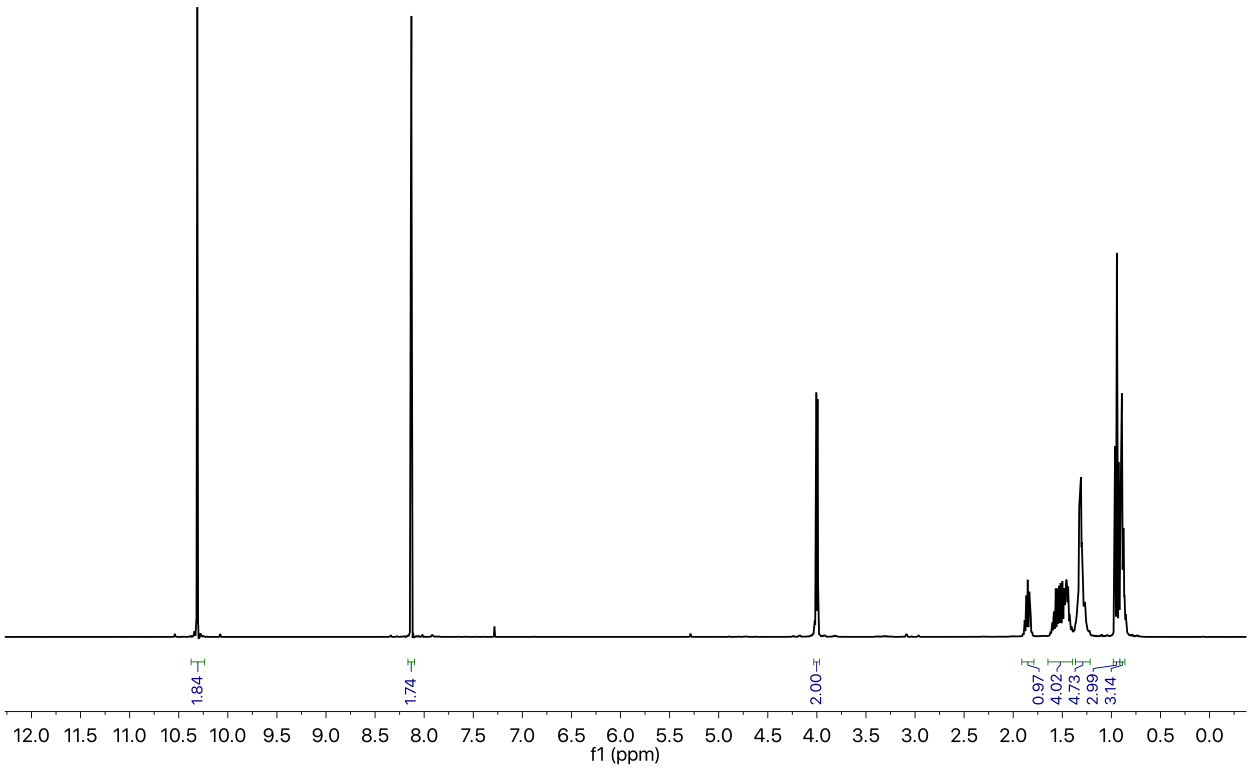

^{1}H-NMR spectrum

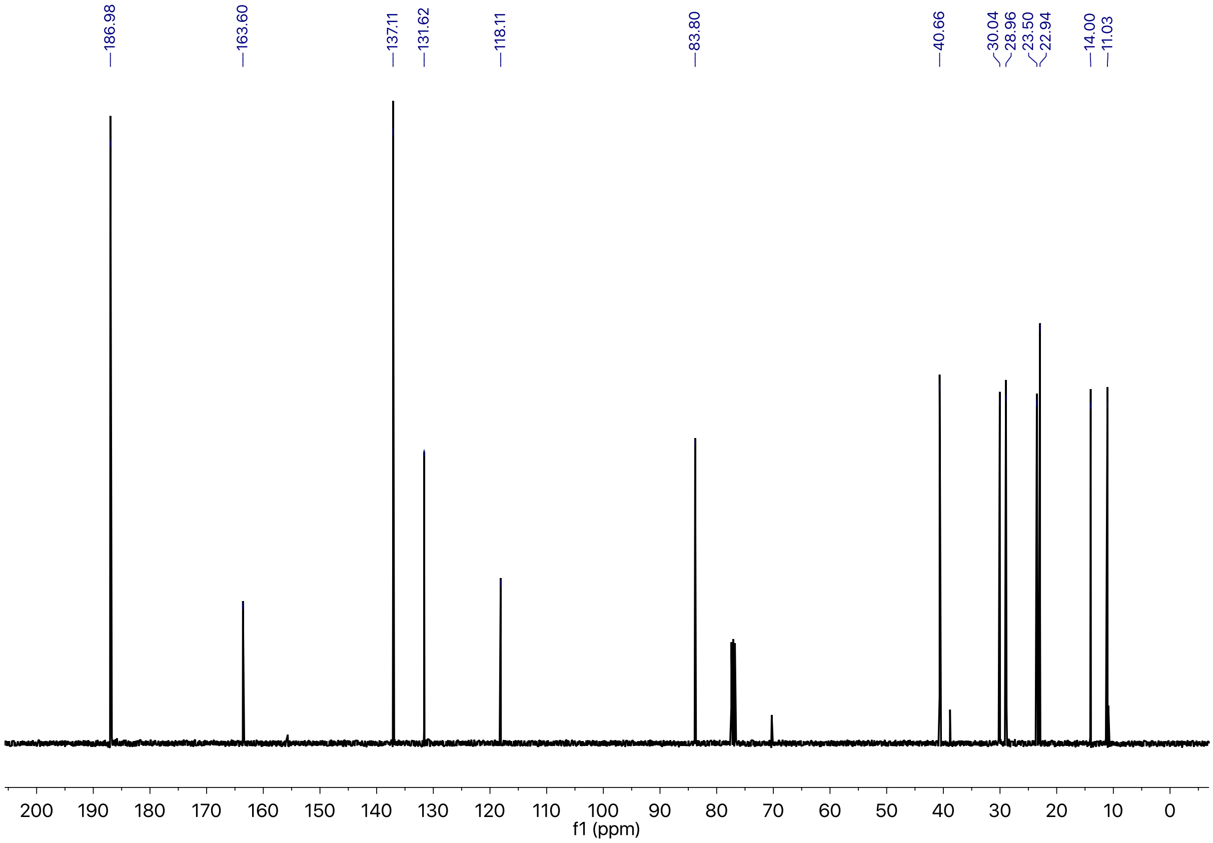

^{13}C-NMR spectrum

3.3 Synthesis of D.

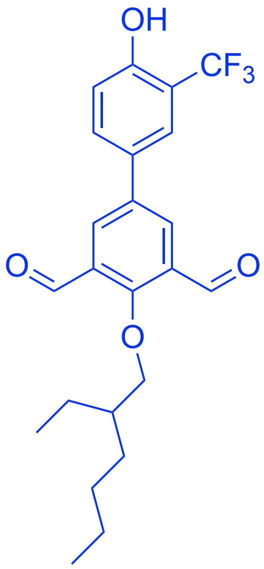

D

Modifying a reported procedure,[3] in a sealed vial compound **4** (500 mg, 1.736 mmol, 1 equiv), compound **2** (711 mg, 2.783 mmol, 1.2 equiv), KF (303 mg, 5.208 mmol, 3 equiv) and $HP(tBu)_3BF_4$ (10 mg, 0.035 mmol, 0.02 equiv) were suspended in freshly degassed (3 freeze pump thaw cycles) THF (2.8 mL) and H_2O (1 mL). Then $Pd_2(dba)_3$ (16 mg, 0.018 mmol, 0.016 equiv) was added and the reaction was stirred under N_2 atmosphere at 80°C under MW irradiation (normal absorbance) for 1 hour and 5 minutes. Then it was extracted in H_2O/EtOAc, the organic phases were collected, dried with $MgSO_4$, concentrated under vacuum and purified by combiflash chromatography with PE:EtOAc (from 9:1 to 8:2). Fractions containing the product were concentrated and then purified again *via* combiflash chromatography, eluting with 100% DCM, giving the pure desired monomer **D** (605 mg, 82% yields).

^{1}H NMR (400 MHz, $CDCl_3$) δ 10.49 (s, 2H), 8.29 (s, 2H), 7.77 (d, *J* = 2.4 Hz, 1H), 7.70 (dd, *J* = 8.5, 2.5 Hz, 1H), 7.11 (d, *J* = 8.6 Hz, 1H), 6.05 (s, 1H), 4.08 (d, *J* = 5.9 Hz, 2H), 1.99 – 1.85 (m, 1H), 1.69 – 1.45 (m, 5H), 1.37 (h, *J* = 3.5 Hz, 4H), 1.00 (t, *J* = 7.5 Hz, 3H), 0.98 – 0.91 (m, 3H).
^{13}C NMR (126 MHz, $CDCl_3$) δ 189.4, 164.8, 154.0, 136.1, 132.4, 131.6, 130.5, 130.3, 125,3 (q, J^3_{CF} = 4.9Hz, 1C), 123,7 (q, J^1_{CF} = 272.3 Hz, CF_3), 118.4, 117,2 (q, J^2_{CF} = 30.6 Hz, 1C), 83.8, 40.6, 30.0, 28.9, 23.5, 22.9, 13.9, 10.9.
^{19}F NMR (471 MHz, $CDCl_3$) δ -61.55.
HRMS (ASAP+) (m/z): $[M + H]^+$ calcd for $C_{23}H_{26}O_4F_3$, 423,1783; found 423,1773.
FT-IR (ATR): ν_{max} / cm^{-1} 3054, 2929, 2872, 2307, 1688, 1622, 1575, 1519, 1461, 1443, 1401, 1381, 1338, 1295, 1264, 1235, 1189, 1135, 1072, 1055, 896, 731, 703.

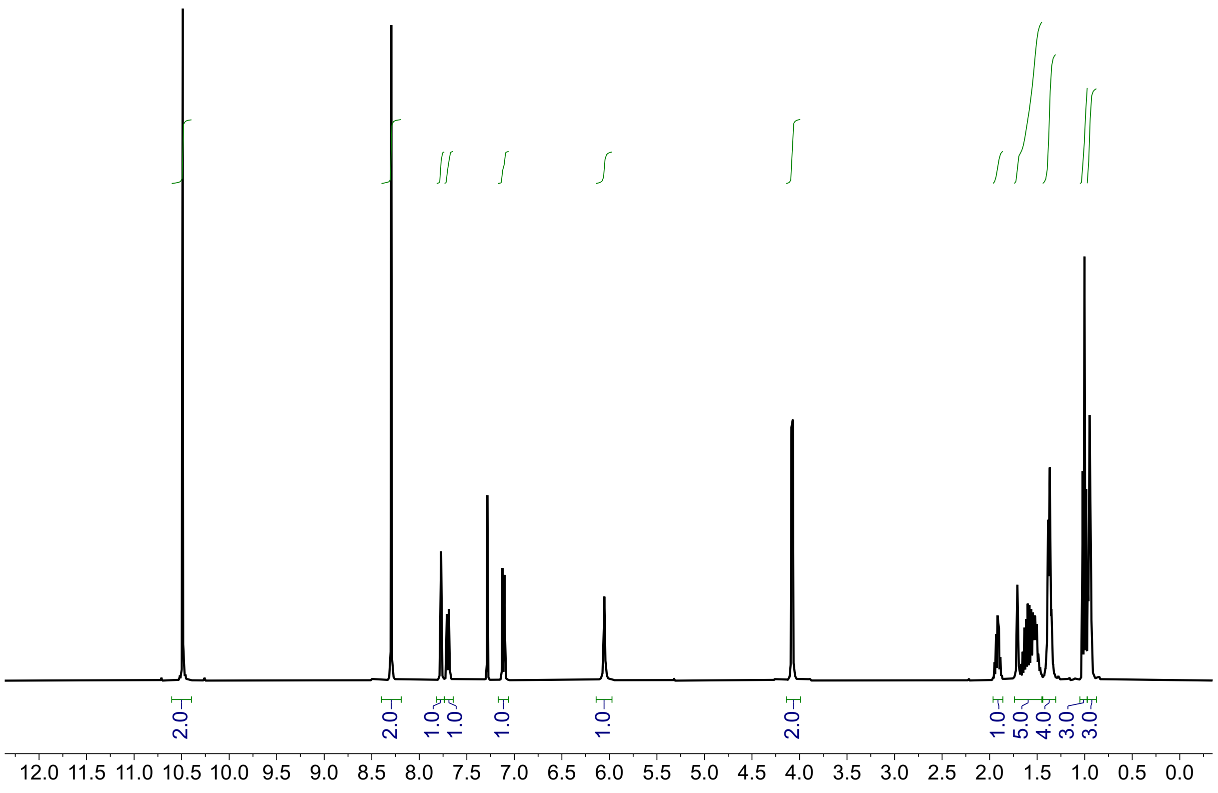

^{1}H-NMR spectrum

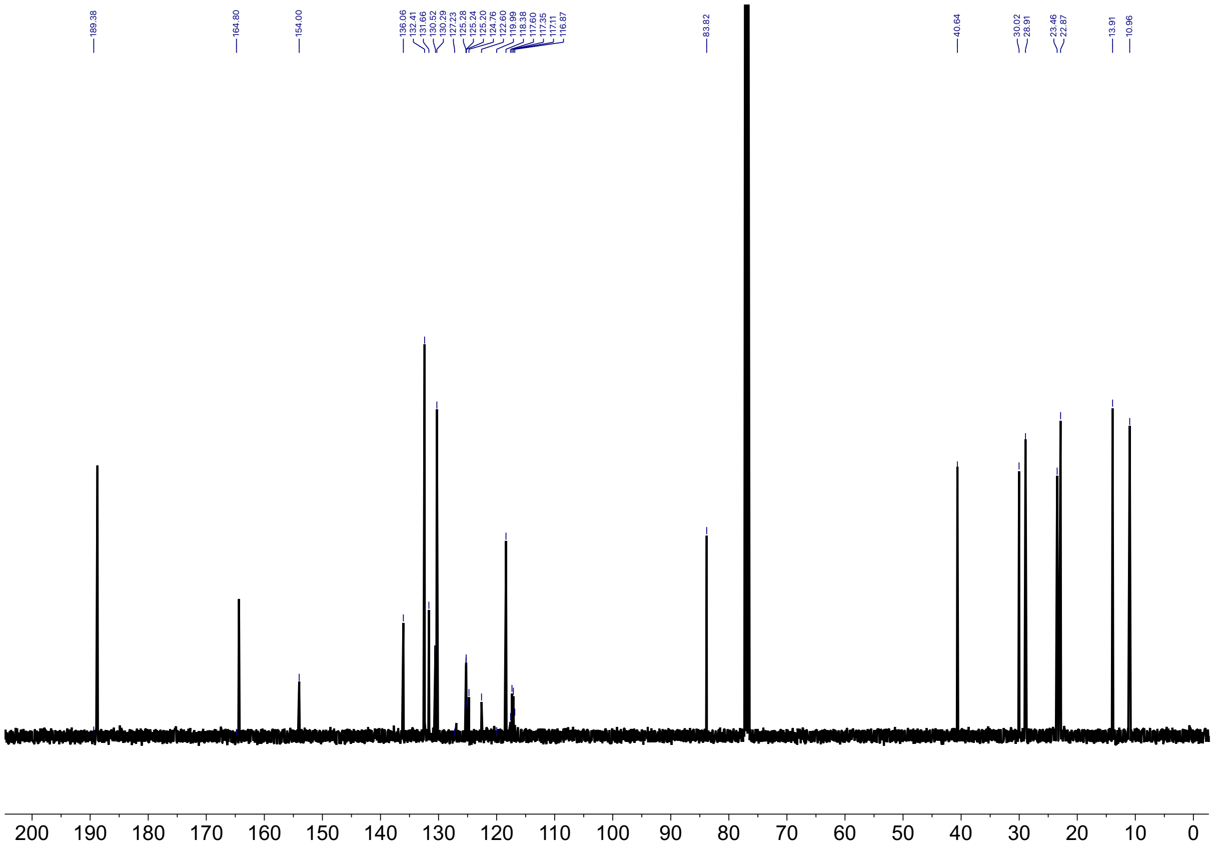

^{13}C-NMR spectrum

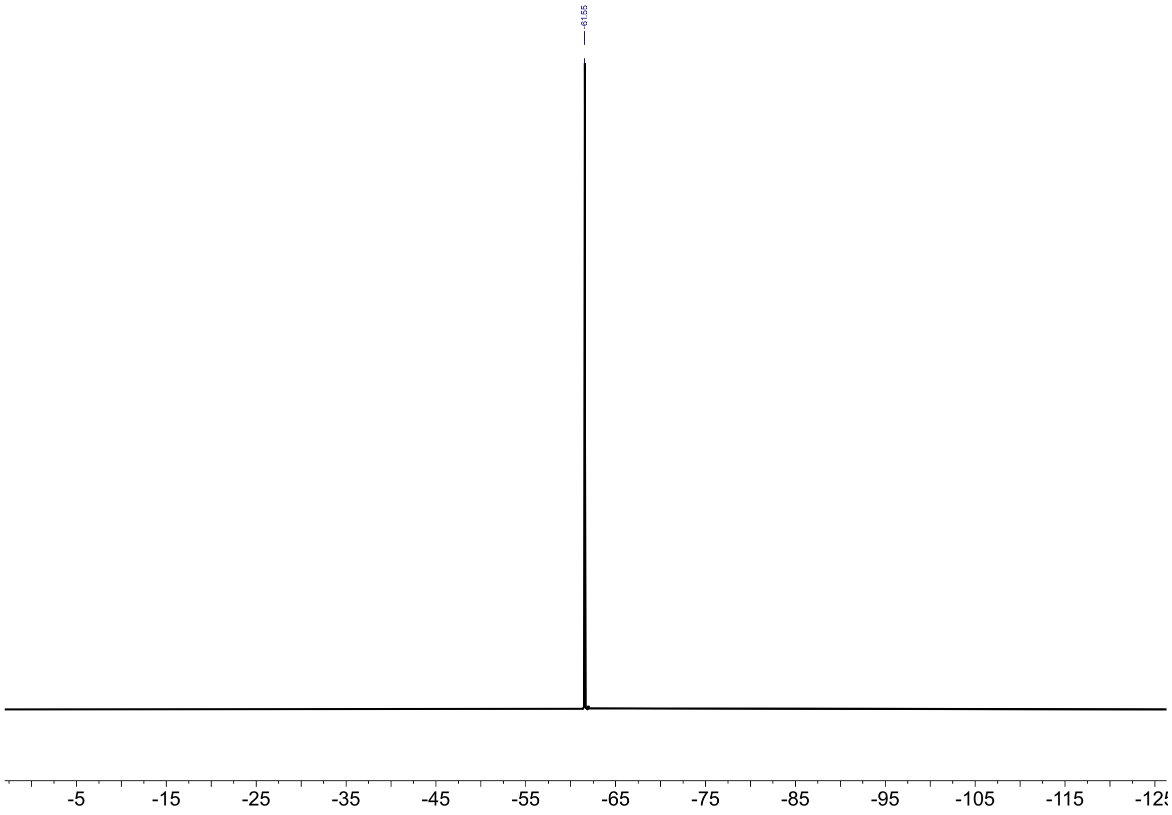

^{19}F-NMR spectrum

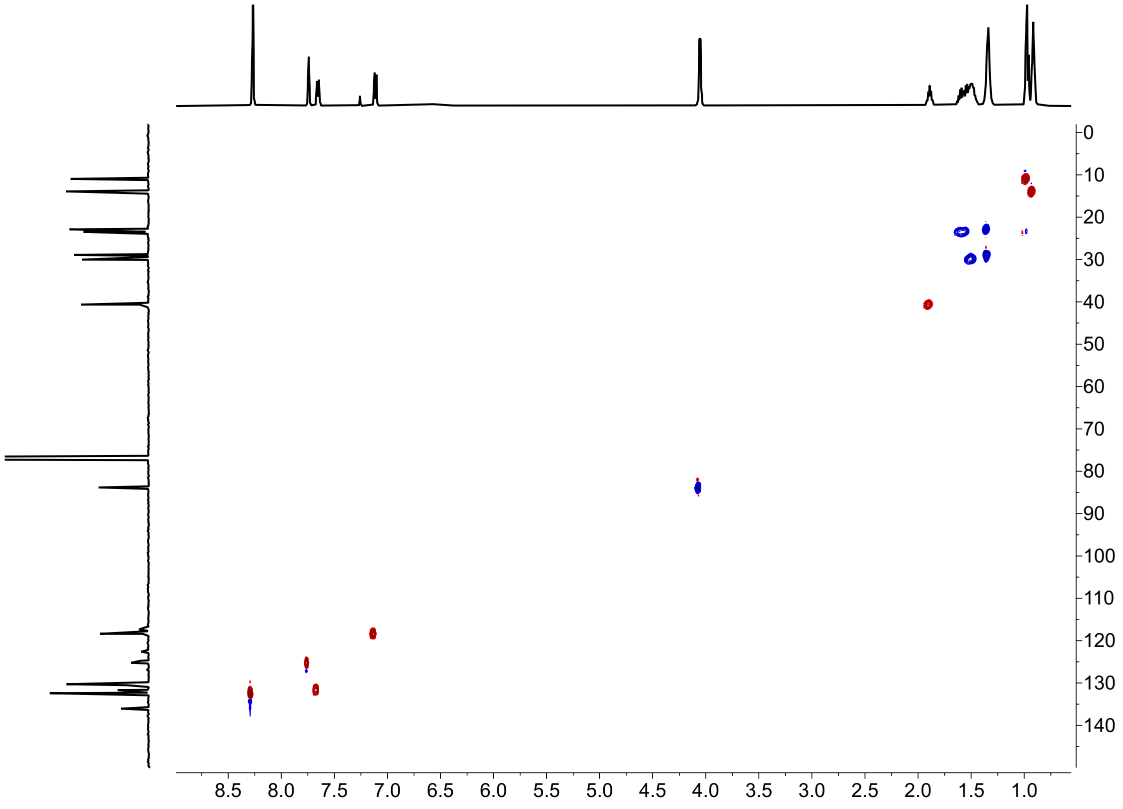

HSQC-NMR spectrum

4. Synthesis of monomer A’

Monomer **A’** was prepared as reported in the following scheme:

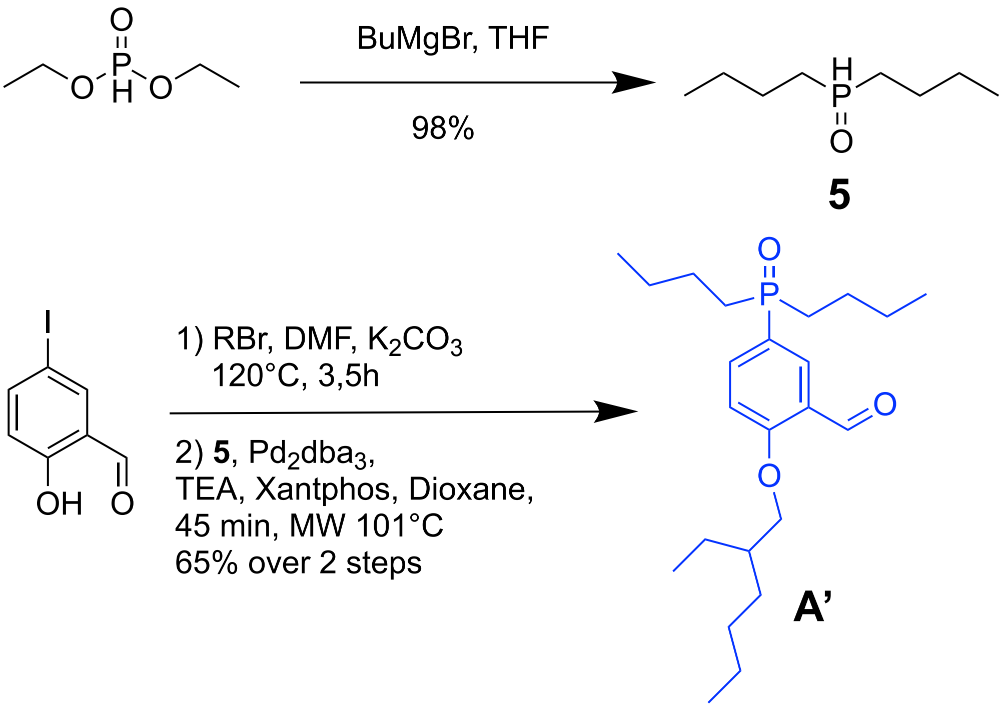

4.1 Synthesis of 5.

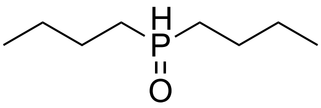

5

Compound **5** was synthesized following a reported procedure.[4] A flask, evacuated/N_2 filled (3x), was charged with n-BuMgCl (2 M in Et_2O, 38.78 mL, 77.57 mmol) and the solution cooled to 0°C. A solution of diethylphosphite (3.331 mL, 25.86 mmol) in 50 mL THF was then added dropwise over 15 minutes. The mixture was aged 15 minutes at 0°C, then the bath was removed, and the mixture stirred two hours at room temperature, then cooled again to 0°C. 0.1 M HCl solution (50 mL) was added dropwise over 20 minutes. To the obtained gel was added CH_2Cl_2 (50 mL), and the mixture agitated well for 5 minutes. The resultant mixture was then filtered through a Celite pad, washing the pad with CH_2Cl_2. The filtrate phases were separated, and the organic phase combined with the first organic phase, dried ($MgSO_4$), and the solvents removed in vacuum. The residue was

azeotroped with hexane (2x30 mL) causing a precipitate that correspond to dibutylphosphine oxide (4.195 g, quantitative). Spectroscopic data were in agreement with the reported,[4] and this compound was used in the following step without further purification.

^{1}H NMR (400.0 MHz, $CDCl_3$): δ_H = 7.49 – 6.25 (dm, *J* = 446 Hz, 1H), 1.94 – 1.50 (m, 8H), 1.50 – 1.34 (m, 4H), 0.93 (t, *J* = 7.3 Hz, 6H).
^{13}C NMR (100.6 MHz, $CDCl_3$): δ_C = 28.25, 27.60, 23.78 (dd, *J* = 9.1, 5.2 Hz), 13.56.
^{31}P NMR (162.0 MHz, $CDCl_3$): δ_P = 35.03
HRMS (ES+): Calculated for $C_8H_{20}OP$ 163.1252 a.m.u., found 163.1258 a.m.u.

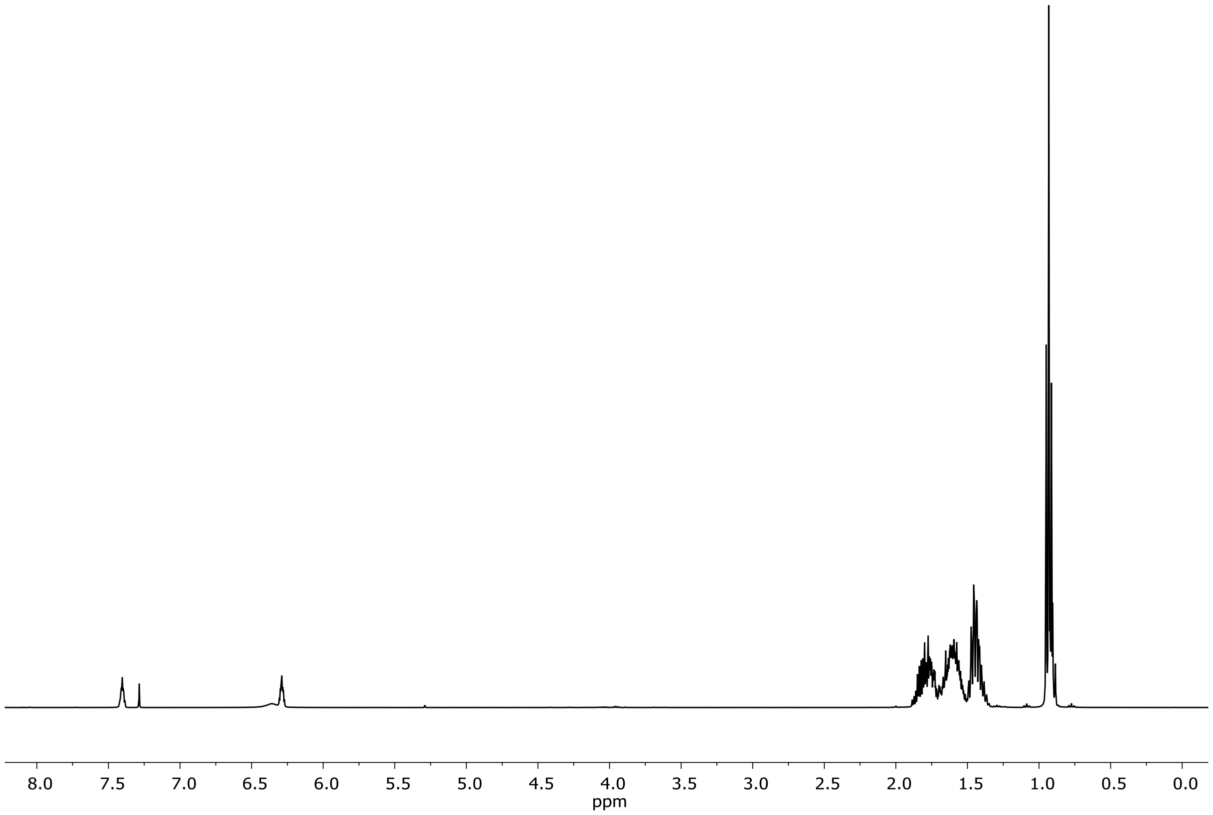

^{1}H-NMR spectrum

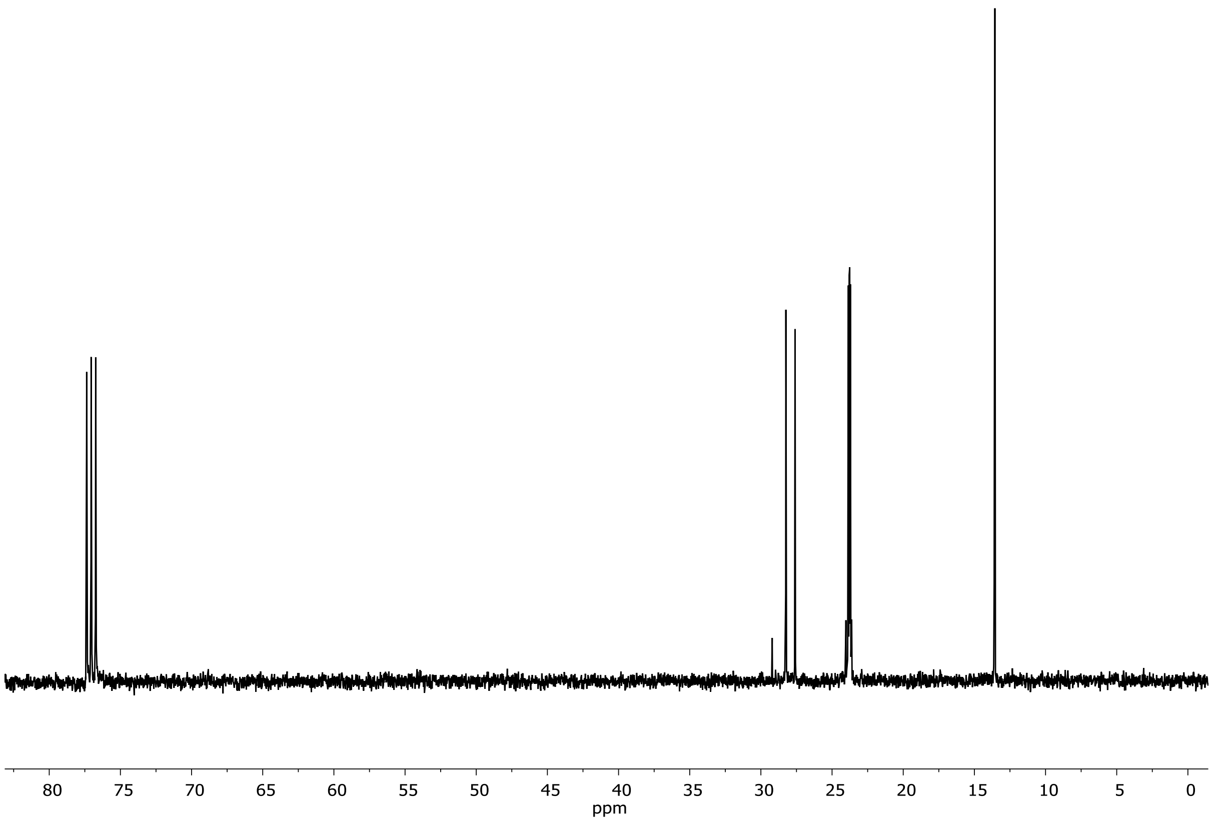

^{13}C-NMR spectrum

4.2 Synthesis of A'.

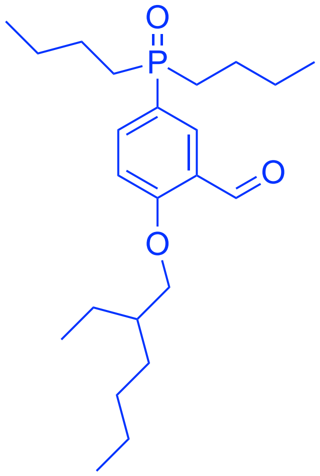

2-hydroxy-5-iodobenzaldehyde (1.5 g, 6.048 mmol, 1 equiv) and 3-(bromomethyl)heptane (4.3 mL, 24.192 mmol, 4 equiv) were dissolved in dry DMF (15 mL); K_2CO_3 anhydrous (3.3 g, 24.192 mmol, 4 equiv) was added and the mixture was stirred at 120°C for 3.5 hours. Then the reaction mixture was extracted 3 times with EtOAc and LiCl 5% solution. The organic layers were collected, dried over

$MgSO_4$, concentrated under vacuum and purified by combiflash chromatography (PE:EtOAc) , giving 1.5 g of a colorless oil, which is freshly used for the next synthetic step, because of its instability. Thus, the obtained alkylated iodobenzaldehyde (1.5 g, 4.164 mmol, 1 equiv) was mixed with phosphine oxide **5** (1 g, 6.246 mmol, 1.5 equiv), Xantphos (241 mg, 0.416 mmol, 0.1 equiv) and Pd_2dba_3 (241 mg, 0.416 mmol, 0.1 equiv); finally, previously degassed dioxane (30 mL, 3 freeze pump thaw cycles) and trimethylamine (1.7 mL, 12.492 mmol, 3 equiv) were added, and the solution was stirred under nitrogen atmosphere, in dark conditions, for 45 minutes. Then the mixture was filtered through Celite, washing the pad with EtOAc. The filtrate was collected, the solvent evaporated, and the obtained residue was purified using combiflash chromatography EtOAc: MeOH (from 0% to 3%) and then DCM: MeOH (from 0% to 3%), affording 1.1 g of the desired monomer **A'** (67% yield over 2 steps).

^{1}H NMR (500 MHz, $CDCl_3$) δ 10.36 (s, 1H), 7.92 (t, *J* = 10.5 Hz, 1H), 7.79 (dd, *J* = 10.3, 2.4 Hz, 1H), 7.01 (d, *J* = 8.6 Hz, 1H), 3.90 (d, *J* = 5.5 Hz, 2H), 1.88 – 1.76 (m, 2H), 1.75 – 1.61 (m, 3H), 1.49 – 1.11 (m, 16H), 0.79 (t, *J* = 7.4 Hz, 3H), 0.72 (t, *J* = 7.1 Hz, 3H), 0.69 (t, *J* = 7.2 Hz, 6H).
^{13}C NMR (126 MHz, $CDCl_3$) δ 188.65, 163.48 (d, *J* = 2.6 Hz), 138.86 (d, *J* = 8.3 Hz), 129.42 (d, *J* = 12.0 Hz), 124.43 (d, *J* = 10.9 Hz), 123.94 (d, *J* = 95.5 Hz), 112.70 (d, *J* = 10.7 Hz), 70.95, 39.08, 30.30, 29.77, 29.22, 28.79, 23.85, 23.73, 23.69, 23.24, 23.21, 22.70, 13.76, 13.31, 10.90.
^{31}P NMR (202 MHz, $CDCl_3$) δ 40.41.
HRMS (ES+): Calculated for $C_{23}H_{40}O_3P$ 395.2715 a.m.u., found 395.2696 a.m.u.
FT-IR (ATR): ν_{max} / cm^{-1} 2957, 2929, 2861, 1684, 1594, 1489, 1463, 1385, 1280, 1242, 1193, 1168, 1087, 1007, 918.

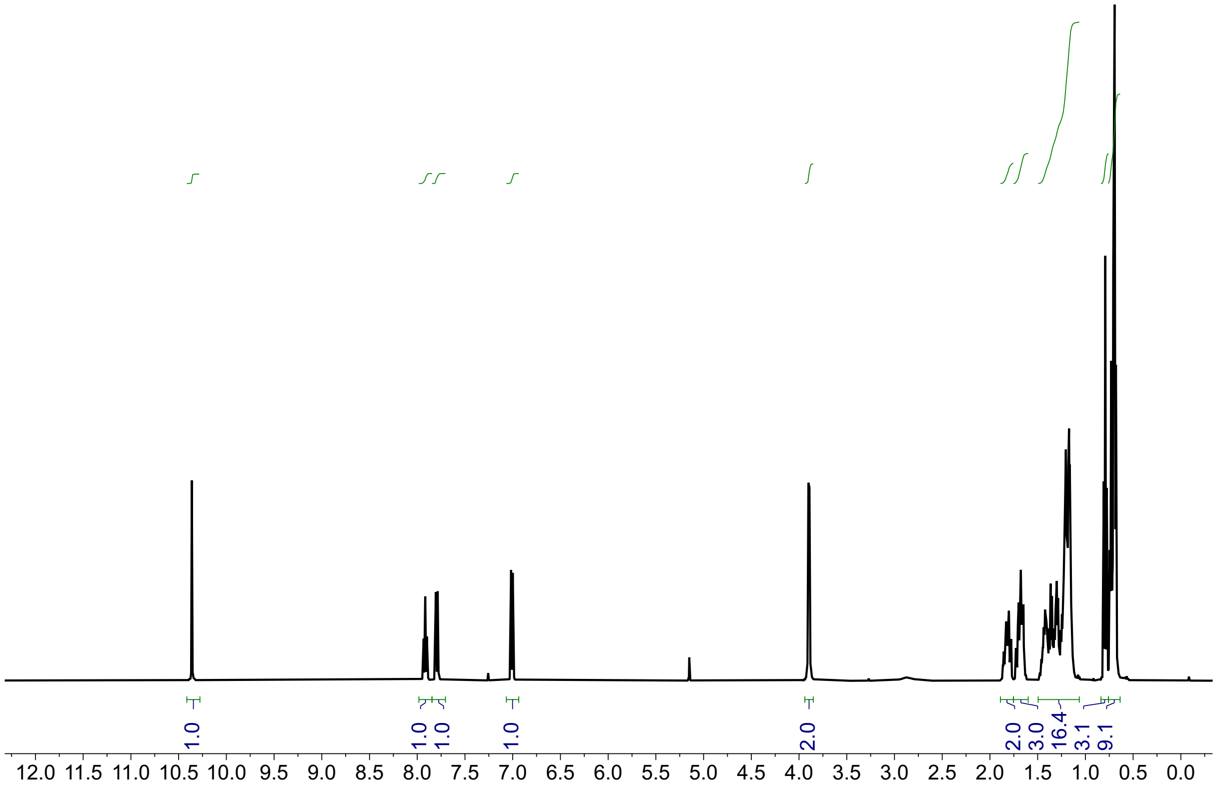

^{1}H-NMR spectrum

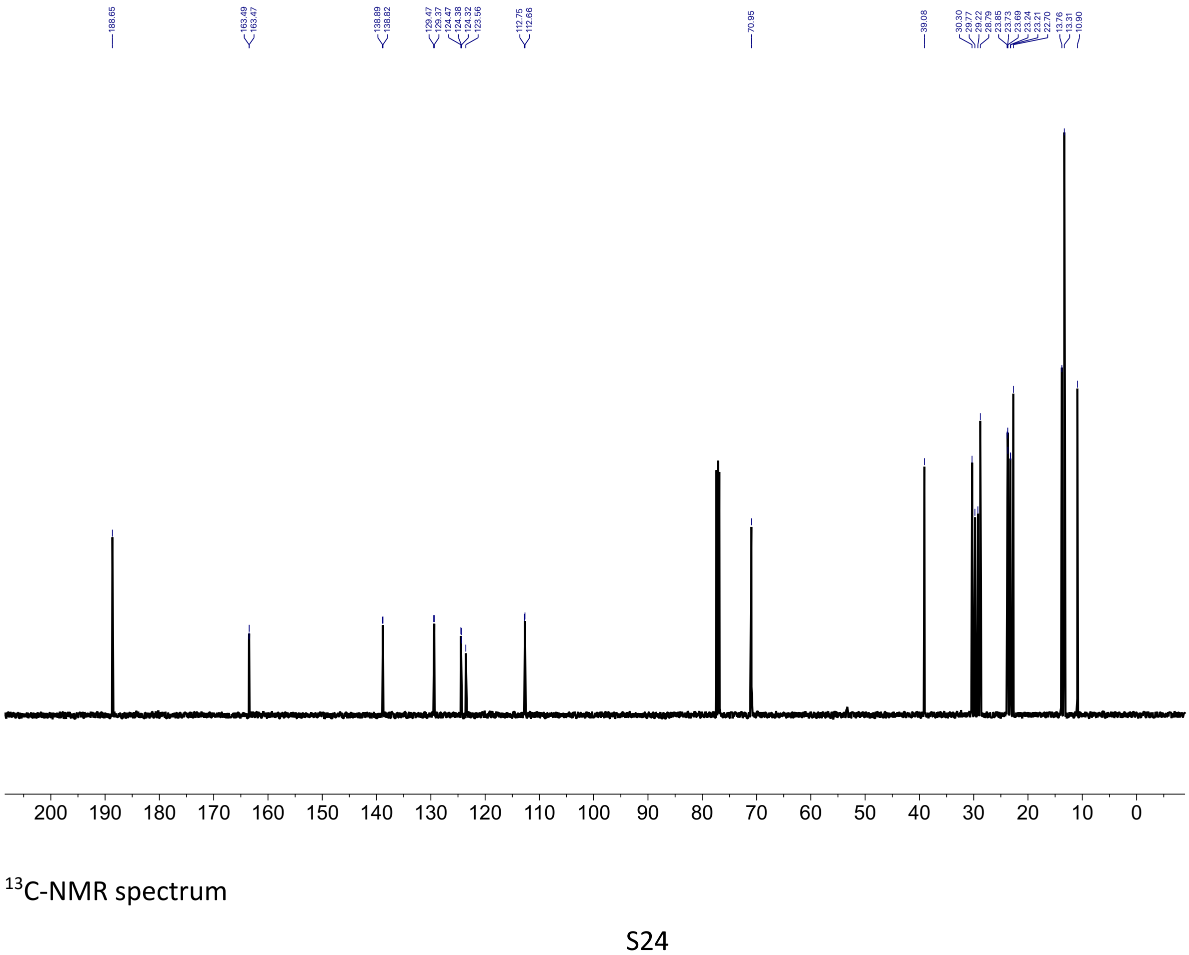

^{13}C-NMR spectrum

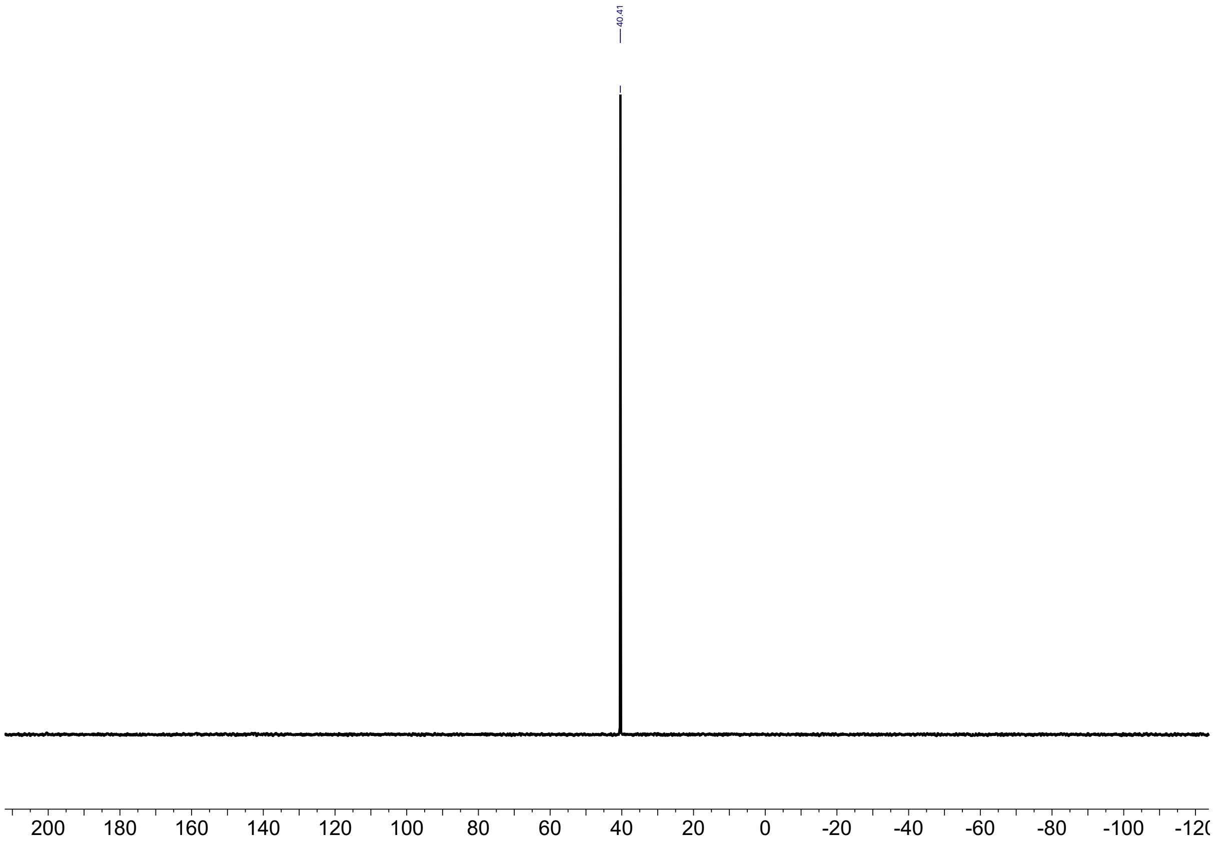

^{31}P-NMR spectrum

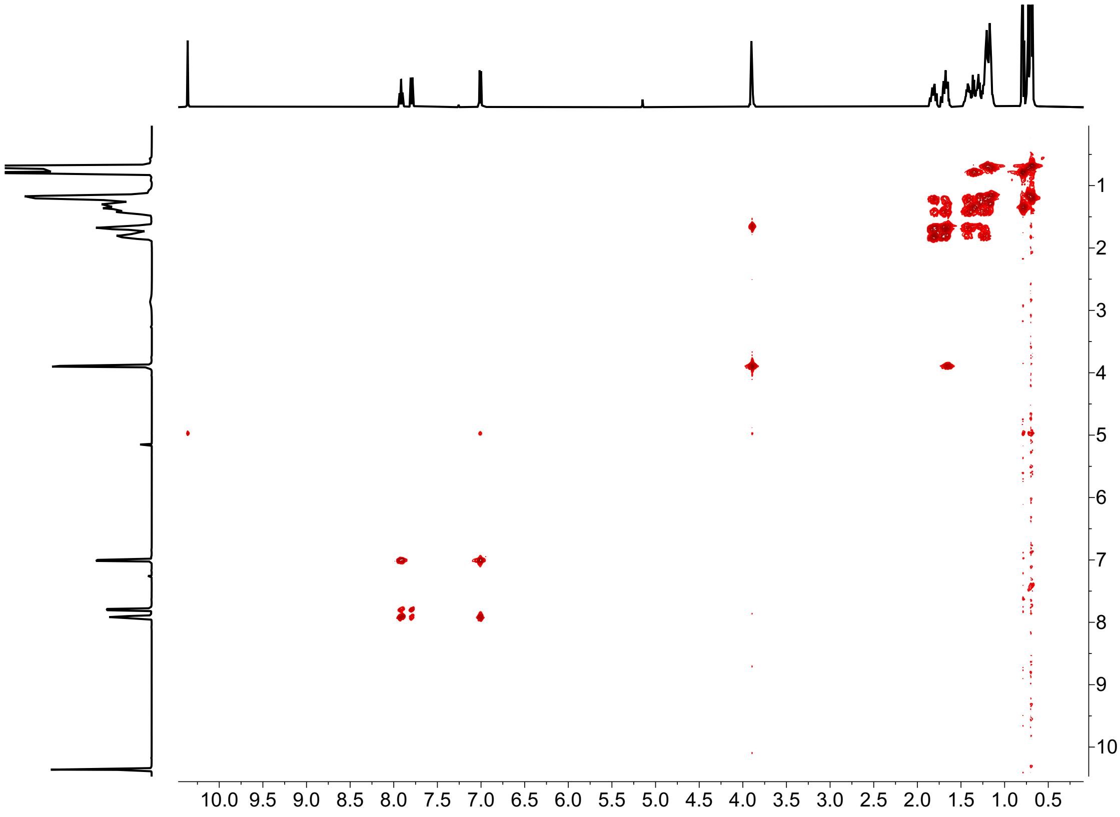

COSY-NMR spectrum

5. Synthesis of monomer A

Monomer **A'** was prepared as reported in the following scheme:

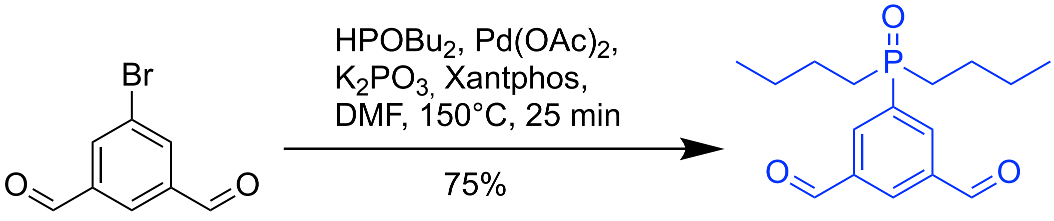

A

5.1 Synthesis of A.

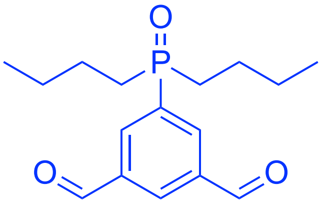

Acceptor monomer **A** was synthesized in one step, dissolving 5-bromoisophthalaldehyde (500 mg, 2.347 mmol, 1 equiv), phosphine oxide **5** (420 mg, 2.589 mmol, 1.1 equiv), Xantphos (163 mg, 0.282 mmol, 0.1 equiv), K_2PO_3 (550 mg, 2.589 mmol, 1.1 equiv) and $Pd(OAc)_2$ (53 mg, 0.235 mmol, 0.1 equiv) in freshly degassed DMF (4 mL, 3 freeze pump thaw cycles). Then the mixture was stirred under N_2 atmosphere, in a sealed vial, under MW irradiation (high absorption), at 150°C for 25 minutes (30 sec of pre-stirring). Then the mixture was extracted (3x) with EtOAc / LiCl 5%; the organic layers were dried with $MgSO_4$, concentrated under reduced pressure and then purified via combiflash chromatography (EtOAc:MeOH, from 0 to 10%), affording the desired monomer **A** (510 mg, 75% yield).

^{1}H NMR (500 MHz, Chloroform-*d*) δ 10.16 (s, 2H), 8.50 (s, 1H), 8.45 (dd, *J* = 9.9, 1.7 Hz, 2H), 2.12 – 2.00 (m, 2H), 1.96 – 1.84 (m, 2H), 1.67 – 1.57 (m, 2H), 1.39 – 1.30 (m, 6H), 0.85 (t, *J* = 7.2 Hz, 6H).
^{13}C NMR (126 MHz, Chloroform-*d*) δ 190.2, 137.04 (d, *J* = 9.6 Hz), 136.38 (d, *J* = 86.4 Hz), 136.22 (d, *J* = 9.1 Hz), 132.67 (d, *J* = 2.6 Hz), 29.50 (d, *J* = 68.8 Hz), 23.89 (d, *J* = 14.5 Hz), 23.33 (d, *J* = 4.4 Hz), 13.4.
^{31}P NMR (202 MHz, $CDCl_3$) δ 40.06.
HRMS (ASAP+): Calculated for $C_{16}H_{24}O_3P$ 295,1463 a.m.u., found 295,1450 a.m.u.

FT-IR (ATR): ν_{max} / cm^{-1} 3401, 2958, 2932, 2871, 2734, 2715, 1702, 1590, 1542, 1466, 1405, 1378, 1345, 1313, 1258, 1221, 1169, 1141, 1122, 1095, 1053, 987, 964, 907, 882, 860, 798, 683, 650.

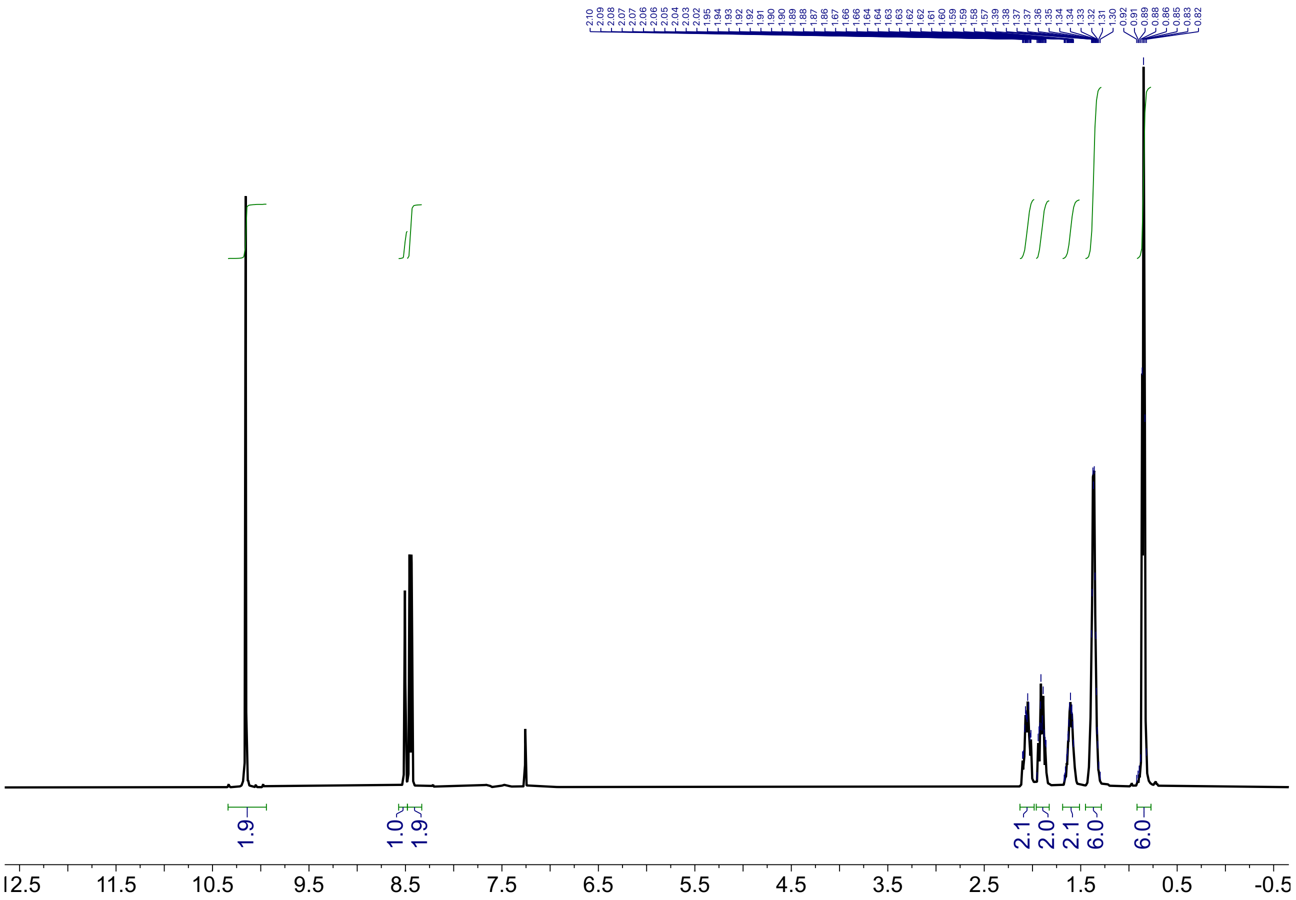

^{1}H-NMR spectrum

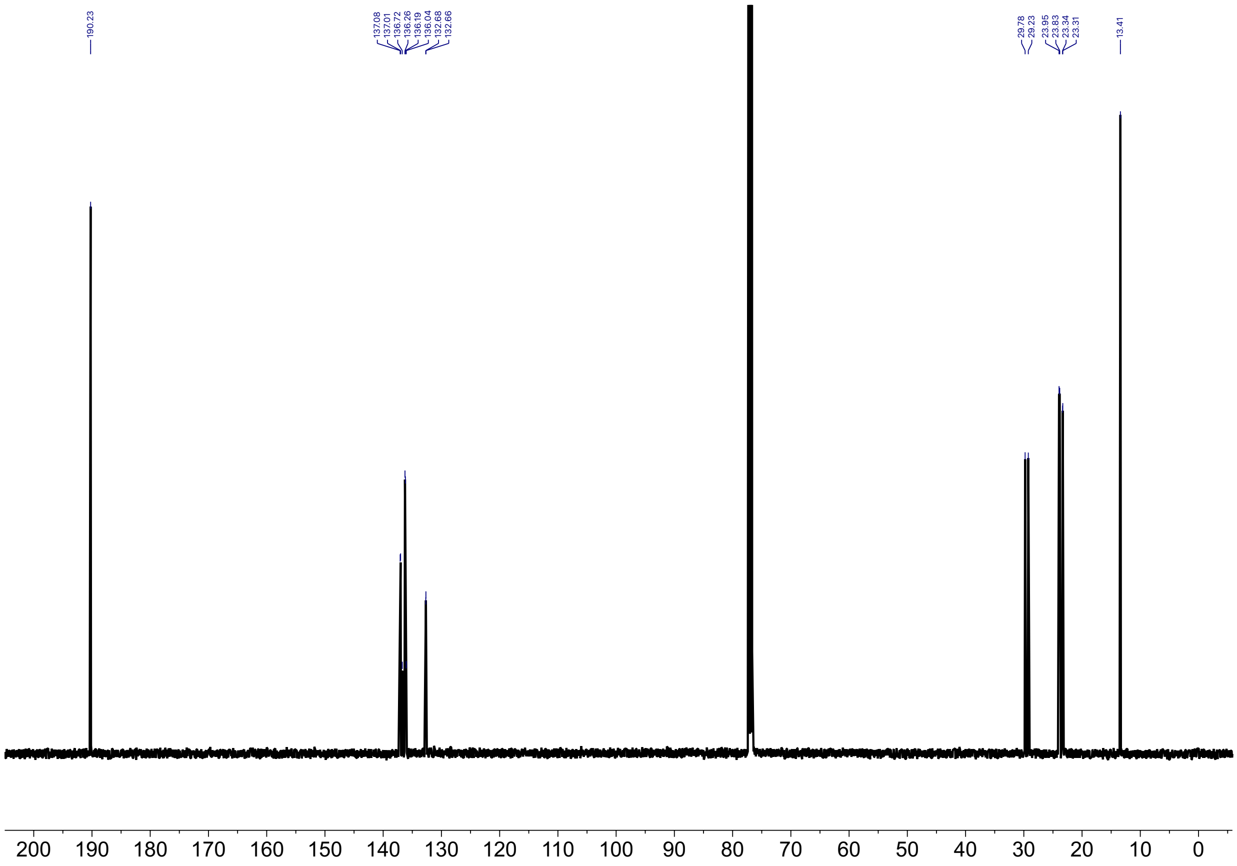

^{13}C-NMR spectrum

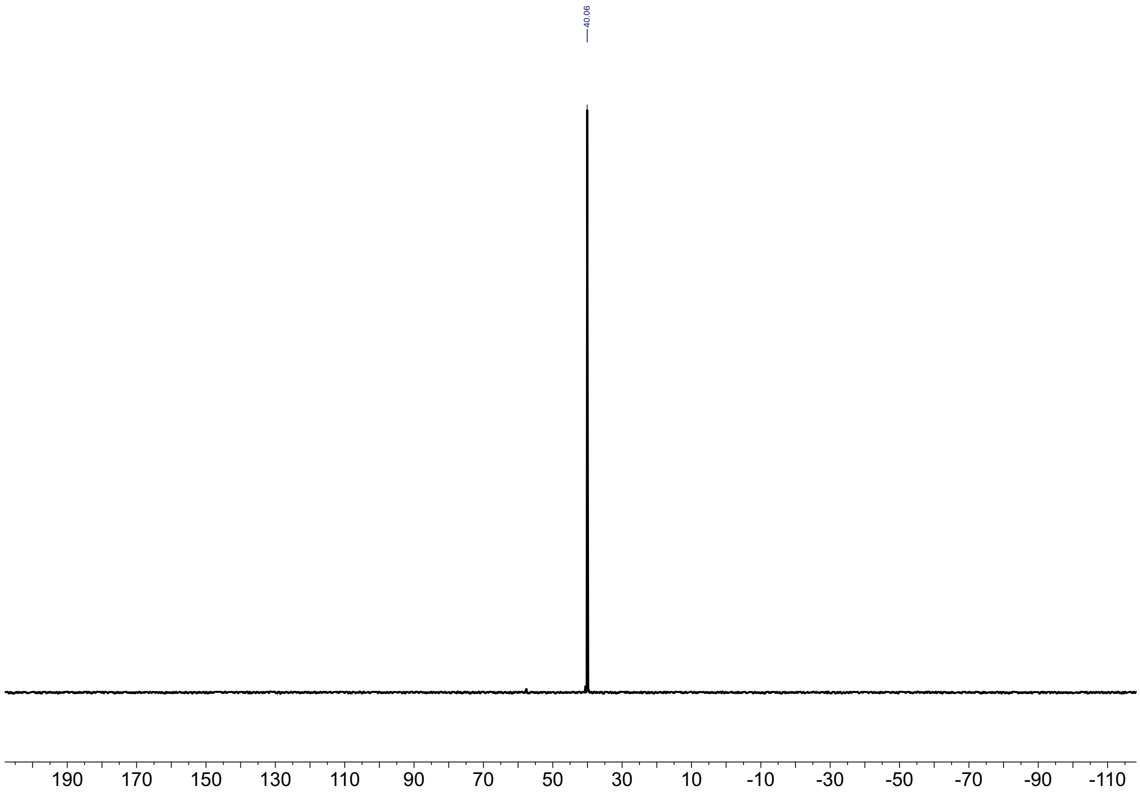

^{31}P-NMR spectrum

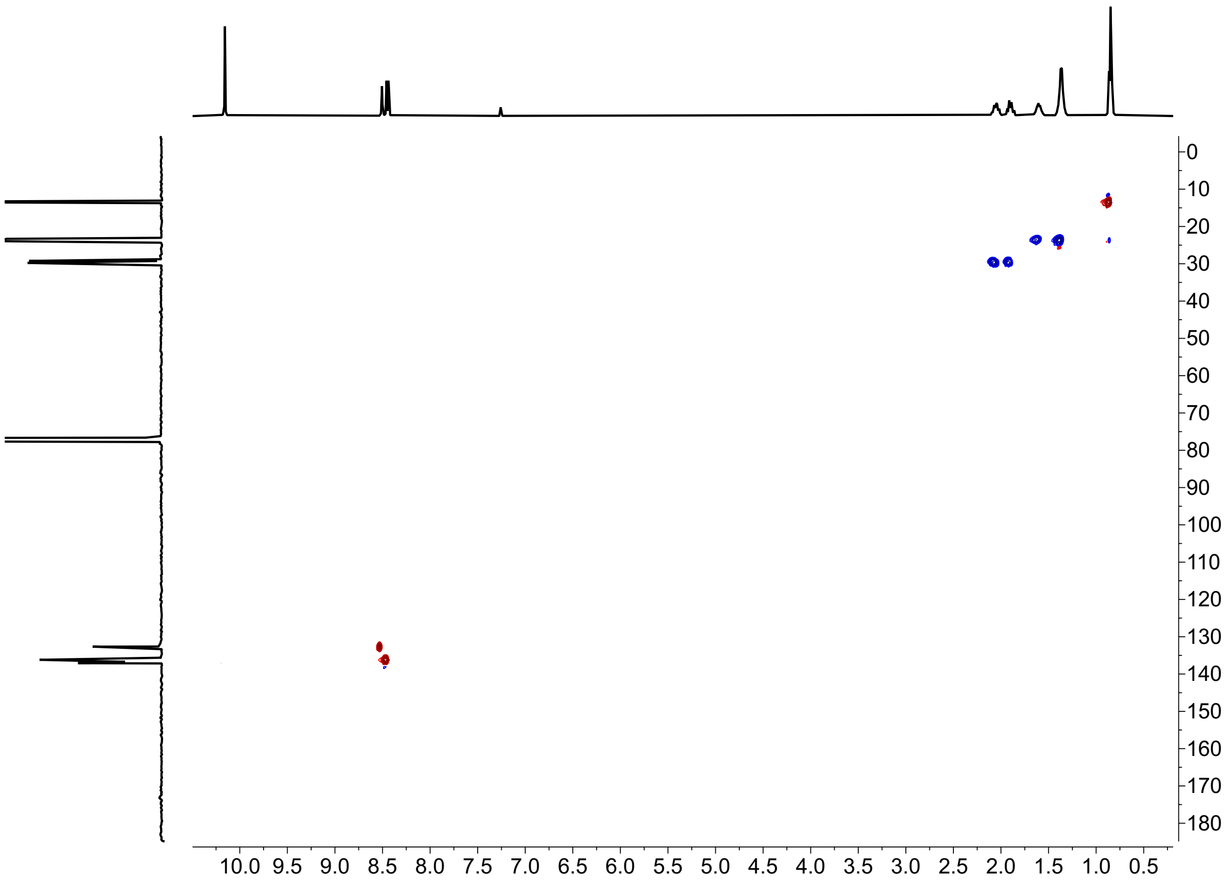

HSQC-NMR spectrum

6. Synthesis of dimer DD

Monomer **DD** was prepared as reported in the following scheme:

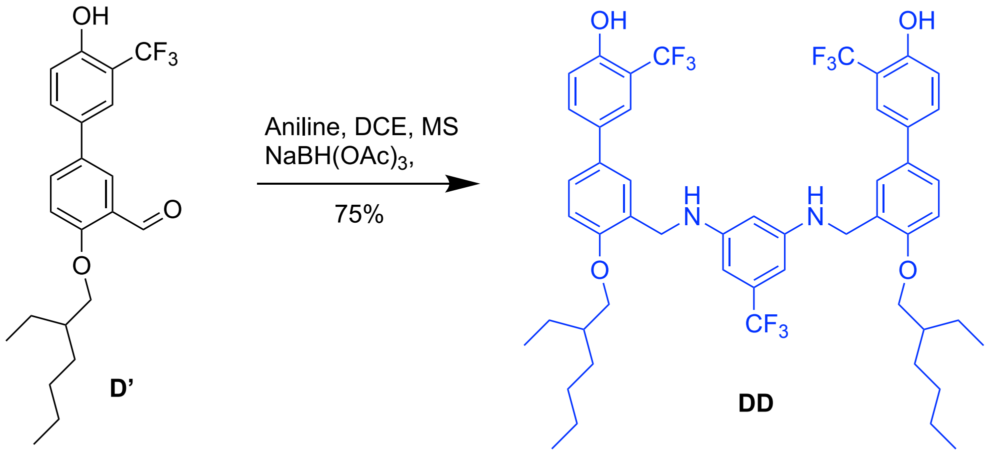

6.1 Synthesis of DD.

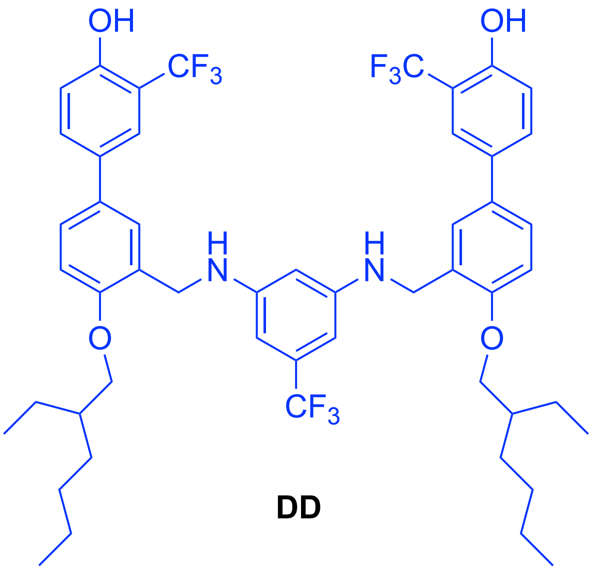

Donor dimer **DD** was synthesized *via* one step reductive amination. Monomer **D'** (100 mg, 0.237 mmol, 1 equiv) and 5-(trifluoromethyl)benzene-1,3-diamine (20 mg, 0.237 mmol, 0.5 equiv) were dissolved in dry dichloroethane (2 mL). Then molecular sieves (3 A) are added and the reaction is shaken on an orbital oscillator for 6 hours, under N_2 atmosphere. Subsequently, $NaBH(OAc)_3$ (50 mg, 0.237 mmol, 1 equiv) was added and the mixture was shaken for 12 hours. Then, in order to be sure that the reduction is fully complete (since imine impurities co-eluate with desired dimer) $NaBH_4$

(9 mg, 0.237 mmol, 1 equiv) and MeOH (0.5 mL) are added and the mixture is shaken for one hour. Then the solution is filtered, MS are washed with DCM and a saturated solution of $NaHCO_3$ is added. The mixture is stirred until complete solubilisation, then the organic phase is extracted and the aqueous phase is washed (3x) with EtOAc. The organic layers are collected and dried over $MgSO_4$, the solvent is evaporated and the residue is purified via combiflash chromatography (PE:EtOAc), giving the desired dimer **DD** (163 mg, 75% yield).

^{1}H NMR (500 MHz, $CDCl_3$) δ 7.60 (d, *J* = 2.3 Hz, 2H), 7.49 (dd, *J* = 8.5, 2.3 Hz, 2H), 7.42 (d, *J* = 2.4 Hz, 2H), 7.36 (dd, *J* = 8.4, 2.4 Hz, 2H), 6.92 (dd, *J* = 8.5, 1.7 Hz, 4H), 6.30 (d, *J* = 1.9 Hz, 2H), 6.05 (s, 2H), 4.34 (s, 4H), 3.93 (dd, *J* = 5.5, 1.8 Hz, 4H), 1.80 – 1.72 (m, 2H), 1.69 (s, 2H), 1.53 – 1.36 (m, 8H), 1.36 – 1.22 (m, 9H), 0.92 (t, *J* = 7.5 Hz, 6H), 0.91 – 0.84 (m, 6H).
^{13}C NMR (126 MHz, $CDCl_3$) δ 156.56, 152.27, 149.47, 133.58, 132.15 (dd, *J* = 62.6, 31.2 Hz),131.49, 127.37, 126.58, 124.36 (d, *J* = 272.6 Hz), 124.00 (d, *J* = 272.3 Hz), 118.00, 116.53 (q, *J* = 30.3 Hz), 111.36, 99.87, 99.69 (dd, *J* = 7.6, 3.6 Hz), 70.33, 43.75, 39.31, 30.62, 29.00, 23.96, 22.90, 13.93, 11.04.
^{19}F NMR (471 MHz, $CDCl_3$) δ -61.33, -63.38.
HRMS (ES+): Calculated for $C_{51}H_{58}N_2O_4F_9$ 933.4253 a.m.u., found 933.4263 a.m.u.
FT-IR (ATR): v_{max} / cm^{-1} 3448, 2959, 2928, 2874, 2862, 1612, 1494, 1463, 1440, 1381, 1335, 1284, 1247, 1165, 1053, 899, 891812, 695.

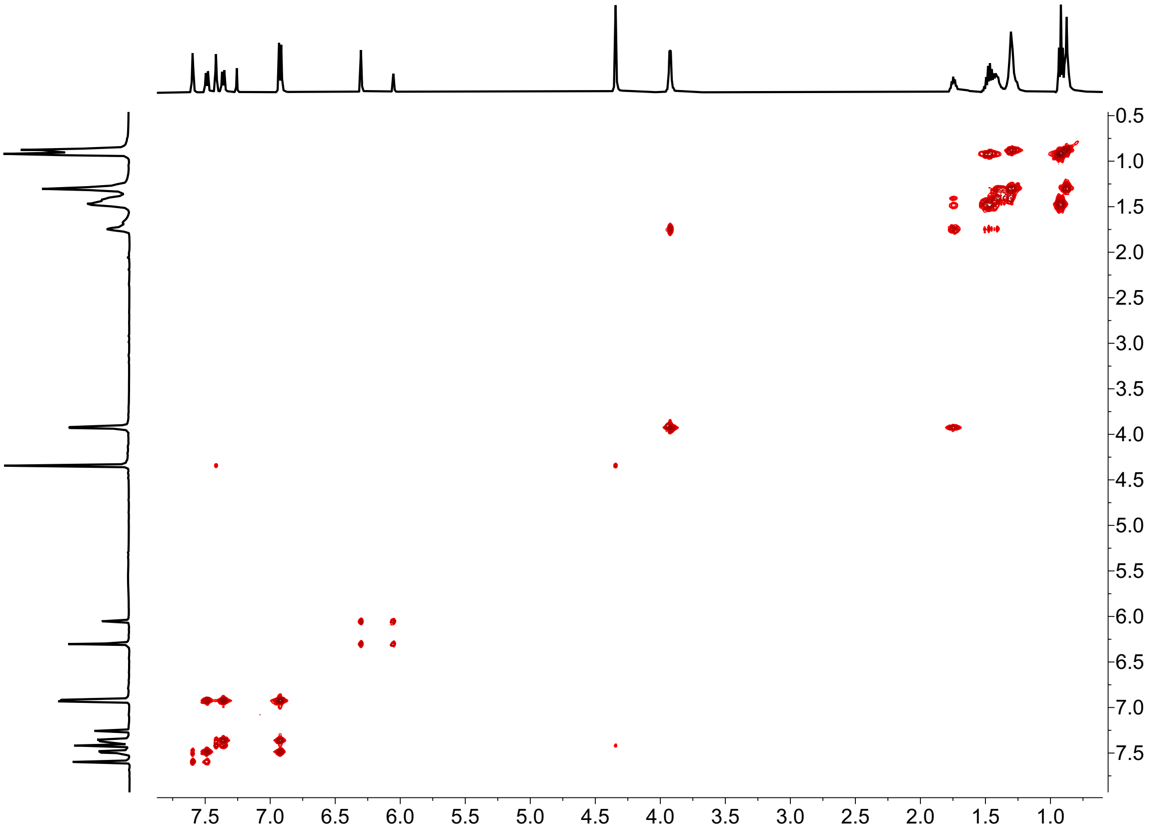

COSY-NMR spectrum

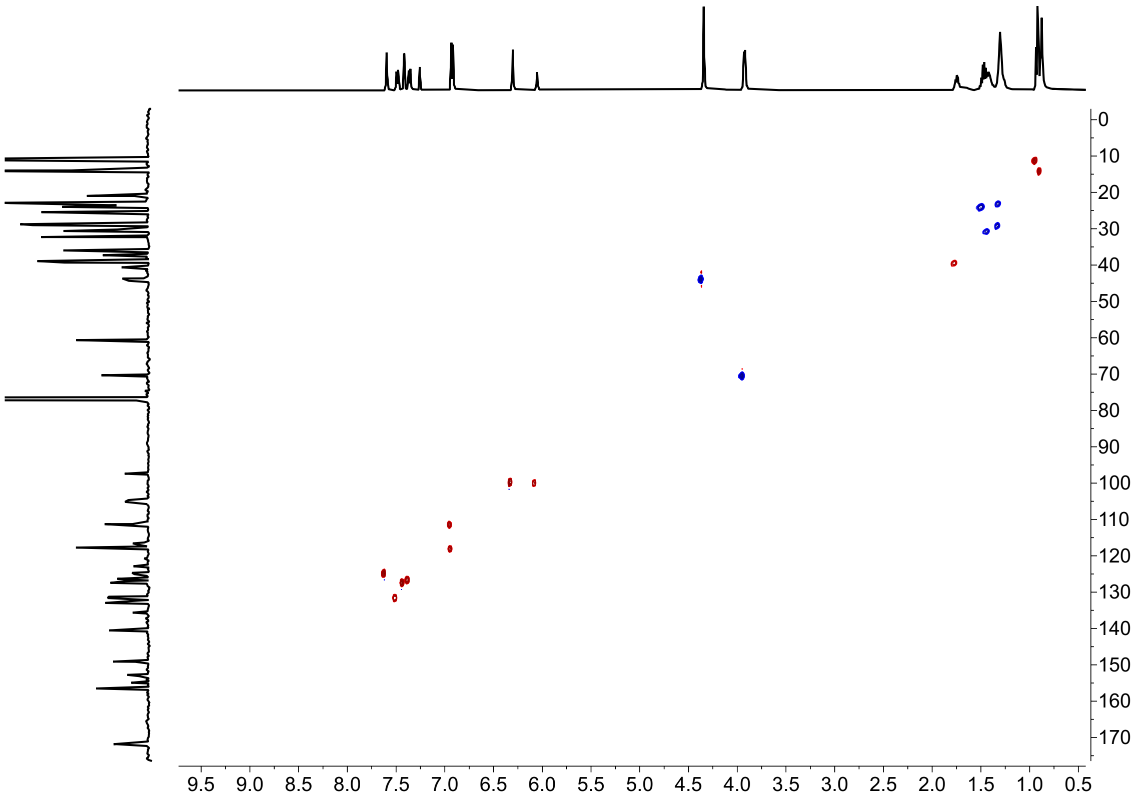

HSQC-NMR spectrum

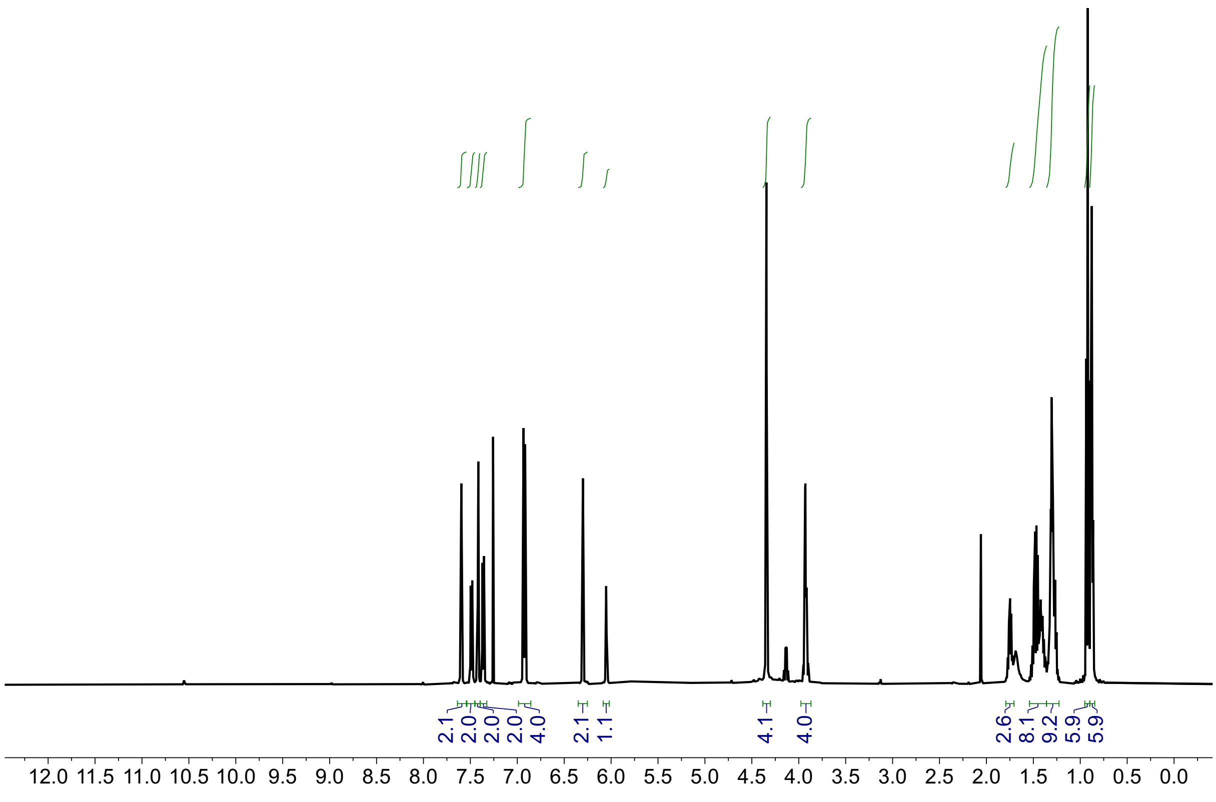

^{1}H-NMR spectrum

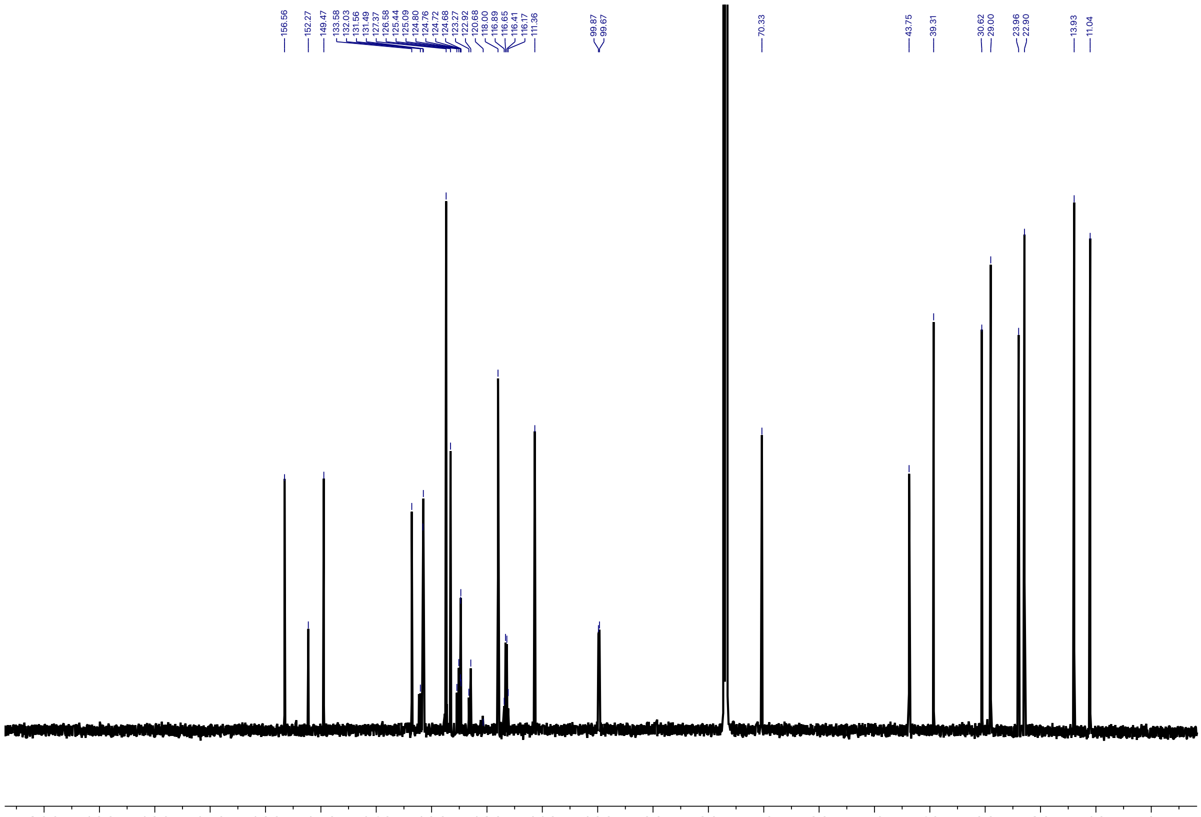

^{13}C-NMR spectrum

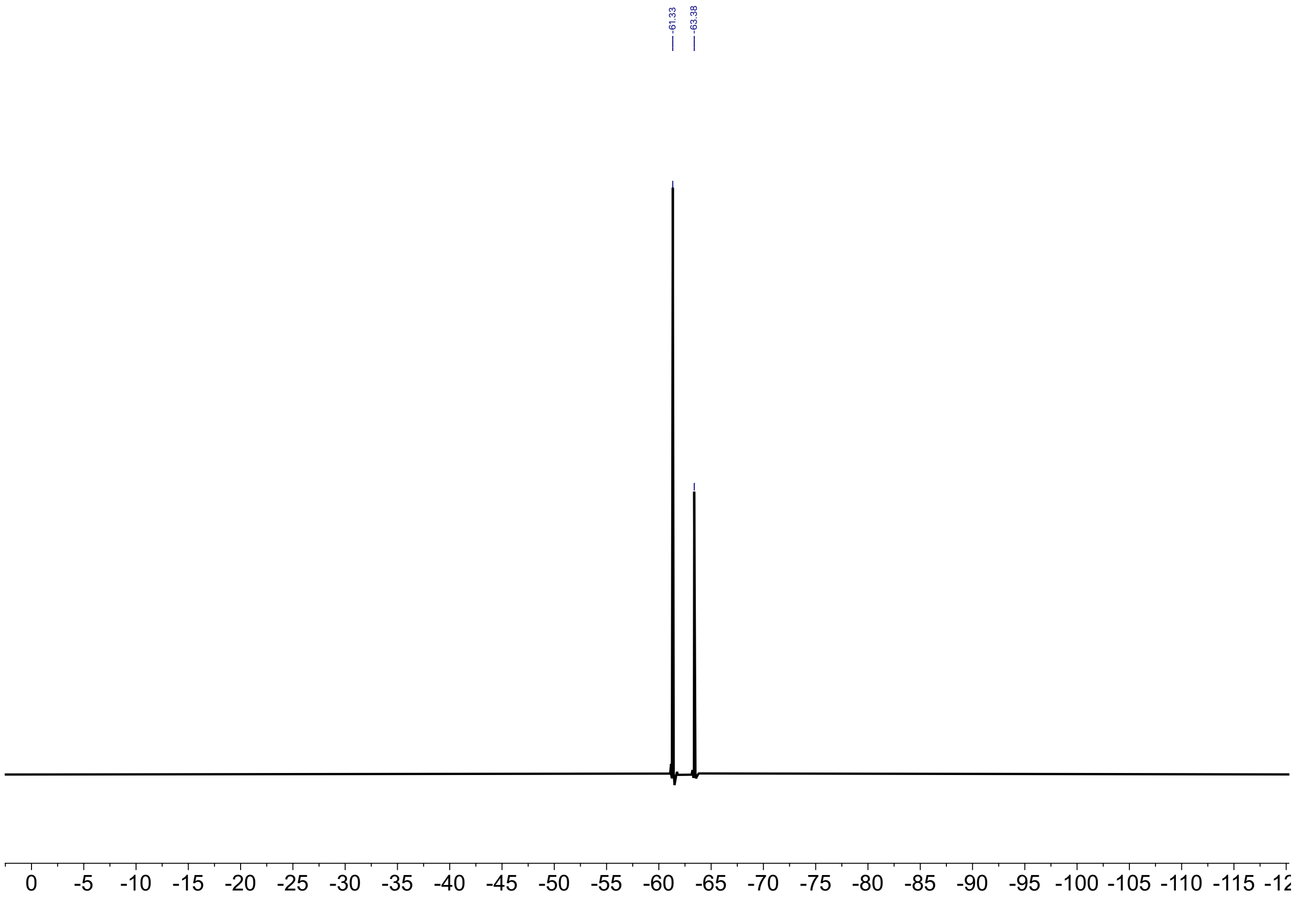

^{19}F-NMR spectrum

7. Synthesis of dimer AA

Monomer **AA** was prepared as reported in the following scheme:

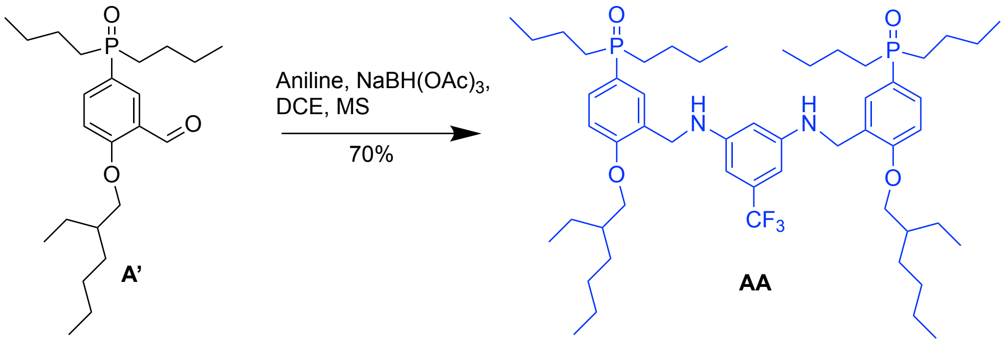

7.1 Synthesis of AA.

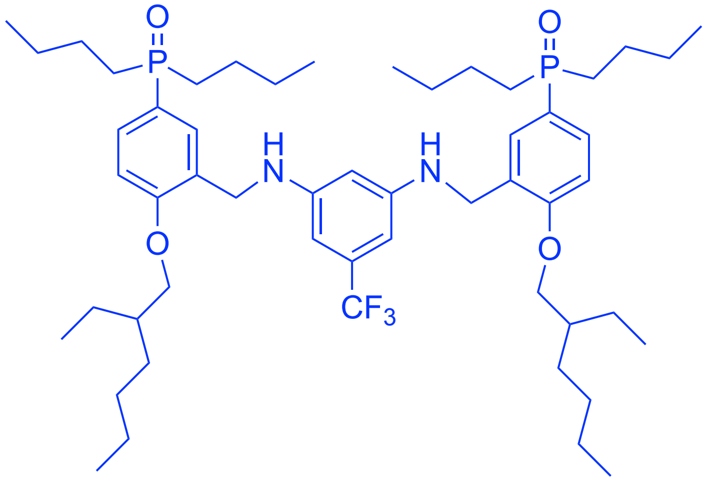

Acceptor dimer **AA** was synthesized *via* one step reductive amination. Monomer **A'** (108 mg, 0.275 mmol, 1 equiv) and 5-(trifluoromethyl)benzene-1,3-diamine (24 mg, 0.275 mmol, 0.5 equiv) were dissolved in dry dichloroethane (2 mL). Then molecular sieves (3 A) are added and the reaction is shaken on an orbital oscillator for 6 hours, under N_2 atmosphere. After imine formation $NaBH(OAc)_3$ (58 mg, 0.275 mmol, 1 equiv) was added and the mixture was shaken for 12 hours. Then, in order to be sure that the reduction is fully complete (since imine impurities co-eluate with desired dimer) $NaBH_4$ (10 mg, 0.275 mmol, 1 equiv) and MeOH (0.5 mL) are added and the mixture is shaken for one hour. Then the solution is filtered, MS are washed with DCM and a saturated solution of $NaHCO_3$

is added. The mixture is stirred until complete solubilisation, then the organic phase is extracted and the aqueous phase is washed (3x) with EtOAc. The organic layers are collected and dried over $MgSO_4$, the solvent is evaporated and the residue is purified via combiflash chromatography (EtOAc:MeOH), giving the desired dimer **AA** (179 mg, 70% yield).

^{1}H NMR (500 MHz, Chloroform-*d*) δ 7.65 – 7.54 (m, 2H), 7.49 (d, *J* = 10.3 Hz, 2H), 6.96 (d, *J* = 8.6 Hz, 2H), 6.17 (s, 2H), 6.00 (s, 1H), 4.30 (s, 7H), 3.99 – 3.85 (m, 5H), 2.38 (s, 4H), 1.87 (ddq, *J* = 15.5, 11.9, 4.9 Hz, 5H), 1.74 (qd, *J* = 14.5, 12.7, 4.1 Hz, 7H), 1.57 – 1.22 (m, 33H), 0.91 (t, *J* = 7.5 Hz, 7H), 0.89 – 0.84 (m, 5H), 0.81 (t, *J* = 7.1 Hz, 14H).
^{13}C NMR (126 MHz, $CDCl_3$) δ 159.44, 159.42, 149.21, 131.57 (d, *J* = 9.2 Hz), 130.29 (d, *J* = 10.9 Hz), 127.53 (d, *J* = 186.5 Hz), 127.34 (d, *J* = 11.4 Hz), 124.29 (q, *J* = 272.5 Hz), 122.91 (d, *J* = 97.3 Hz), 110.95, 110.85, 100.01, 99.26, 99.24, 99.20, 99.17, 70.36, 43.29, 39.24, 30.57, 29.90, 29.36, 28.97, 23.36 (d, *J* = 4.0 Hz), 22.87, 13.91, 13.41, 11.04.
^{31}P NMR (202 MHz, $CDCl_3$) δ 41.17.
^{19}F NMR (471 MHz, $CDCl_3$) δ -63.44.
HRMS (ES+): Calculated for $C_{53}H_{86}N_2O_4F_3P_2$ 933,6015 a.m.u., found 933,5975 a.m.u.
FT-IR (ATR): ν_{max} / cm^{-1} 3280, 2959, 2931, 2872, 1598, 1552, 1510, 1492, 1466, 1394, 1380, 1347, 1324, 1275, 1246, 1223, 1187, 1159, 1116, 1084, 1029, 909, 815, 762, 713, 698.

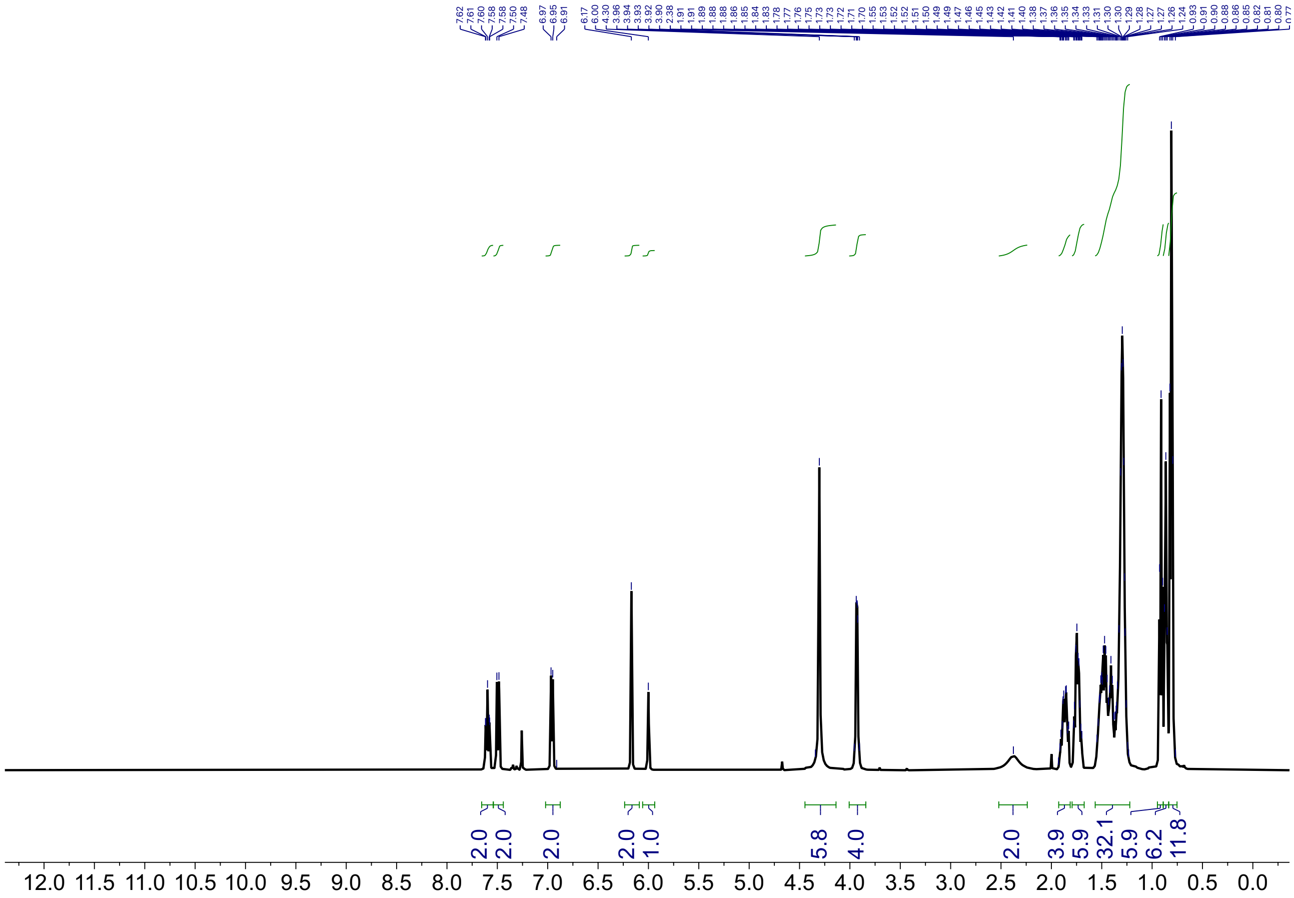

^{1}H-NMR spectrum

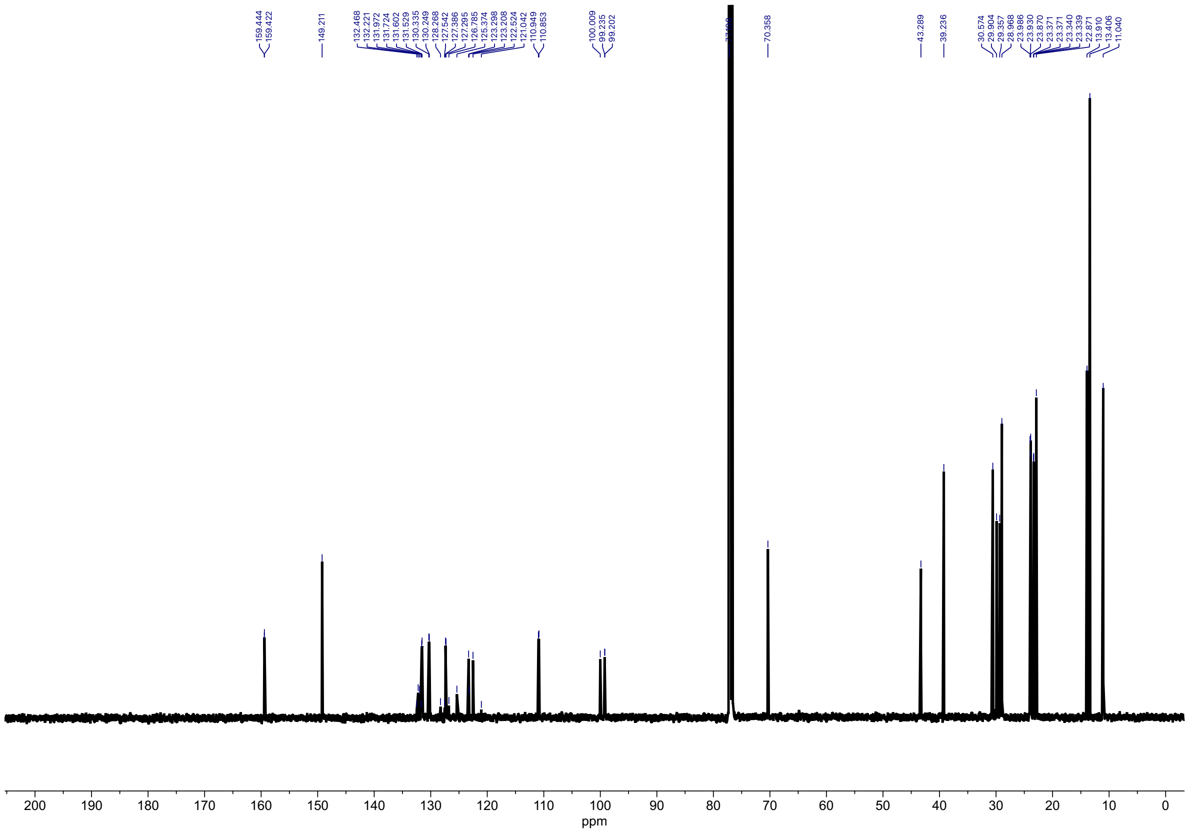

^{13}C-NMR spectrum

^{31}P-NMR spectrum

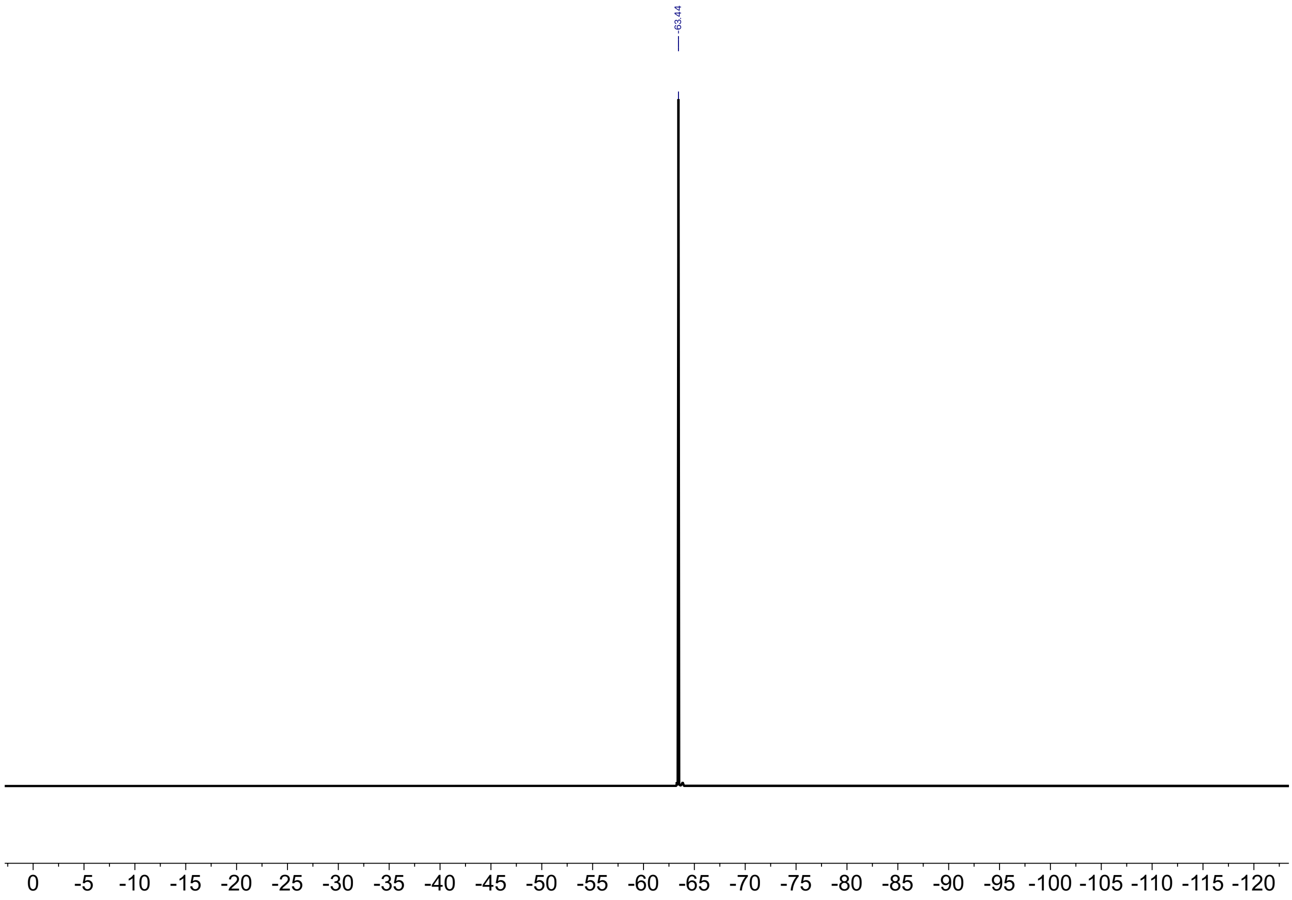

^{19}F-NMR spectrum

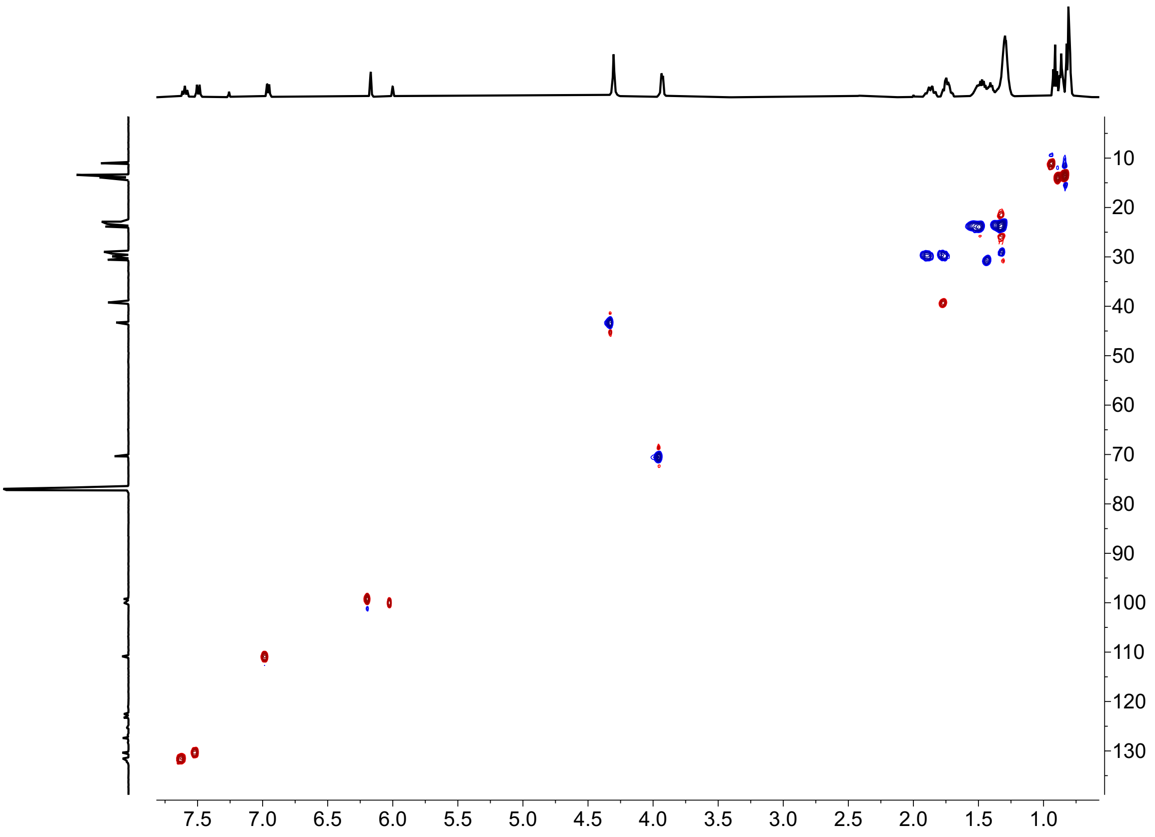

HSQC-NMR spectrum

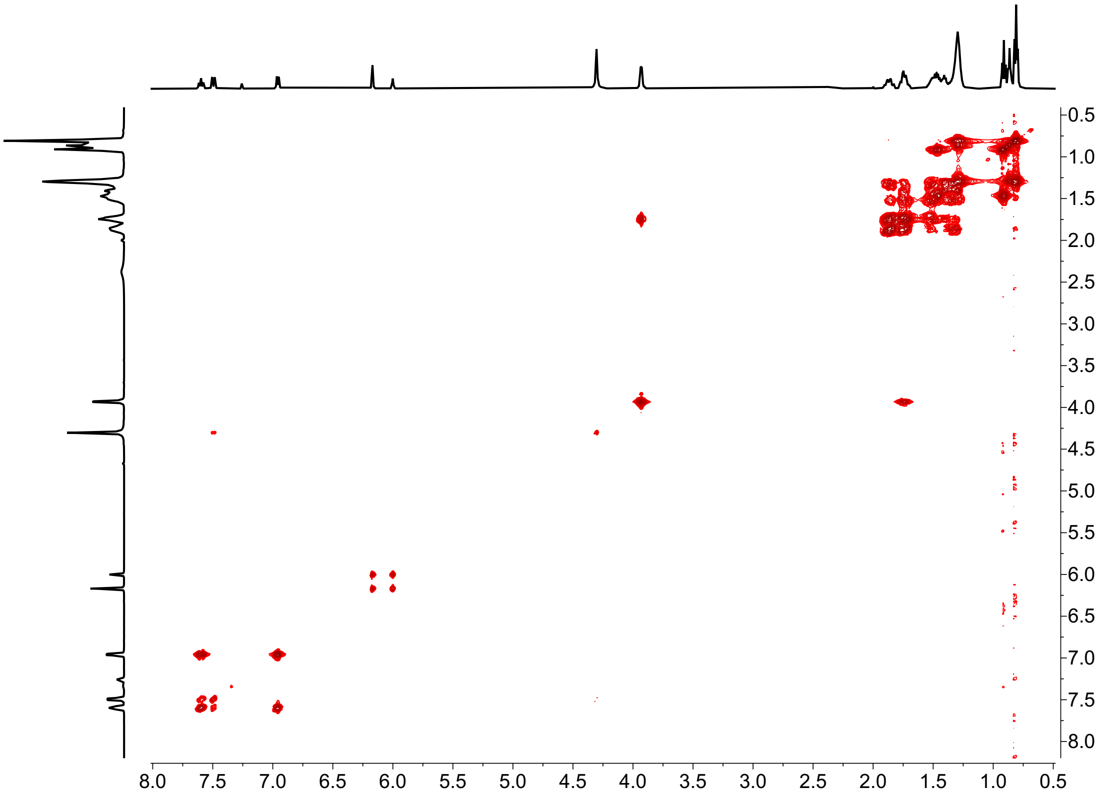

COSY-NMR spectrum

8. Synthesis of aniline 7

Aniline **7** was prepared as reported in the following scheme:

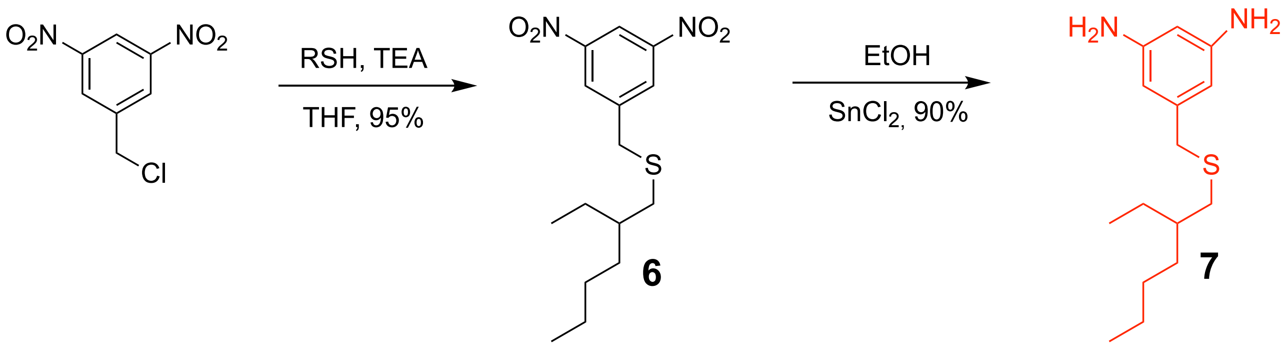

8.1 Synthesis of 6.

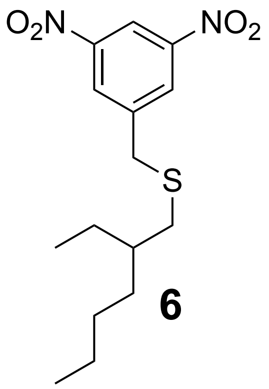

1-(chloromethyl)-3,5-dinitrobenzene (3 g, 13.851 mmol, 1 equiv) was dissolved in dry THF (45 mL) and trimethylamine (2.3 mL, 16.621, 1.2 equiv). Then, 2-ethylhexane-1-thiol was added, and the reaction was stirred under N2 atmosphere. A pink precipitate was formed, and after 15 minutes the solvent was evaporated and the residue was firstly extracted (3x) with DCM / NaOH 2N. The organic layers were collected, dried over $MgSO_4$ and purified via combiflash chromatography (PE:EtOAc), giving 4.3 g of compound **6** (95% yield).

^{1}H NMR (500 MHz, $CDCl_3$-*d*) δ 8.94 (t, *J* = 2.1 Hz, 1H), 8.56 (d, *J* = 2.2 Hz, 2H), 3.88 (s, 2H), 2.45 (d, *J* = 6.2 Hz, 2H), 1.51 – 1.14 (m, 9H), 0.88 (t, *J* = 7.2 Hz, 3H), 0.84 (t, *J* = 7.4 Hz, 3H).

^{13}C NMR (126 MHz, $CDCl_3$) δ 149.11, 143.97, 128.91, 117.47, 38.95, 36.45, 36.09, 32.33, 28.85, 25.54, 22.91, 14.04, 10.77.

HRMS (ES+): Calculated for C_{15} H_{23} N_2 O_4 S 327,1379 a.m.u., found 327,1371 a.m.u.

FT-IR (ATR): ν_{max} / cm^{-1} 3107, 3084, 2958, 2927, 2872, 2858, 1594, 1538, 1459, 1358, 1341, 1235, 1115, 1075, 909, 809, 729, 661.

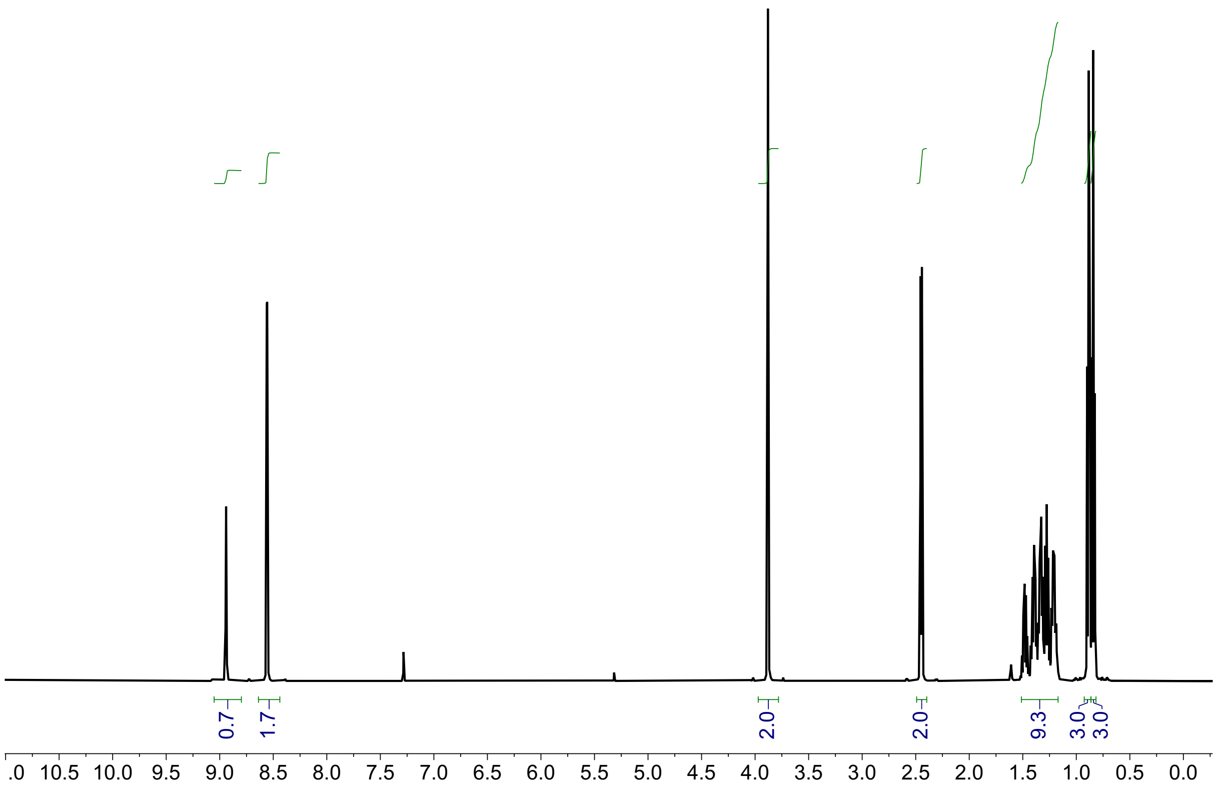

^{1}H-NMR spectrum

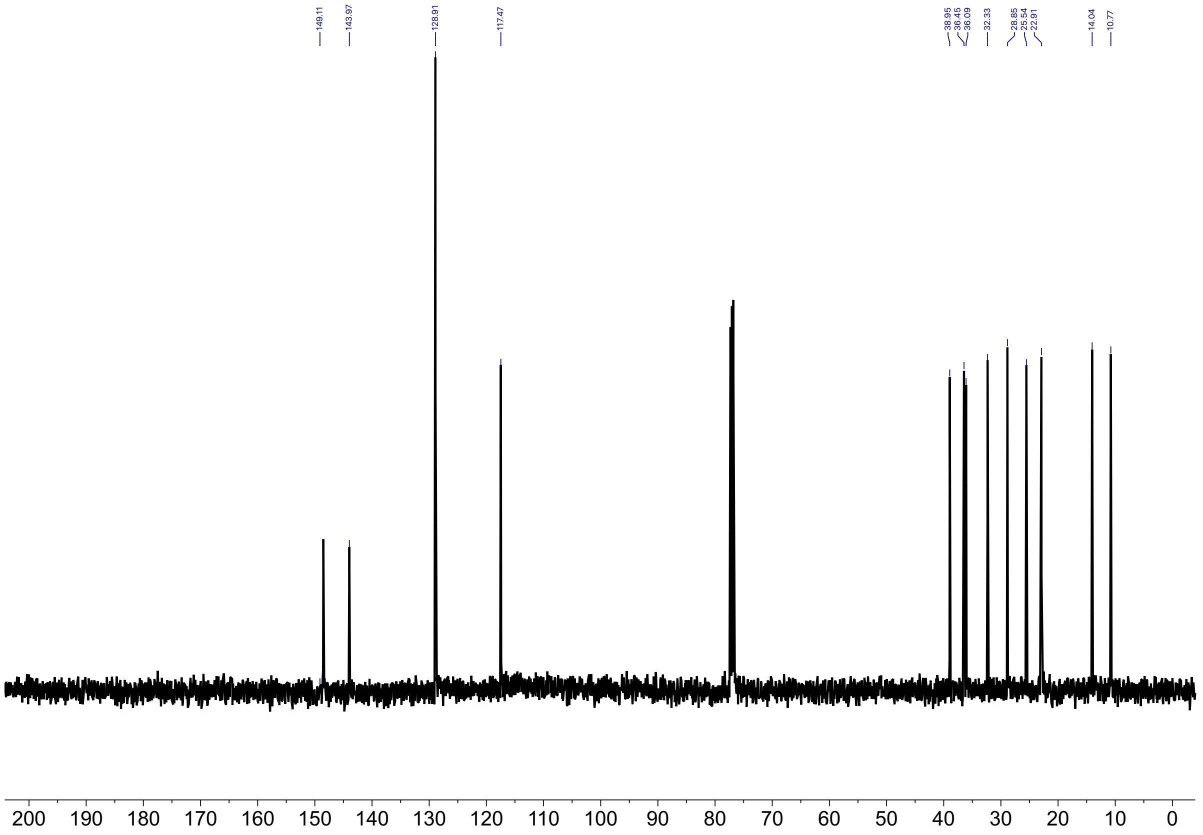

^{13}C-NMR spectrum

8.2 Synthesis of 6.

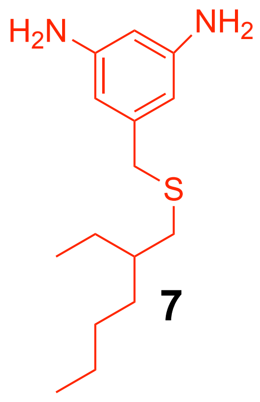

7

Compound **6** (4.3 g, 13.185 mmol, 1 equiv) was dissolved in EtOH 96% (75 mL) then $SnCl_2$ (20 g, 105.48 mmol, 8 equiv) was added, and the reaction was refluxed for 10 minutes. Then, solvent was evaporated the residue was firstly extracted (3x) with DCM / NaOH 2N. The organic layers were collected, dried over $MgSO_4$ and purified via combiflash chromatography (PE:EtOAc), giving 3.6 g of compound **7** (90% yield).

^{1}H NMR (500 MHz, $CDCl_3$-*d*) δ 6.11 (d, *J* = 2.1 Hz, 2H), 5.92 (t, *J* = 2.1 Hz, 1H), 3.53 (s, 4H), 3.51 (s, 2H), 2.44 (d, *J* = 6.2 Hz, 2H), 1.51 – 1.19 (m, 9H), 0.91 (t, *J* = 7.2 Hz, 3H), 0.86 (t, *J* = 7.4 Hz, 3H).
^{13}C NMR (126 MHz, $CDCl_3$) δ 147.52, 140.33, 106.64, 100.25, 39.00, 37.00, 36.09, 32.46, 28.91, 25.61, 23.01, 14.16, 10.85.
HRMS (ES+): Calculated for C_{15} H_{27} N_2 S 267, 1884 a.m.u., found 267,1895 a.m.u.
FT-IR (ATR): ν_{max} / cm^{-1} 3424, 3342, 3216, 3024, 2956, 2924, 2871, 2856, 1596, 1514, 1466, 1378, 1357, 1295, 1232, 1191, 1158, 1004, 992, 834, 725, 686, 630.

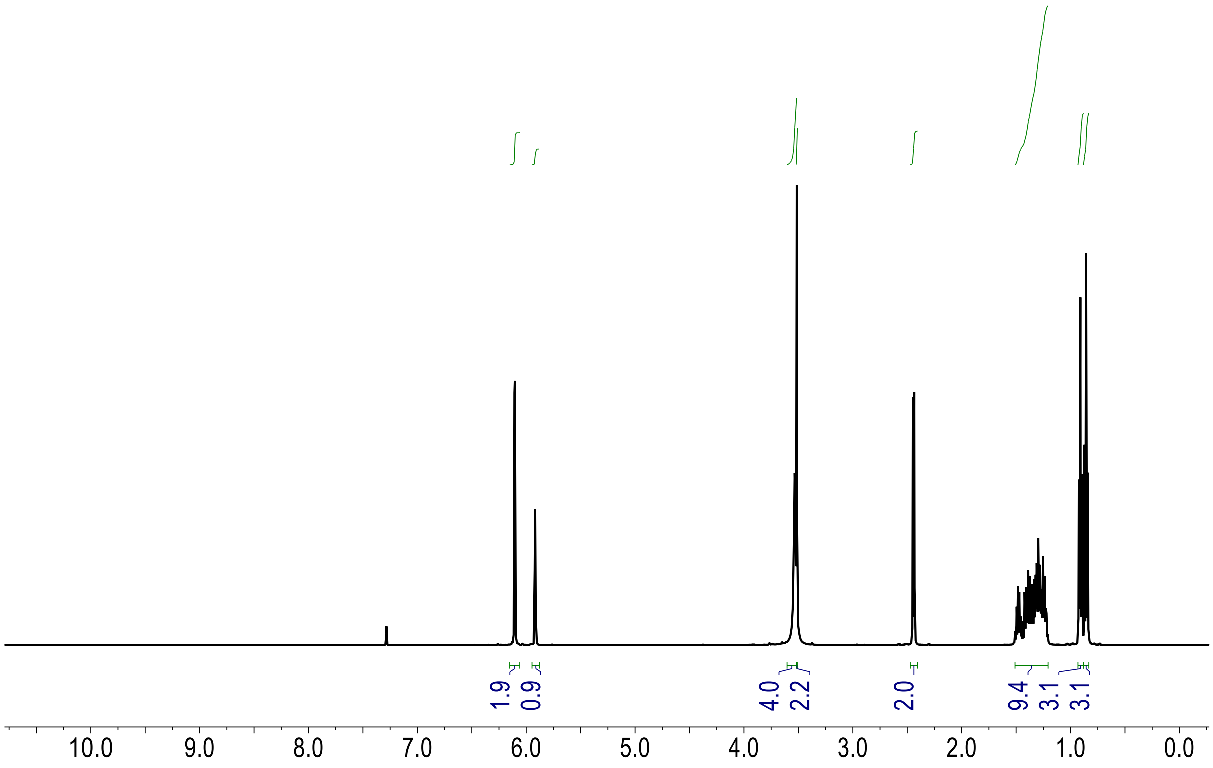

^{1}H-NMR spectrum

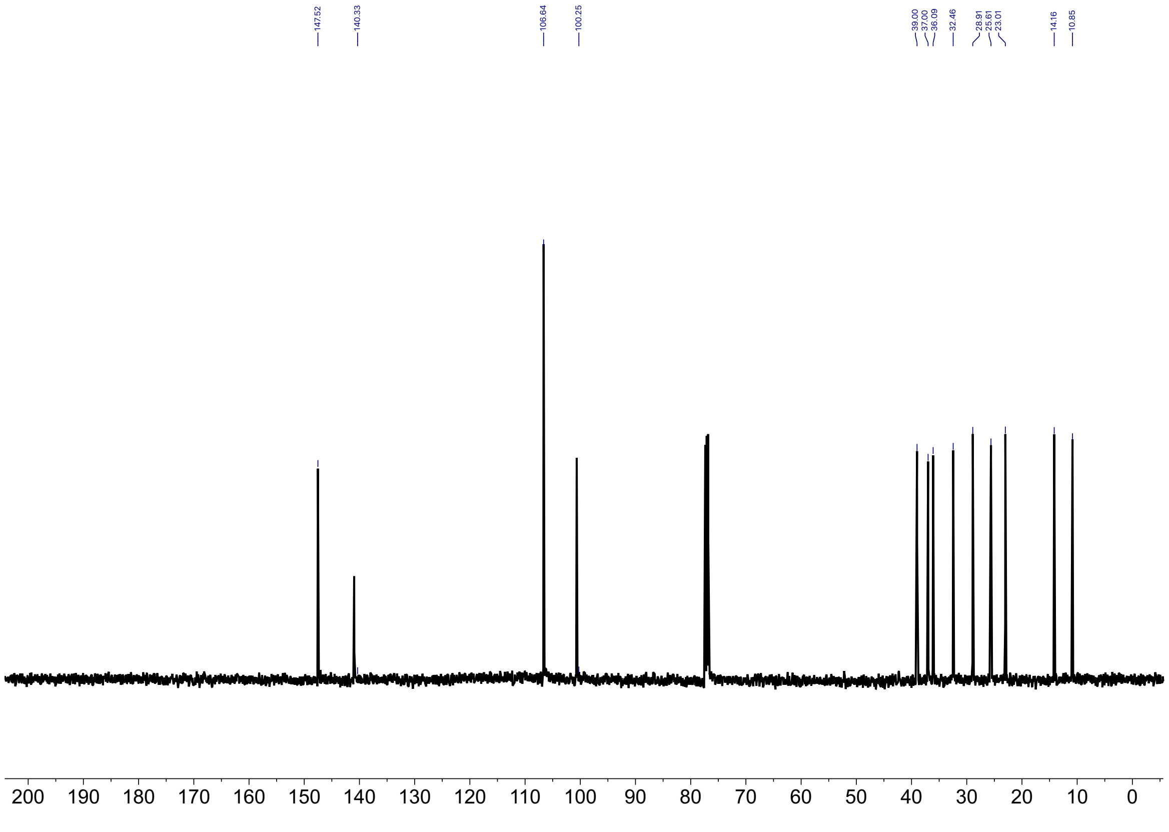

^{13}C-NMR spectrum

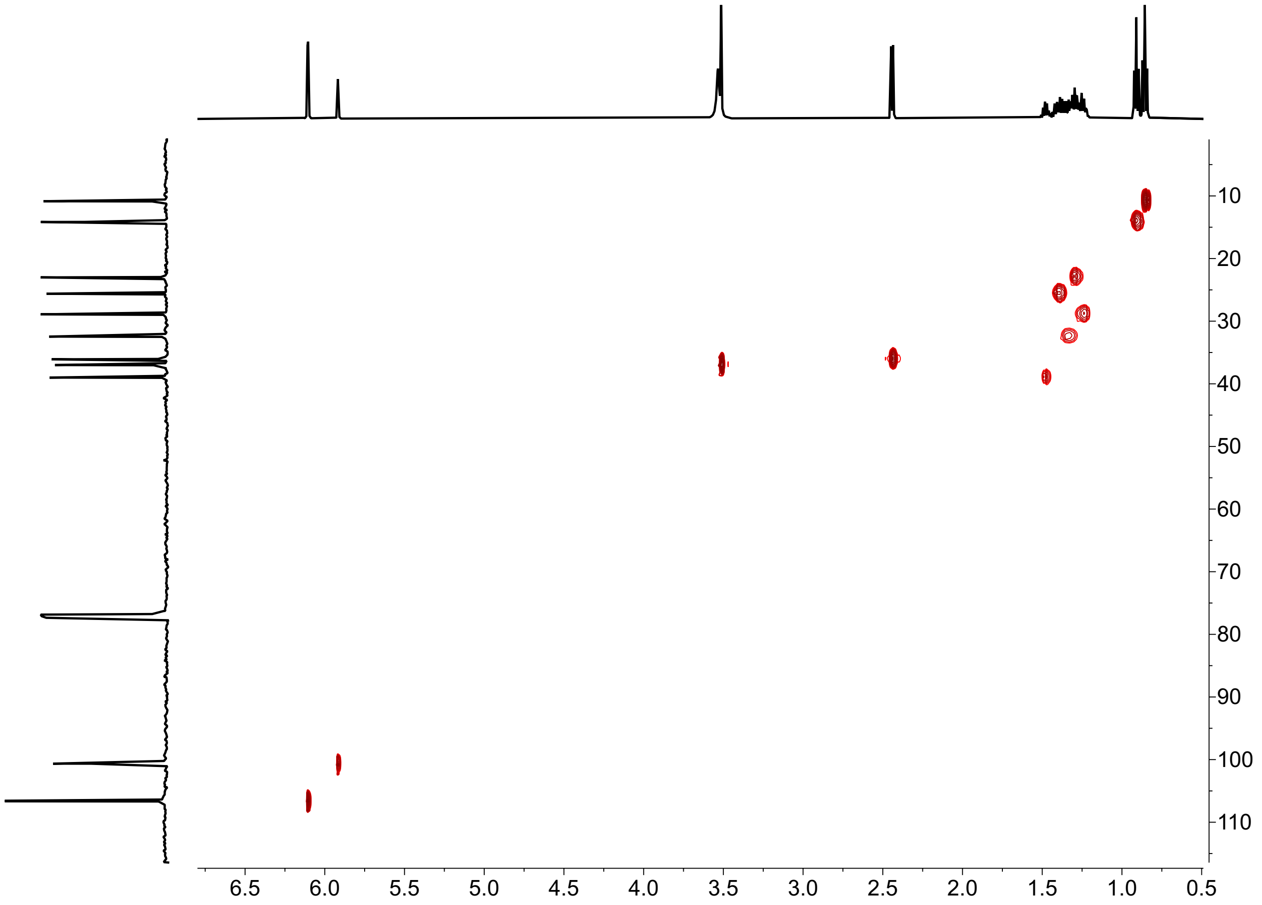

HSQC-NMR spectrum

9. Synthesis of trimer DDD

Trimer **DDD** was prepared as reported in the following scheme:

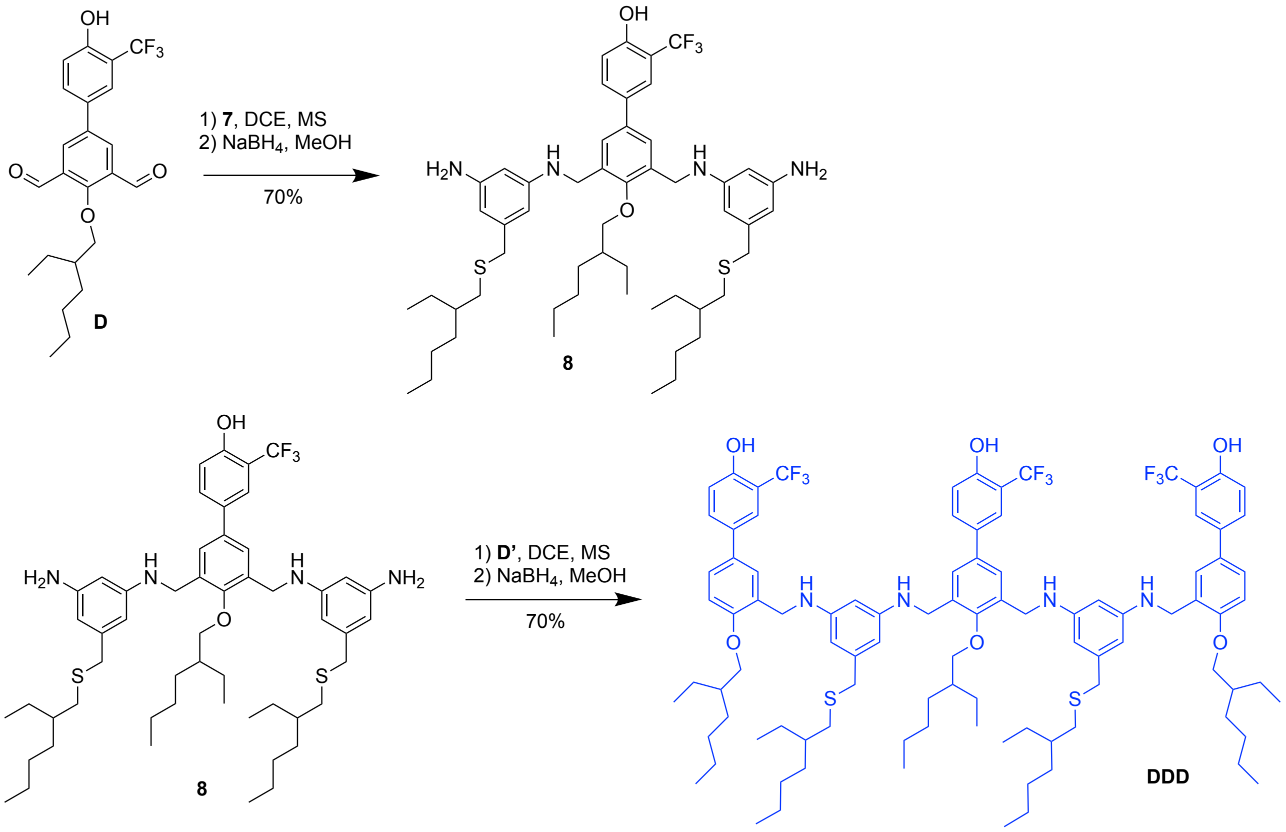

9.1 Synthesis of 8.

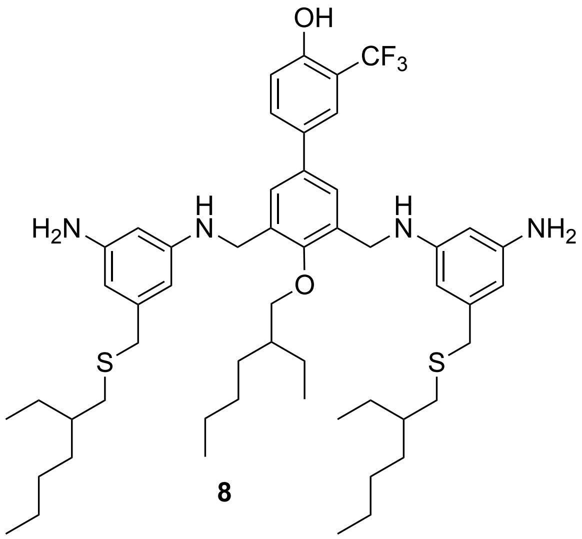

Monomer **D** (123 mg, 0.302 mmol, 1 equiv) and aniline **7** (722 mg, 2.718, 9 equiv) were dissolved in dry DCE (2.5 mL). Molecular sieves (3A) were added and the mixture was mixed overnight on an orbital shaker, under N_2 atmosphere. Then, $NaBH_4$ (114 mg, 3.020 mmol, 10 equiv) and MeOH (2.5 mL) were added and the mixture was shaken for 2 hours (^{1}H-NMR was used to confirm fully imine

reduction). Then the solution was filtered, MS were washed with DCM and a saturated solution of $NaHCO_3$ was added. The mixture was stirred until complete solubilisation, then the organic phase was extracted and the aqueous phase was washed (3x) with EtOAc. The organic layers were collected and dried over $MgSO_4$, the solvent was evaporated and the residue was purified *via* combiflash chromatography (PE:EtOAc), giving the desired compound **8** (195 mg, 70% yield).

^{1}H NMR (500 MHz, $CDCl_3$) δ 7.51 (d, *J* = 2.3 Hz, 1H), 7.41 – 7.31 (m, 2H), 7.24 (d, *J* = 8.5 Hz, 1H), 6.54 (d, *J* = 8.4 Hz, 1H), 6.16 (s, 2H), 6.10 (s, 2H), 5.91 (s, 2H), 4.35 (s, 4H), 3.79 (d, *J* = 5.9 Hz, 2H), 3.51 (s, 4H), 2.41 (d, *J* = 6.2 Hz, 4H), 1.80 – 1.70 (m, 1H), 1.62 – 1.13 (m, 26H), 0.92 (t, *J* = 7.6 Hz, 3H), 0.87 (t, *J* = 7.1 Hz, 9H), 0.81 (t, *J* = 7.5 Hz, 6H).
^{13}C NMR (126 MHz, $CDCl_3$) δ 171.47, 154.77, 153.43, 149.16, 145.78, 140.19, 135.22, 132.91, 132.09, 131.40, 126.68, 124.95, 123.87 (d, *J* = 272.5 Hz), 117.59, 116.73 (q, *J* = 30.3 Hz), 107.01, 106.34, 103.67, 98.32, 60.49, 43.41, 40.67, 38.90, 37.06, 36.04, 32.32, 30.27, 29.15, 28.78, 25.47, 23.63, 22.97, 22.89, 13.61, 11.17, 10.71.
^{19}F NMR (471 MHz, $CDCl_3$) δ -61.76.
HRMS (ASAP+): Calculated for $C_{53}H_{78}N_4O_2F_3S_2$ 923.5518 a.m.u., found 923.5536 a.m.u.
FT-IR (ATR): ν_{max} / cm^{-1} 3383, 3043, 2958, 2926, 2871, 2858, 2724, 1715, 1599, 1515, 1445, 1407, 1379, 1338, 1266, 1201, 1103, 1078, 1052, 956, 907, 882, 767, 687, 600.

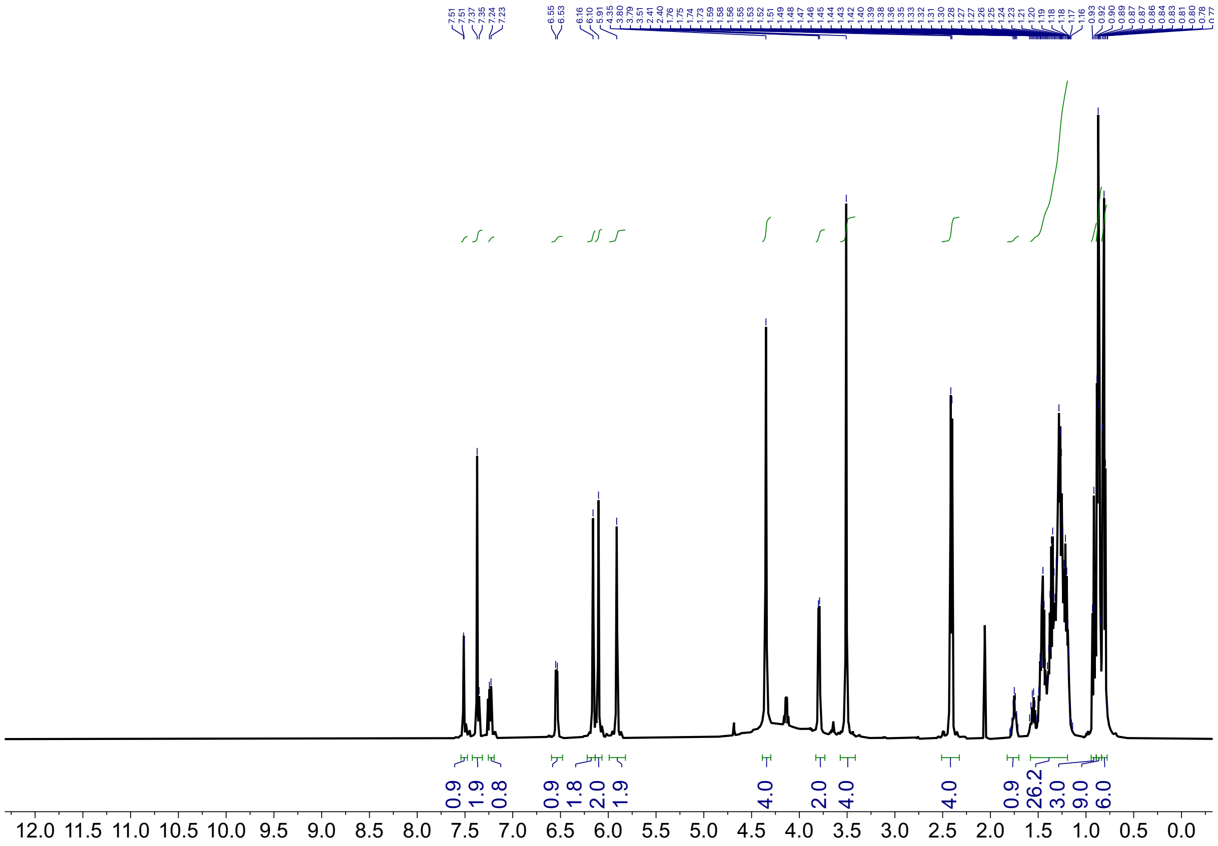

^{1}H-NMR spectrum

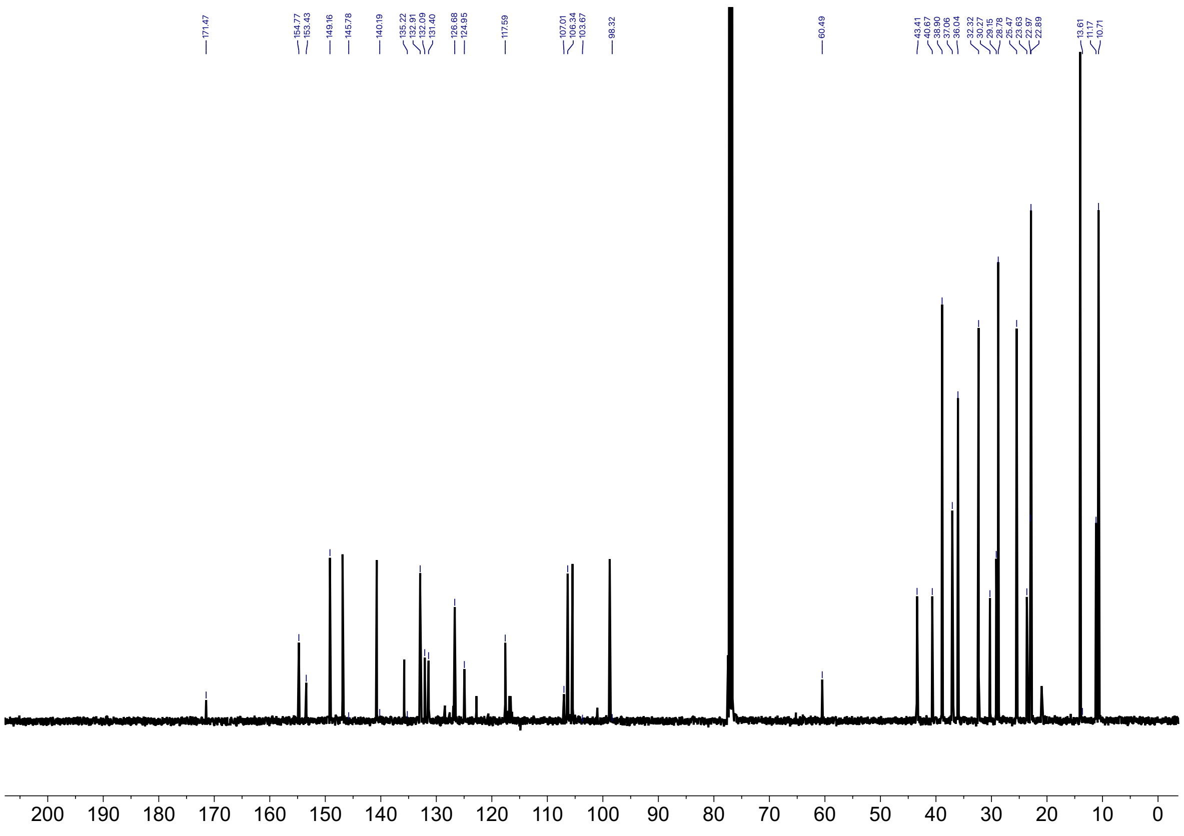

^{13}C-NMR spectrum

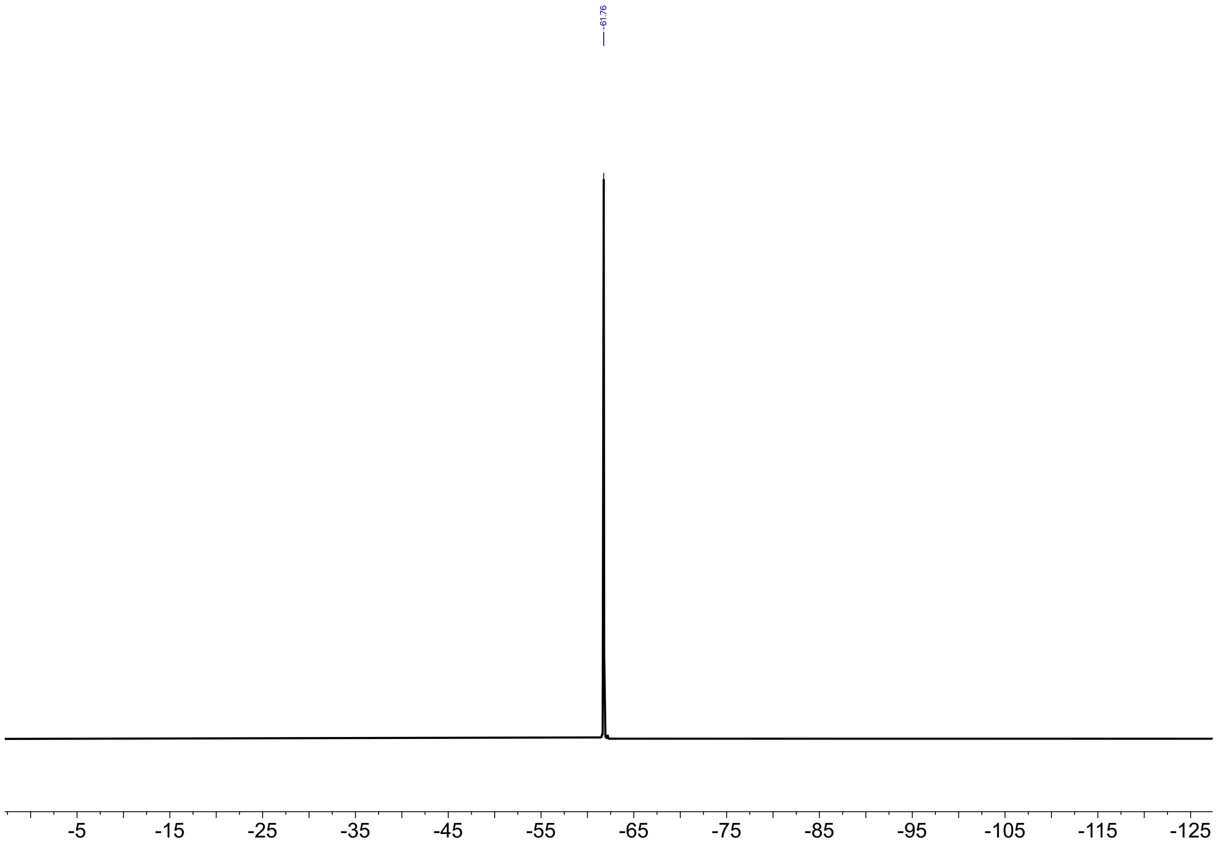

^{19}F-NMR spectrum

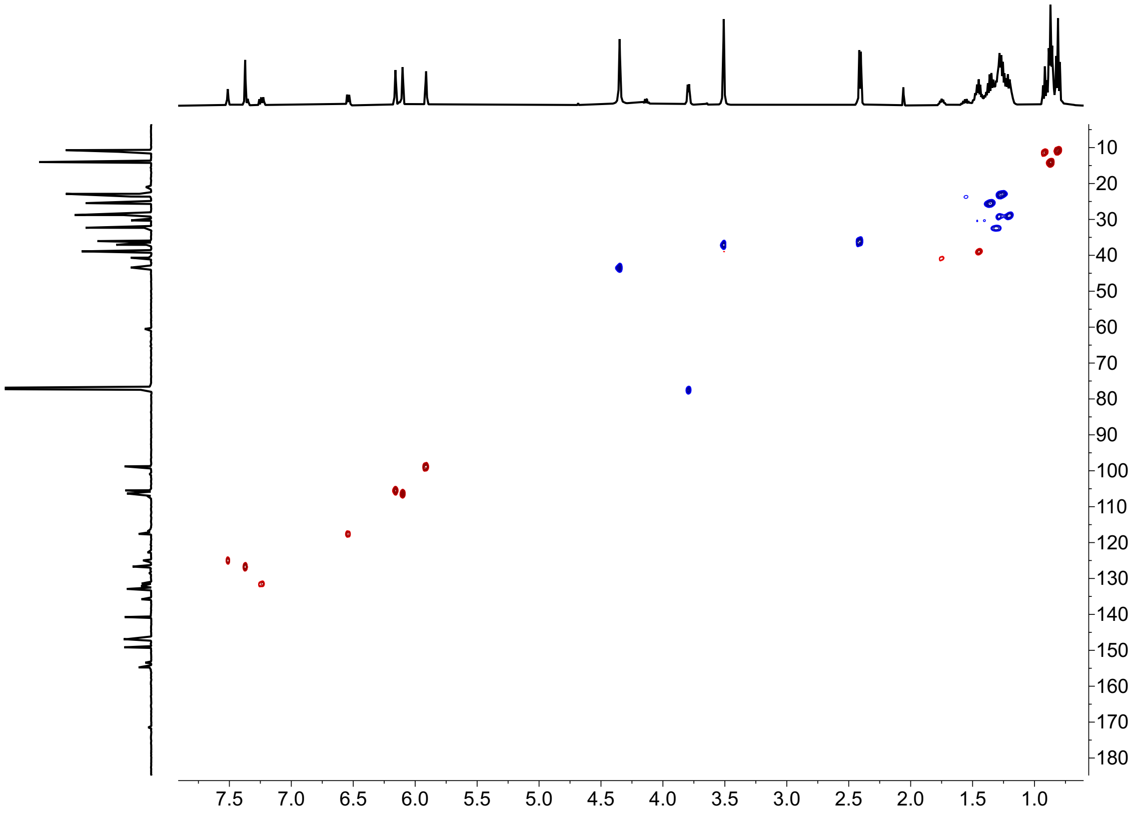

HSQC-NMR spectrum

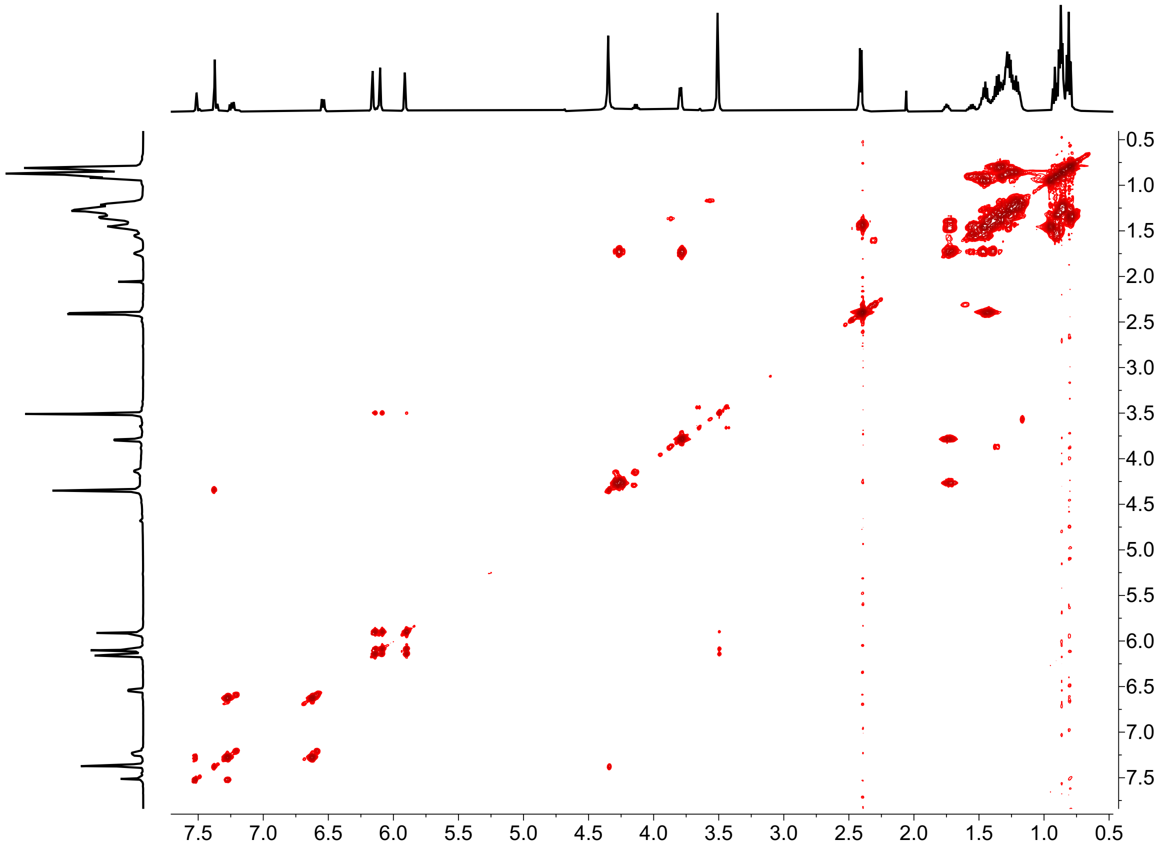

COSY-NMR spectrum

9.2 Synthesis of DDD.

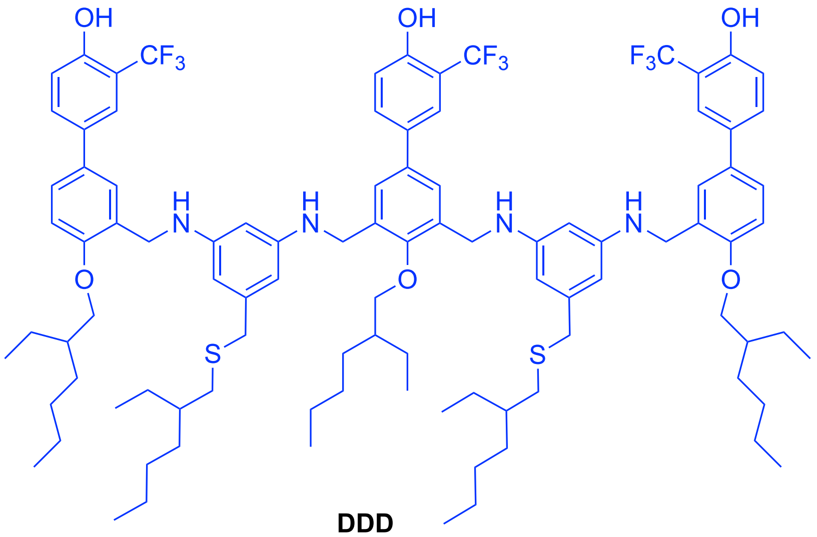

DDD

Compound **8** (120 mg, 0.164 mmol, 1 equiv) and donor monomer **D'** (260 mg, 0.657, 4 equiv) were dissolved in dry DCE (2 mL). Molecular sieves (3A) were added and the mixture was mixed overnight on an orbital shaker, under N_2 atmosphere. Then, $NaBH_4$ (70 mg, 1.640 mmol, 10 equiv) and MeOH (2.5 mL) were added and the mixture was shaken for 2 hours (^{1}H-NMR was used to confirm fully imine reduction). Then the solution was filtered, MS were washed with DCM and a saturated solution of $NaHCO_3$ was added. The mixture was stirred until complete solubilisation, then the organic phase was extracted and the aqueous phase was washed (3x) with EtOAc. The organic layers were collected and dried over $MgSO_4$, the solvent was evaporated and the residue was purified via combiflash chromatography (PE:EtOAc), giving the desired compound **DDD** (192 mg, 70% yield).

^{1}H NMR (500 MHz, $CDCl_3$-*d*) δ 7.56 (s, 2H), 7.50 (s, 1H), 7.44 – 7.28 (m, 9H), 7.26 (s, 1H), 6.90 (d, *J* = 8.5 Hz, 2H), 6.80 – 6.71 (m, 3H), 6.12 (s, 4H), 5.95 (s, 2H), 4.34 (s, 8H), 3.92 (t, *J* = 4.8 Hz, 4H), 3.76 (d, *J* = 5.9 Hz, 2H), 3.52 (s, 4H), 2.38 (d, *J* = 6.3 Hz, 4H), 1.80 – 1.64 (m, 3H), 1.57 – 1.13 (m, 46H), 0.93 (t, *J* = 7.5 Hz, 6H), 0.91 – 0.87 (m, 6H), 0.86 – 0.82 (m, 9H), 0.81 – 0.74 (m, 9H).
^{13}C NMR (126 MHz, $CDCl_3$) δ 156.54, 154.96, 152.47, 151.98, 149.25, 148.15, 140.51, 135.52, 133.28, 132.97, 132.82, 131.58, 131.52, 131.37, 127.80, 127.38, 126.90, 126.29, 124.91 (d, *J* = 4.9 Hz), 124.65 (d, *J* = 4.7 Hz), 123.99 (d, *J* = 272.4 Hz), 117.86, 111.25, 104.92, 104.39, 97.07, 70.29, 44.15, 43.58, 40.62, 39.31, 38.90, 37.29, 35.54, 32.28, 30.62, 30.20, 29.08, 29.01, 28.76, 25.42, 23.98, 23.54, 22.95, 22.89, 22.86, 13.99, 13.94, 11.07, 10.67.
^{19}F NMR (471 MHz, $CDCl_3$) δ -61.56, -61.59.
HRMS (ASAP+): Calculated for $C_{97}H_{128}F_9N_4O_6S_2$ 1679,9126 a.m.u., found 1679,9118 a.m.u.
FT-IR (ATR): ν_{max} / cm^{-1} 3408, 2957, 2926, 2871, 2859, 1601, 1495, 1441, 1407, 1380, 1334, 1292, 1247, 1160, 1130, 1052.

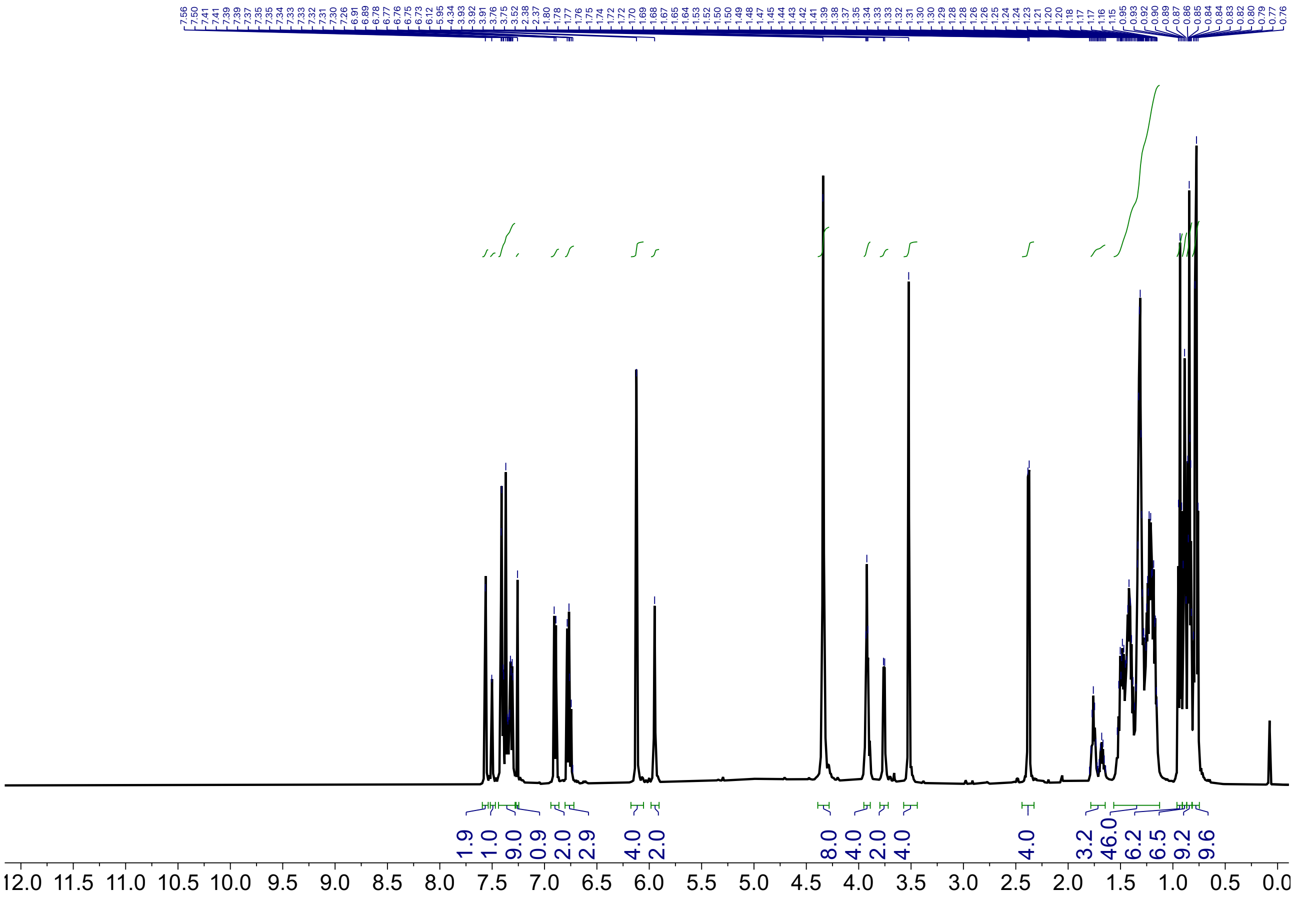

^{1}H-NMR spectrum

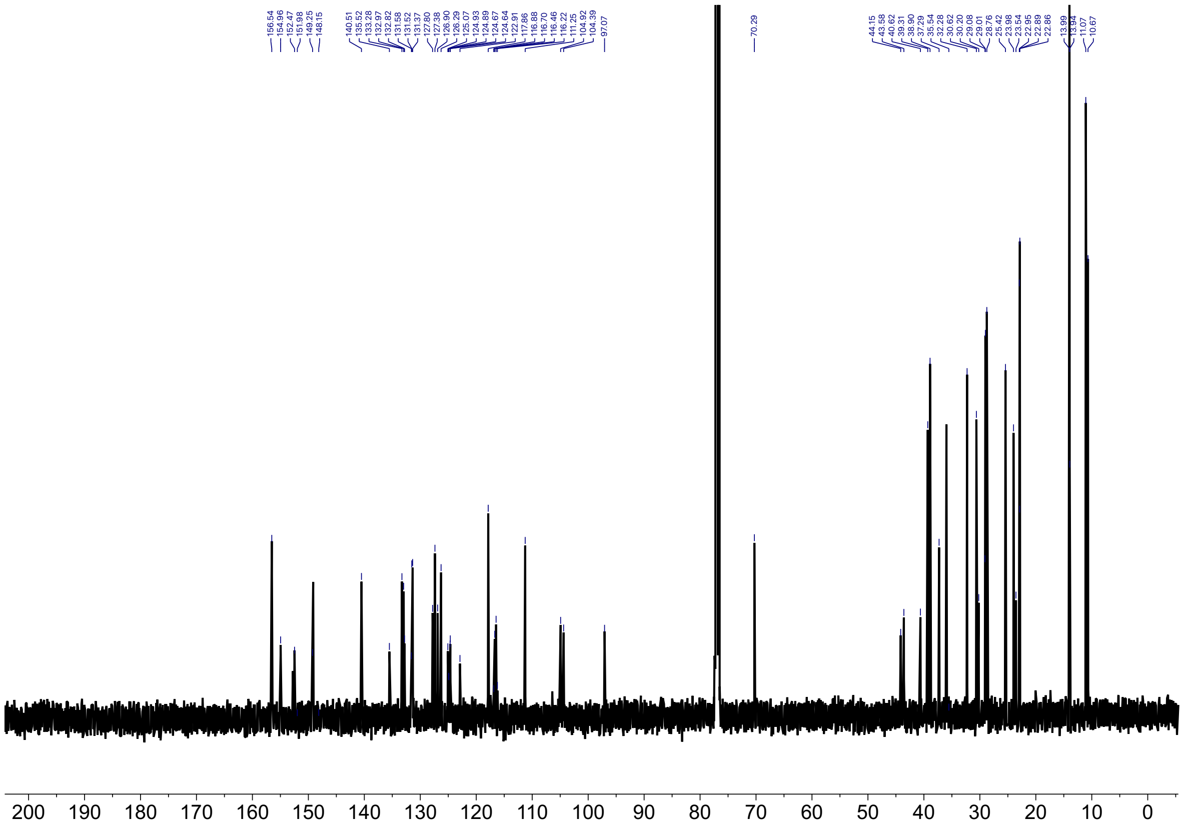

^{13}C-NMR spectrum

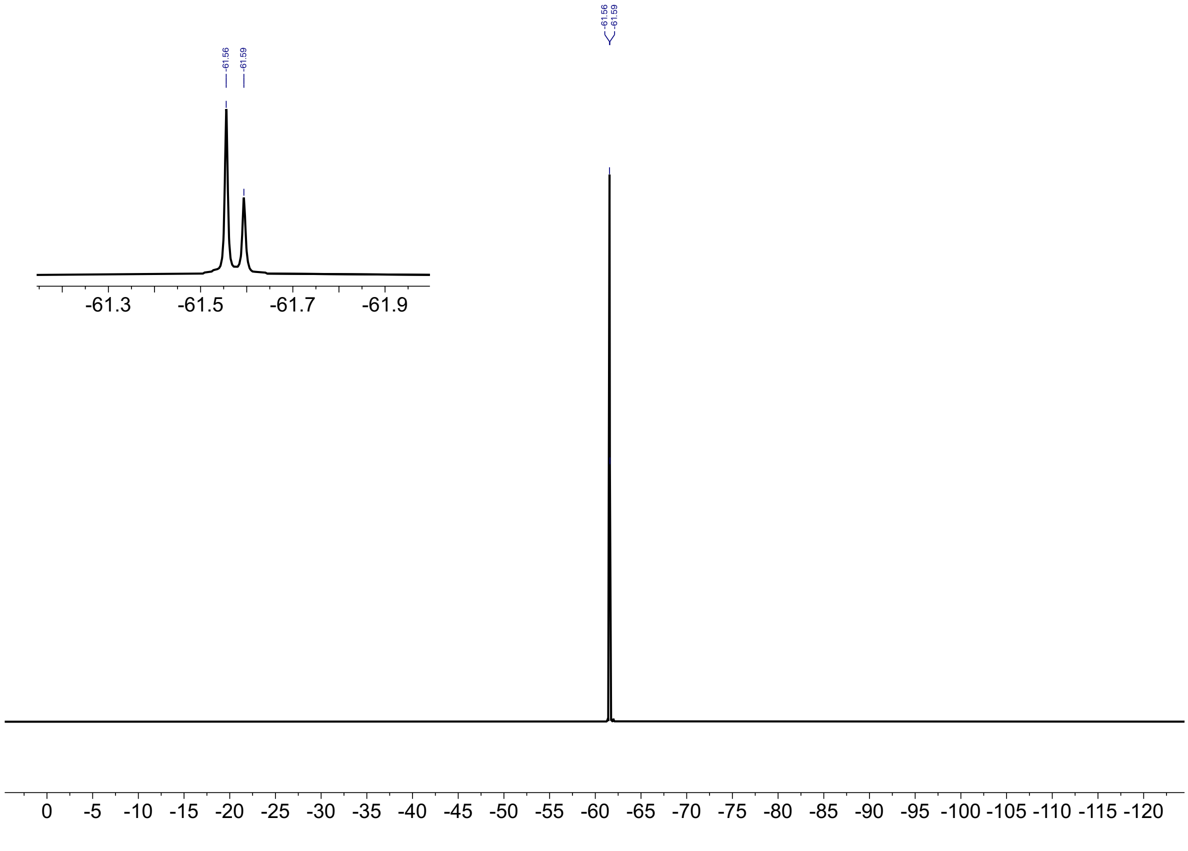

^{19}F-NMR spectrum

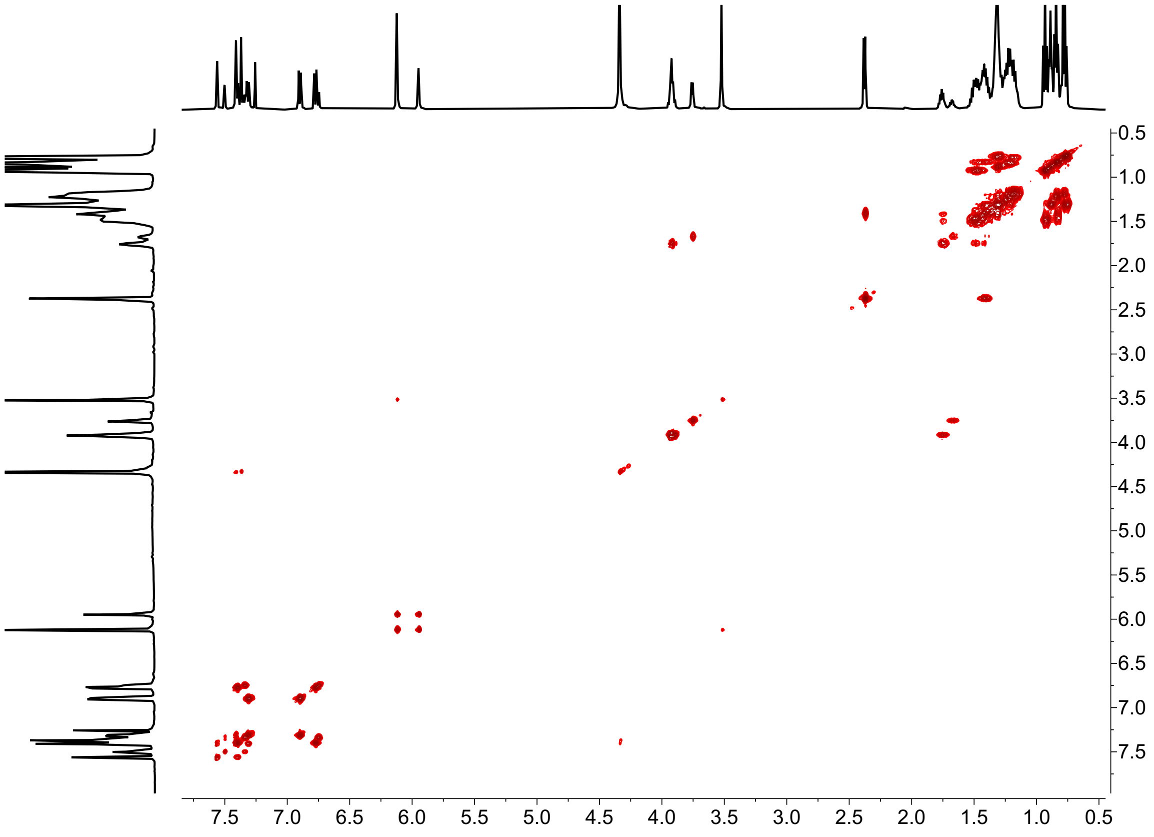

COSY-NMR spectrum

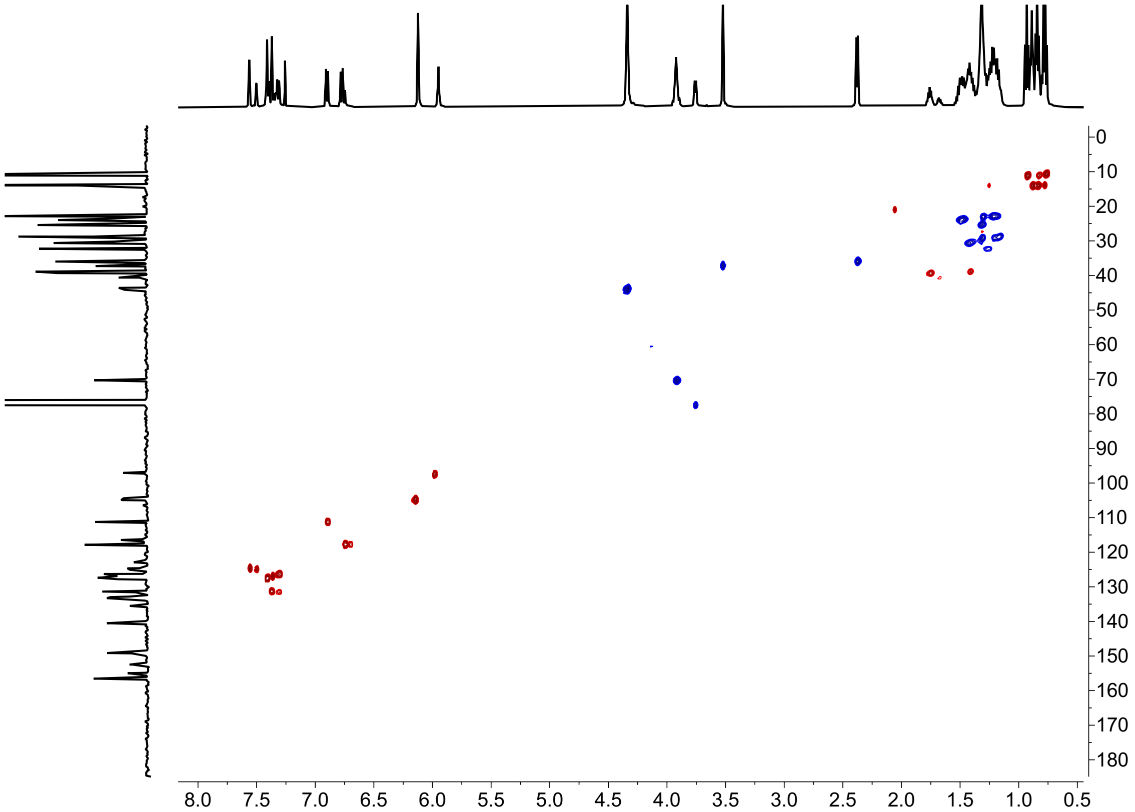

HSQC-NMR spectrum

10. Synthesis of trimer AAA

Trimer **AAA** was prepared as reported in the following scheme:

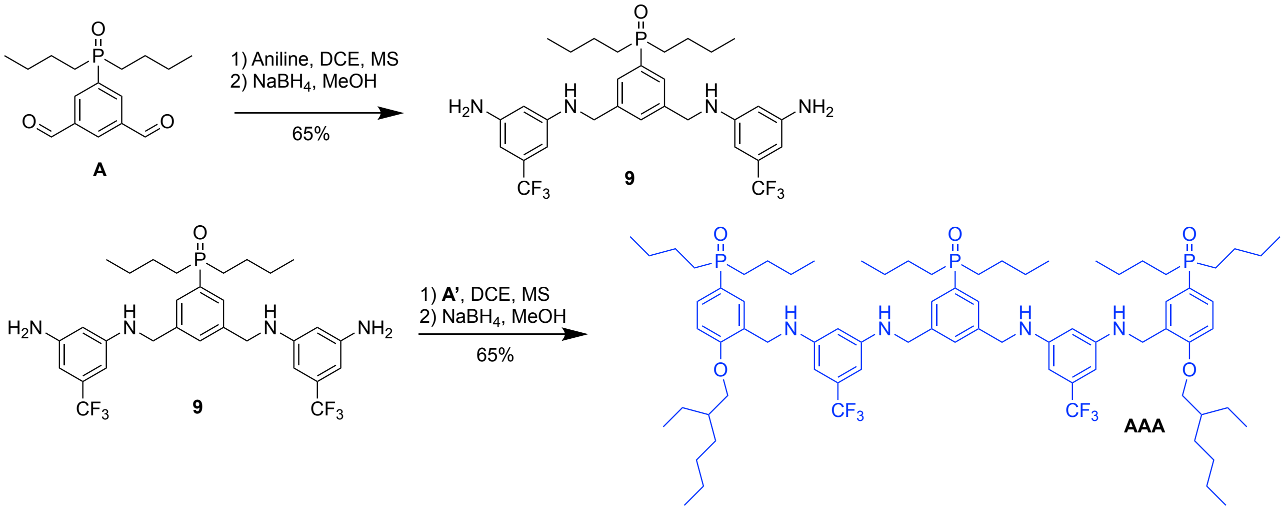

10.1 Synthesis of 9.

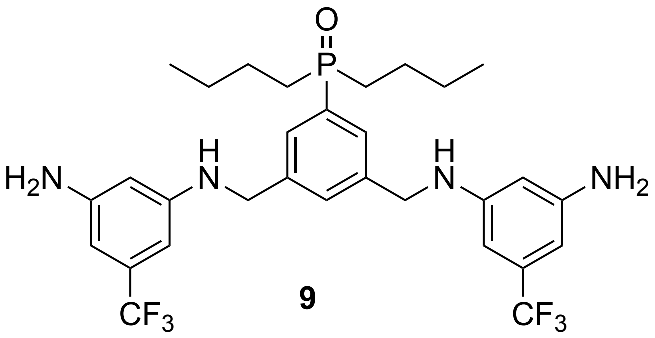

9

Monomer **A** (105 mg, 0.357 mmol, 1 equiv) and aniline **7** (565 mg, 3.210, 9 equiv) were dissolved in dry DCE (2.5 mL). Molecular sieves (3A) were added and the mixture was mixed overnight on an orbital shaker, under N_2 atmosphere. Then, $NaBH_4$ (114 mg, 3.570 mmol, 10 equiv) and MeOH (1 mL) were added and the mixture was shaken for 2 hours (^{1}H-NMR was used to confirm fully imine reduction). Then the solution was filtered, MS were carefully washed with DCM and a saturated solution of $NaHCO_3$ was added. The mixture was stirred until complete solubilisation, then the organic phase was extracted and the aqueous phase was washed (3x) with EtOAc. The organic layers

were collected and dried over $MgSO_4$, the solvent was evaporated and the residue was purified via combiflash chromatography (PE:EtOAc), giving the desired compound **9** (142 mg, 65% yield).

^{1}H NMR (500 MHz, Chloroform-*d*) δ 7.55 (d, *J* = 10.8 Hz, 2H), 7.46 (s, 1H), 6.25 (s, 2H), 6.22 (s, 2H), 5.96 (s, 2H), 4.33 (s, 6H), 3.77 (bs, 4H), 1.99 – 1.86 (m, 2H), 1.84 – 1.76 (m, 2H), 1.57 – 1.50 (m, 2H), 1.38 – 1.21 (m, 6H), 0.83 (t, *J* = 7.2 Hz, 6H).

^{13}C NMR (126 MHz, Chloroform-*d*) δ 148.36 (d, *J* = 137.6 Hz), 140.18 (d, *J* = 10.9 Hz), 133.60 (d, *J* = 90.3 Hz), 132.31 (q, *J* = 31.5 Hz), 129.01 (d, *J* = 2.6 Hz), 127.95 (d, *J* = 9.1 Hz), 124.11 (q, *J* = 272.4 Hz), 101.40, 100.29 (q, *J* = 4.2 Hz), 47.50, 29.40 (d, *J* = 68.4 Hz), 23.91 (d, *J* = 14.6 Hz), 23.36 (d, *J* = 4.2 Hz), 13.37.

^{19}F NMR (471 MHz, $CDCl_3$) δ -63.47.

^{31}P NMR (202 MHz, $CDCl_3$) δ 41.32.

HRMS (ES+): Calculated for $C_{30}H_{38}N_4OF_6P$ 615,2687 a.m.u., found 615,2674 a.m.u.

FT-IR (ATR): ν_{max} / cm^{-1} 3428, 3327, 3219, 3071, 3040, 2961, 2933, 2873, 1608, 1519, 1487, 1455, 1427, 1385, 1330, 1269, 1210, 1157, 1112, 1085, 989, 995, 904, 888, 829, 735, 695.

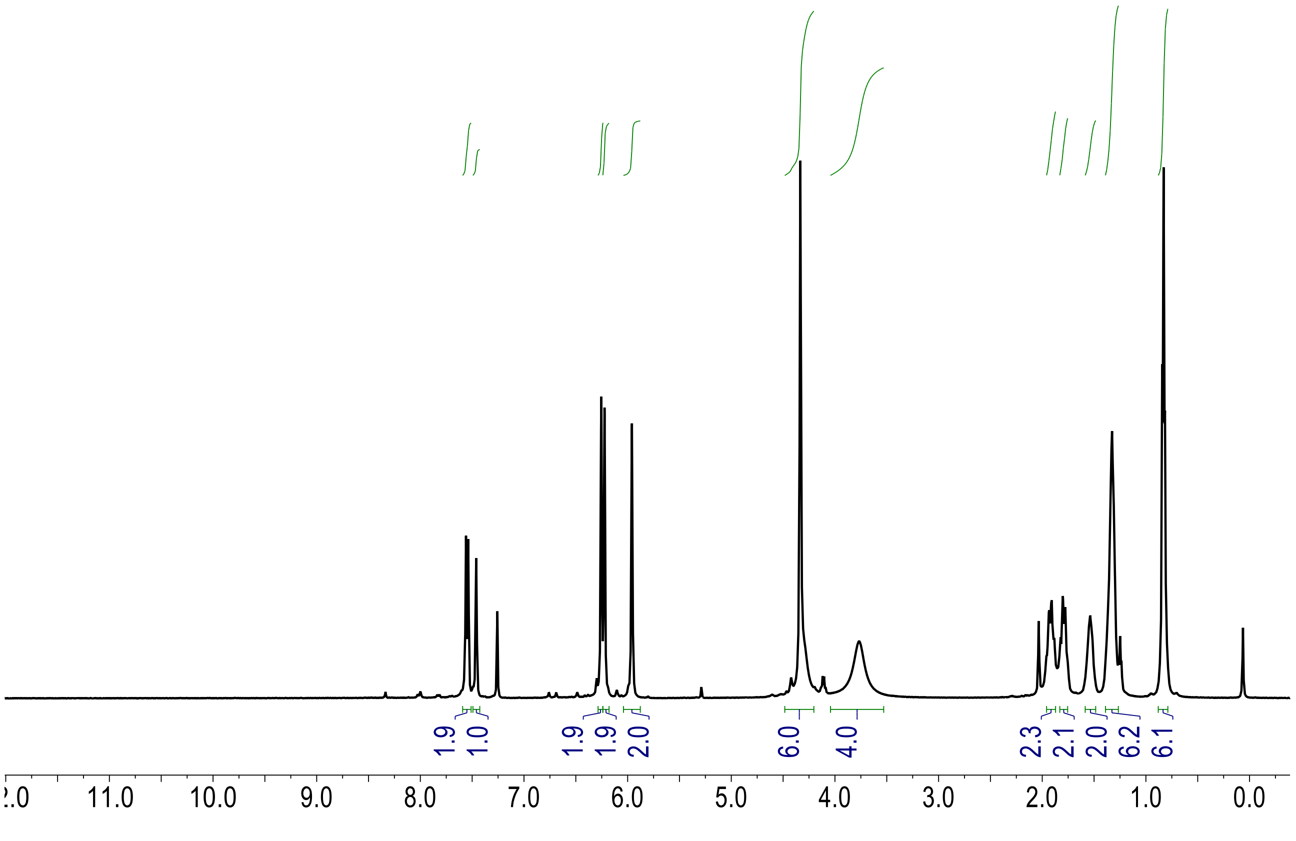

^{1}H-NMR spectrum

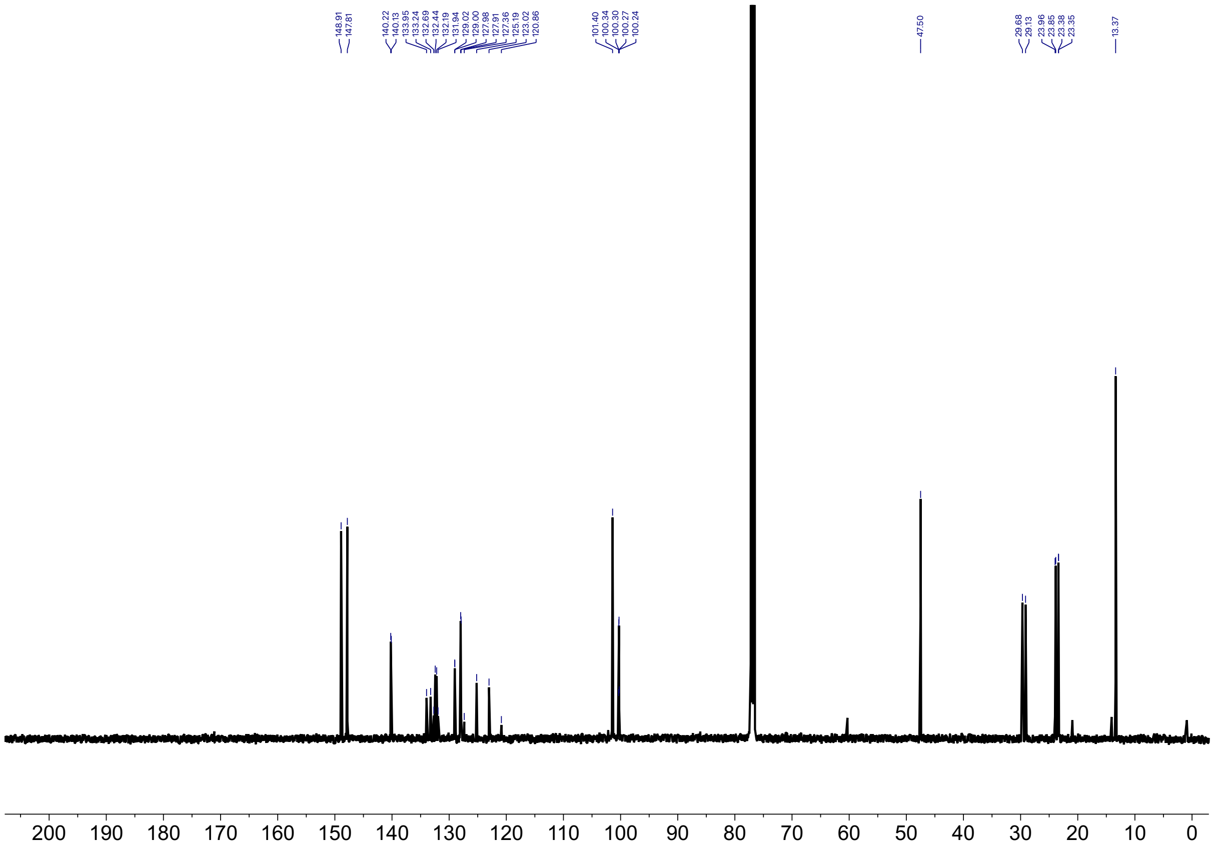

^{13}C-NMR spectrum

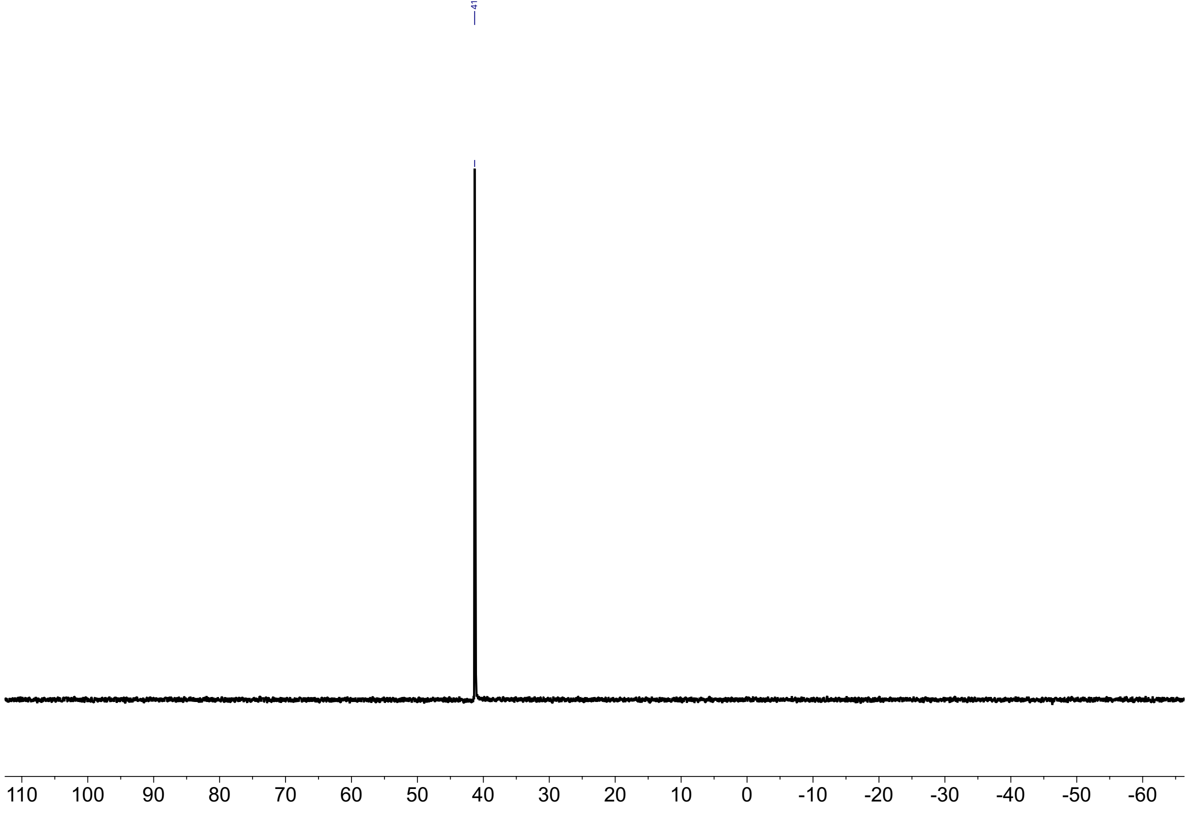

^{31}P-NMR spectrum

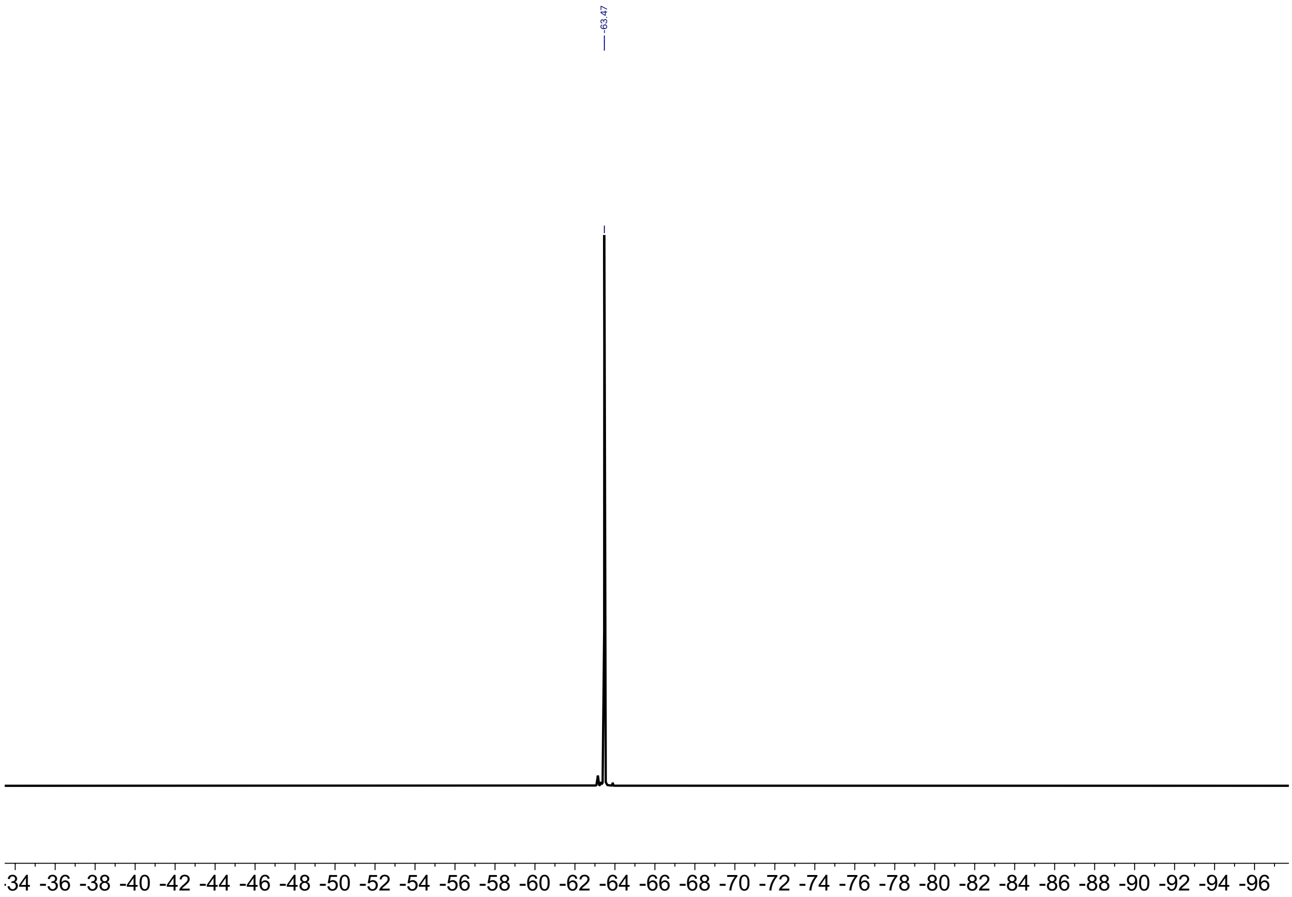

^{19}F-NMR spectrum

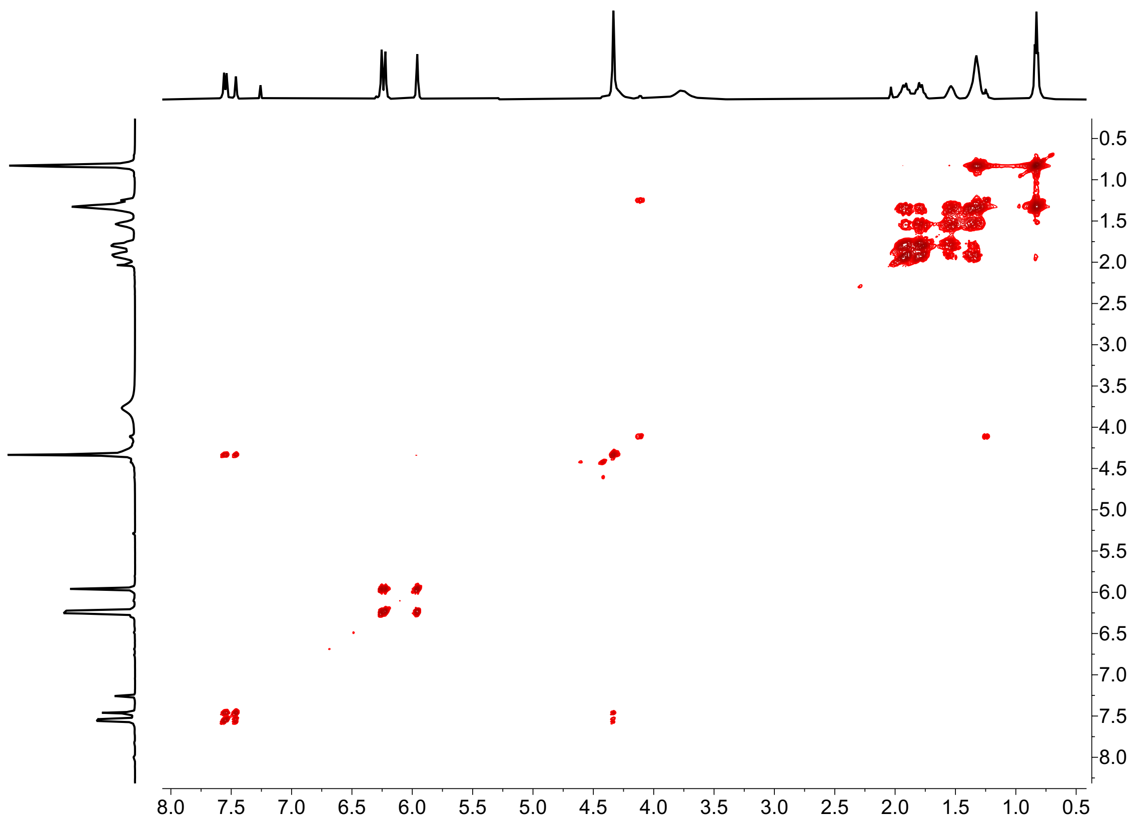

COSY-NMR spectrum

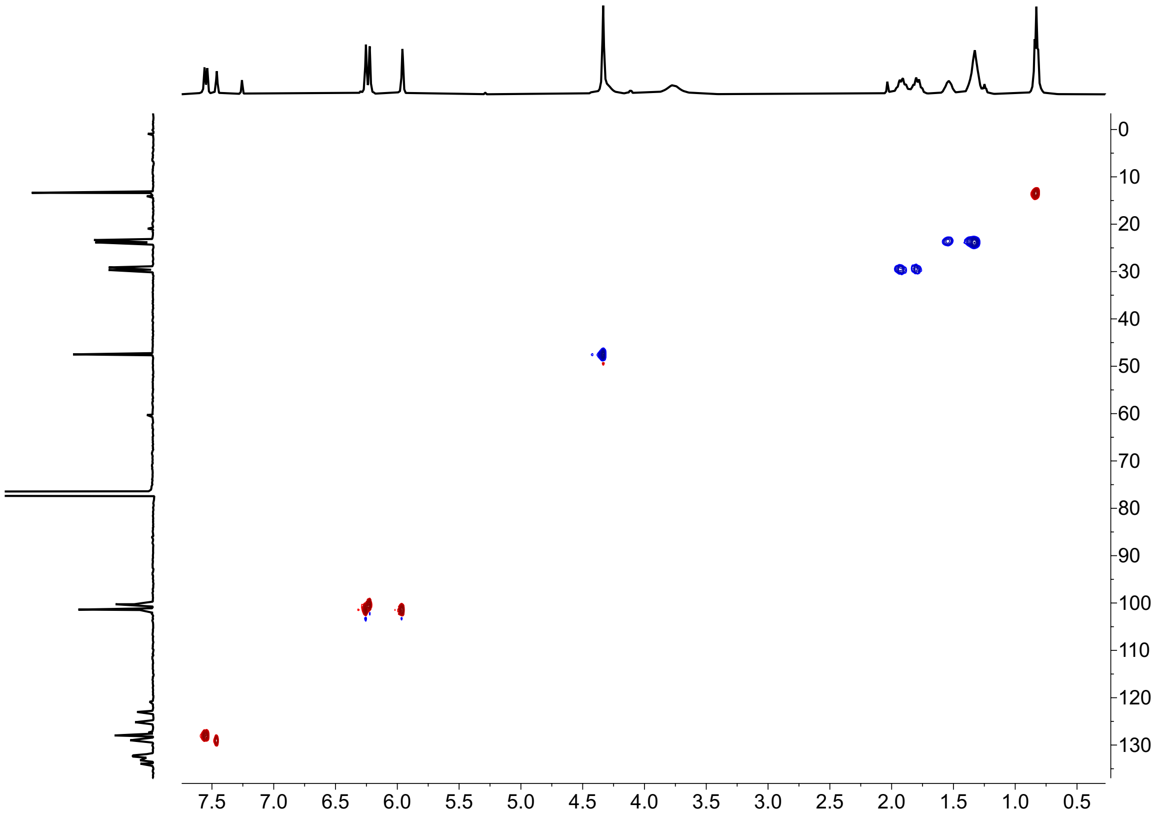

HSQC-NMR spectrum

10.2 Synthesis of AAA.

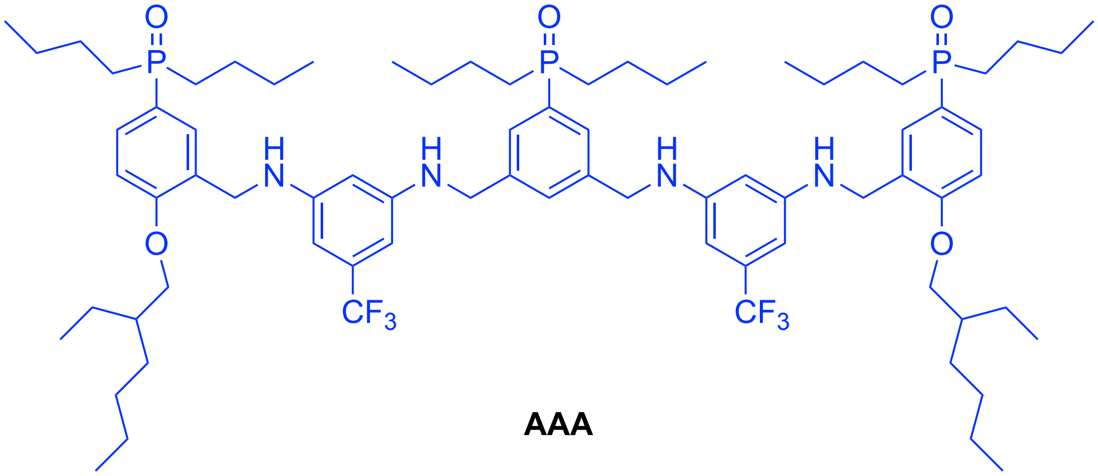

AAA

Compound **9** (40 mg, 0.065 mmol, 1 equiv) and donor monomer **A'** (102 mg, 0.260, 4 equiv) were dissolved in dry DCE (1 mL). Molecular sieves (3A) were added and the mixture was mixed overnight on an orbital shaker, under N_2 atmosphere. Then, $NaBH_4$ (50 mg, 1.300 mmol, 10 equiv) and MeOH : DCE (2.5 mL, 7:3) were added and the mixture was shaken for 2 hours (^{1}H-NMR was used to confirm fully imine reduction). Then the solution was filtered, MS were carefully washed with DCM and a saturated solution of $NaHCO_3$ was added. The mixture was stirred until complete solubilisation, then the organic phase was extracted and the aqueous phase was washed (3x) with EtOAc. The organic layers were collected and dried over $MgSO_4$, the solvent was evaporated and the residue was purified via combiflash chromatography (PE:EtOAc), giving the desired compound **AAA** (59 mg, 65% yield).

^{1}H NMR (500 MHz, $CDCl_3$-*d*) δ 7.59 – 7.51 (m, 7H), 6.95 (d, *J* = 8.7 Hz, 2H), 6.15 (d, *J* = 4.8 Hz, 4H), 6.00 (s, 2H), 4.32 (s, 4H), 4.29 (s, 4H), 3.93 (d, *J* = 5.5 Hz, 4H), 2.43 (bs, 4H), 1.98 – 1.68 (m, 14H), 1.58 – 1.19 (m, 42H), 0.92 (t, *J* = 7.5 Hz, 6H), 0.89 – 0.85 (m, 6H), 0.84 – 0.76 (m, 18H).
^{13}C NMR (126 MHz, $CDCl_3$-*d*) δ 159.53, 149.08 (d, *J* = 17.7 Hz), 140.26 (d, *J* = 10.9 Hz), 133.34 (d, *J* = 90.5 Hz), 132.08 (q, *J* = 31.5 Hz), 131.22 (d, *J* = 9.6 Hz), 130.66 (d, *J* = 10.1 Hz), 129.37, 128.07 (d, *J* = 8.8 Hz), 127.37 (d, *J* = 11.2 Hz), 124.26 (q, *J* = 272.6 Hz), 110.95 (d, *J* = 12.5 Hz),123,1, 122,4, 99.46 (d, *J* = 3.5 Hz), 99.71, 99.07 (d, *J* = 3.9 Hz), 70.36, 47.59, 42.43, 39.25, 30.60, 29.88, 29.68, 29.33, 29.14, 28.97, 23.97, 23.95, 23.86, 23.37, 23.33, 22.88, 13.92, 13.40, 11.05.
^{19}F NMR (471 MHz, $CDCl_3$) δ -63.43.
^{31}P NMR (202 MHz, $CDCl_3$) δ 41.46.
HRMS (ASAP+): Calculated for $C_{76}H_{116}N_4O_5F_6P_3$ 1371,8063 a.m.u., found 1371,8041 a.m.u.

FT-IR (ATR): ν_{max} / cm^{-1}3286, 2959, 2930, 2872, 1608, 1546, 1513, 1494, 1466, 1396, 1381, 1346, 1324, 1276, 1247, 1218, 1188, 1117, 1085, 908, 820, 805, 709, 696.

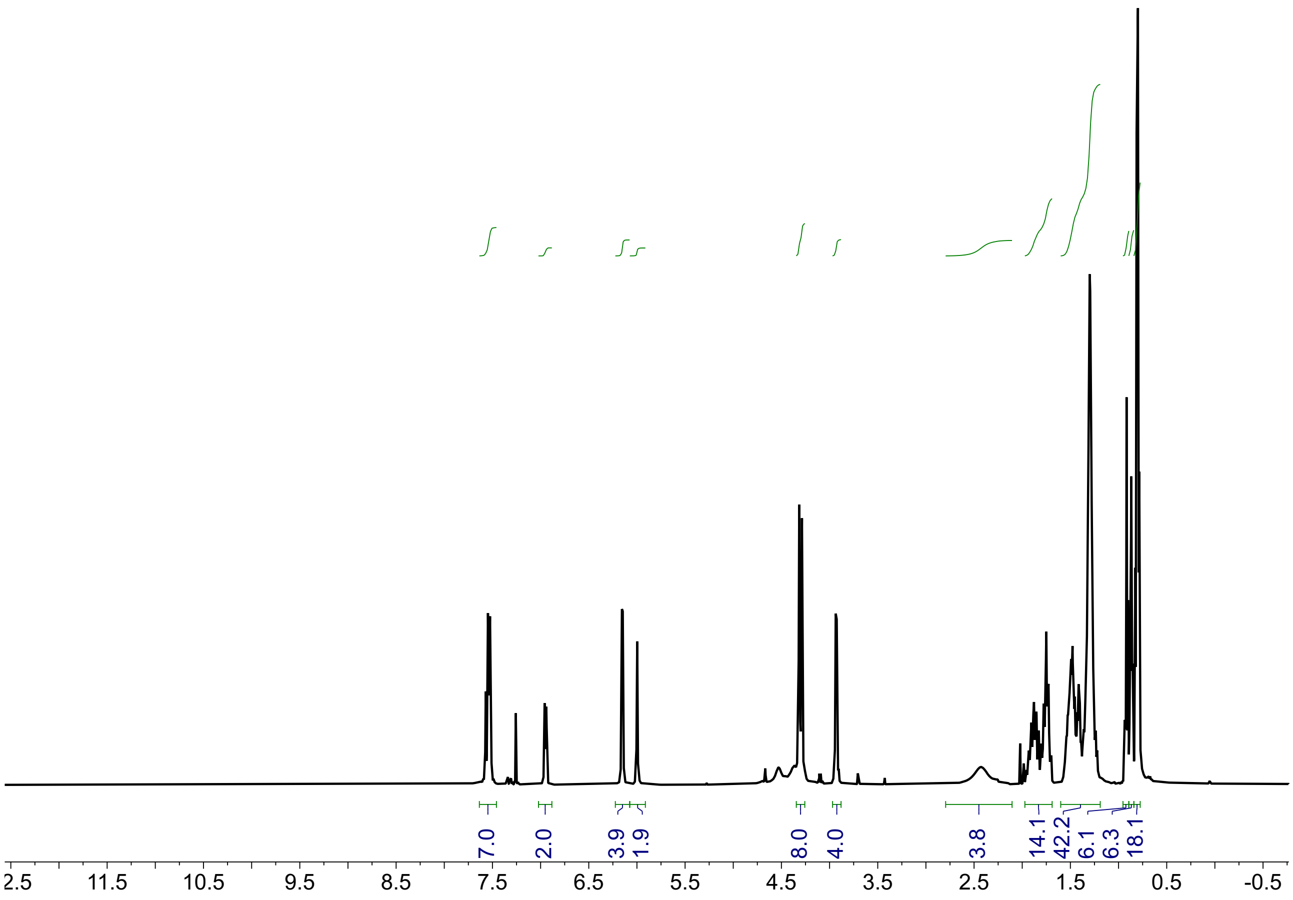

^{1}H-NMR spectrum

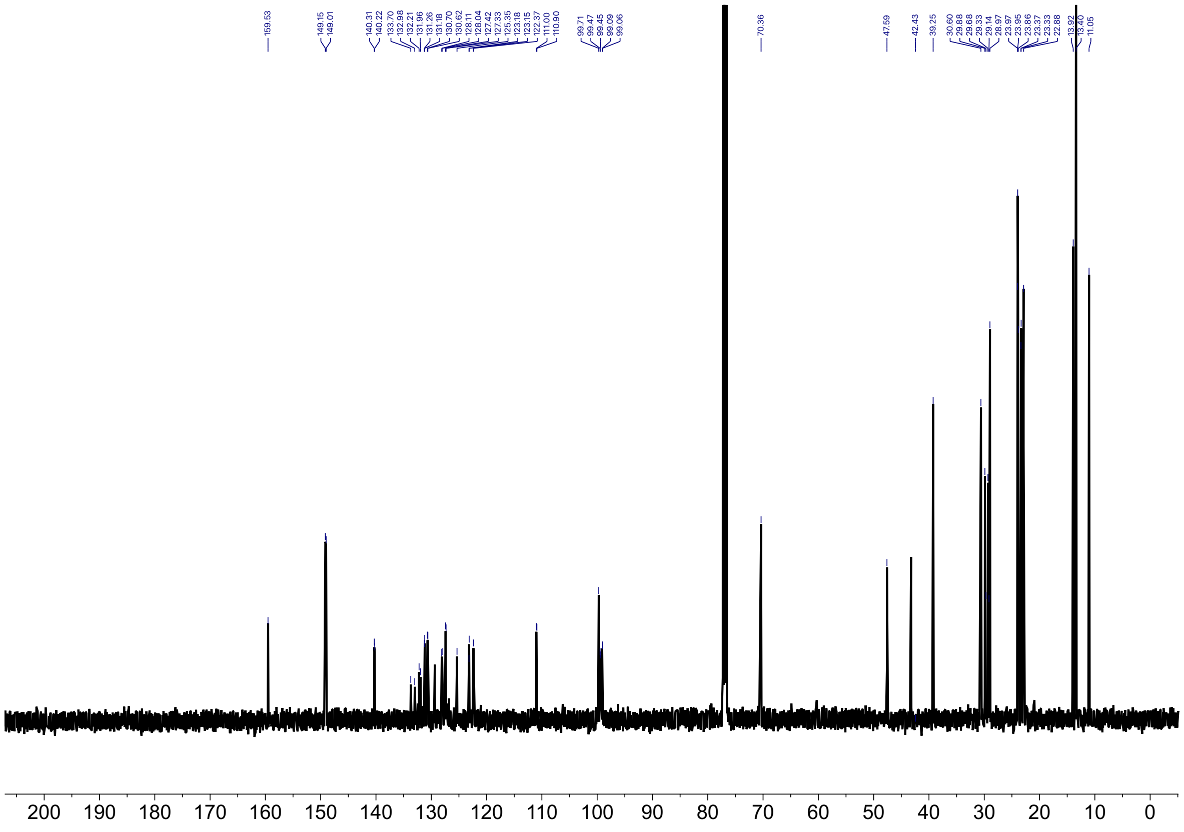

^{13}C-NMR spectrum

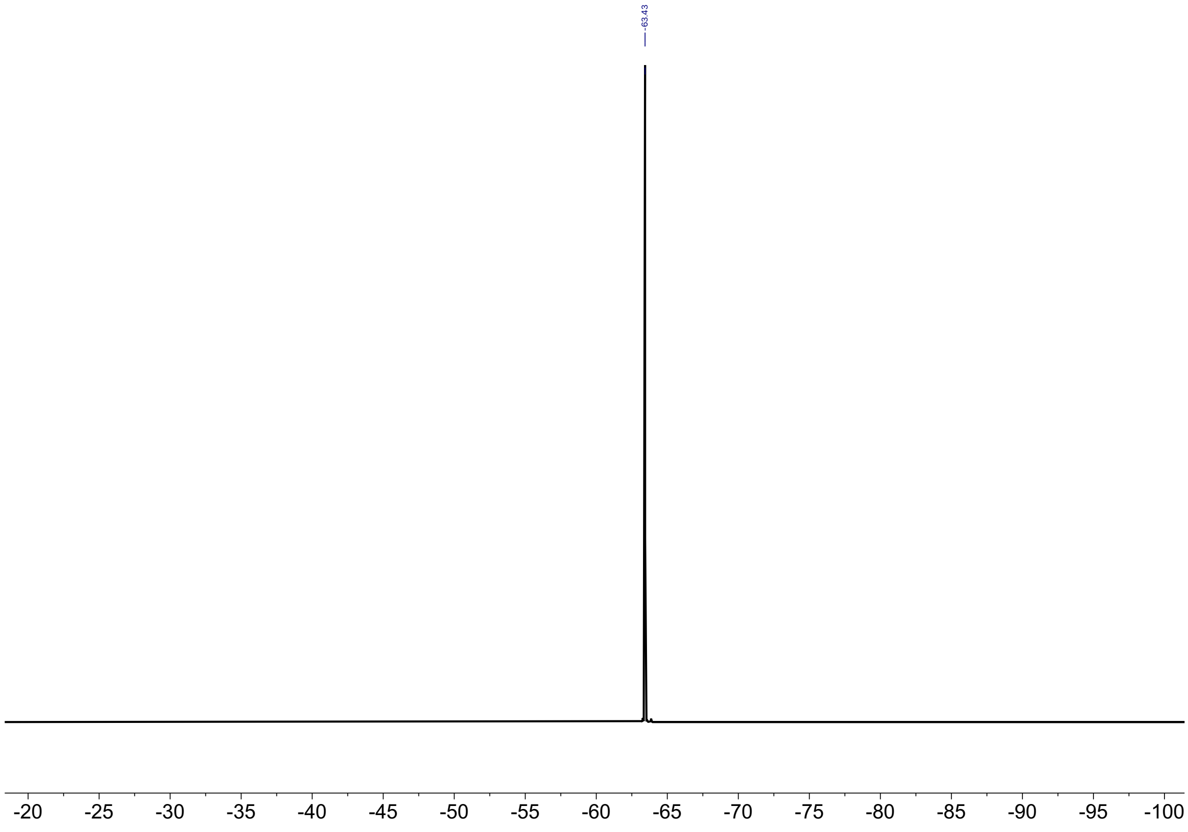

^{19}F-NMR spectrum

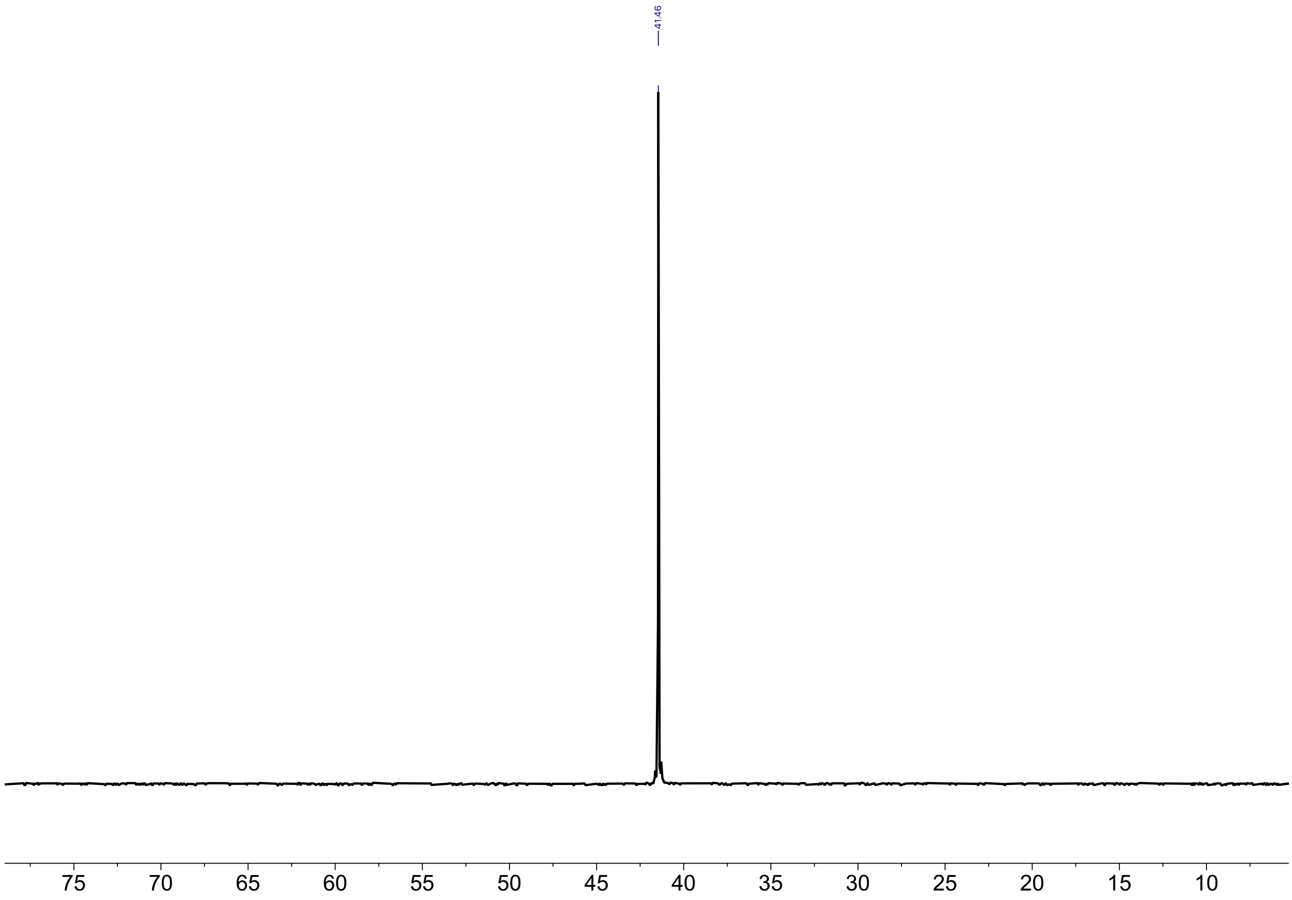

^{31}P-NMR spectrum

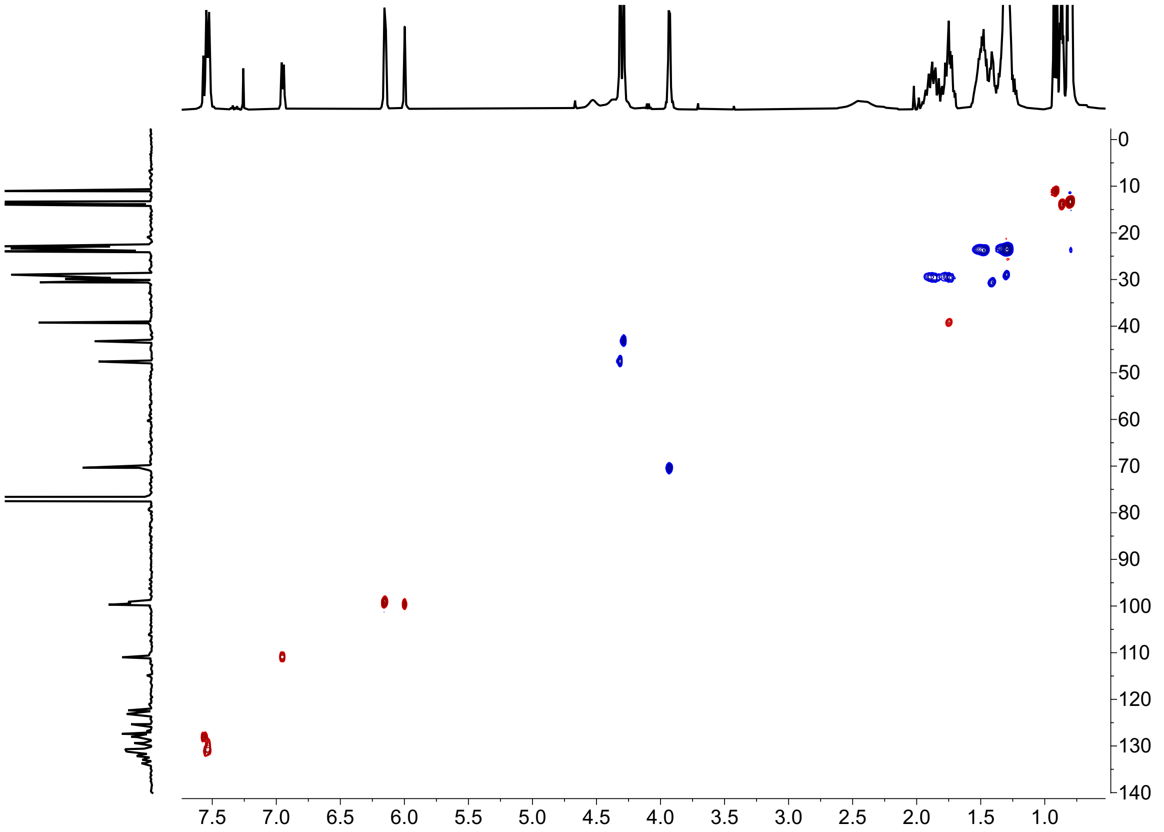

HSQC-NMR spectrum

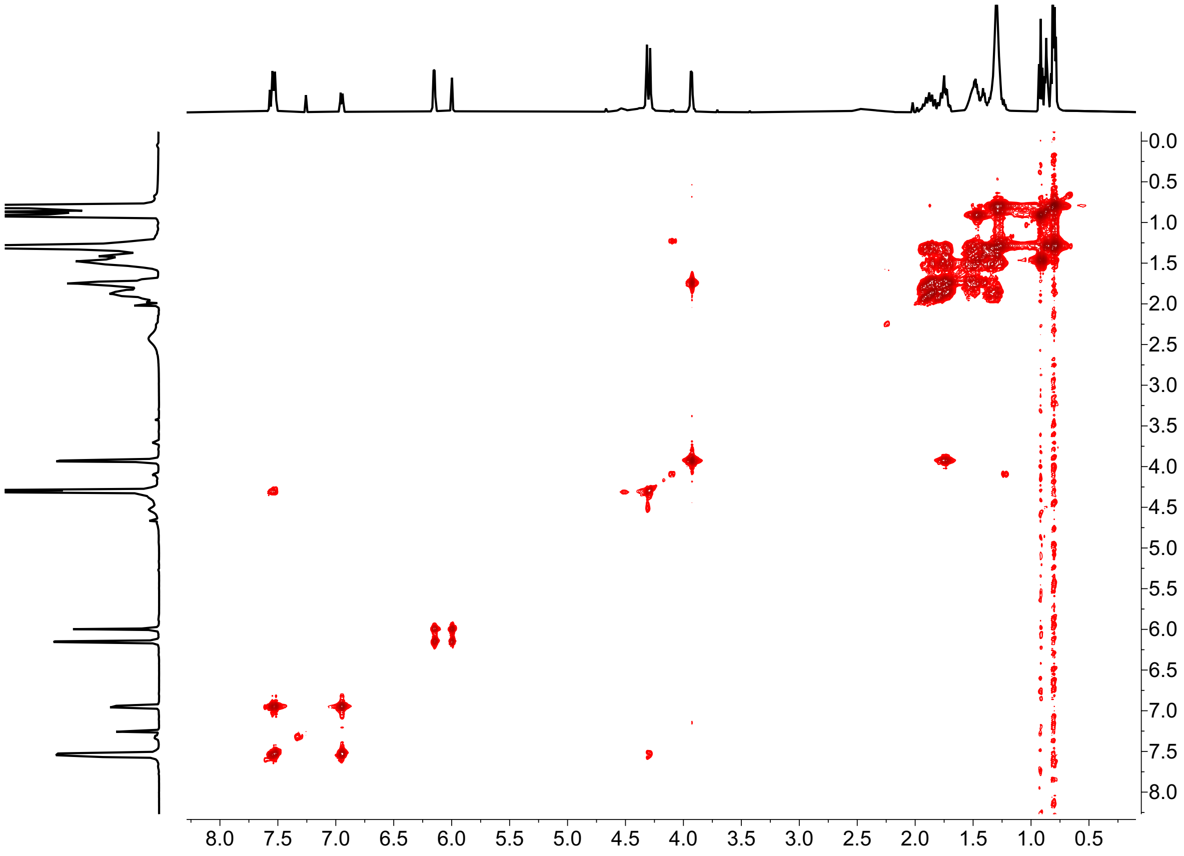

COSY-NMR spectrum

11. NMR spectroscopy studies

All binding constants were measured by ^{19}F and ^{1}H NMR titrations in a Bruker 500 MHz AVIII HD Smart Probe spectrometers. The host (H, phenol derivatives **D**, **DD**, **DDD**) was dissolved in toluene-*d8* or chloroform-*d3* at a known concentration. The guest (G, phosphine oxide derivatives **A**, **AA**, **AAA**) was dissolved in the host solution and made to a known concentration. A known volume of host was added to an NMR tube and the spectrum was recorded. Known volumes of guest in host solution were added to the NMR tube, and the spectra were recorded after each addition. The chemical shifts of the host spectra were monitored as a function of guest concentration and analysed using a purpose written software in Microsoft Excel. Errors were calculated as two times the standard deviation from the average value (95% confidence limit).

^{19}F and ^{1}H (donor CF_3 and O*H*) NMR chemical shifts and limiting complexation-induced changes in chemical shifts of the free host (ppm) obtained by fitting titration data measured in toluene-*d8* and chloroform-*d3* at 298 K to a 1:1 binding isotherm.

		^{19}F-NMR			^{1}H-NMR		
**Solvent**	**Complex**	δ_{free}	δ_{bound}	$\Delta\delta$	δ_{free}	δ_{bound}	$\Delta\delta$
**$CDCl_3$**	***D•A***	-61,1	-62,8	-1,7	5,7	11,3	5,6
	DD•AA	-61,0	-62,3	-1,2	5,5	10,3	4,8
	DDD•AAA	-61,0	-62,2	-1,2			
		-61,1	-62,2	-1,0			
**Toluene**	***D•A***	-61,8	-62,3	-0,5	4,9	11,6	6,6
	DD•AA	-61,5	-61,8	-0,3			
	DD•AA^{IMINE}	-61,5	-61,9	-0,4			

11.1 Titration D vs A in chloroform (^{1}H and ^{19}F-NMR).

Titration of **A** (40.59 mM) into **D** (2.27 mM) in $CDCl_3$ (^1H and ^{19}F-NMR).

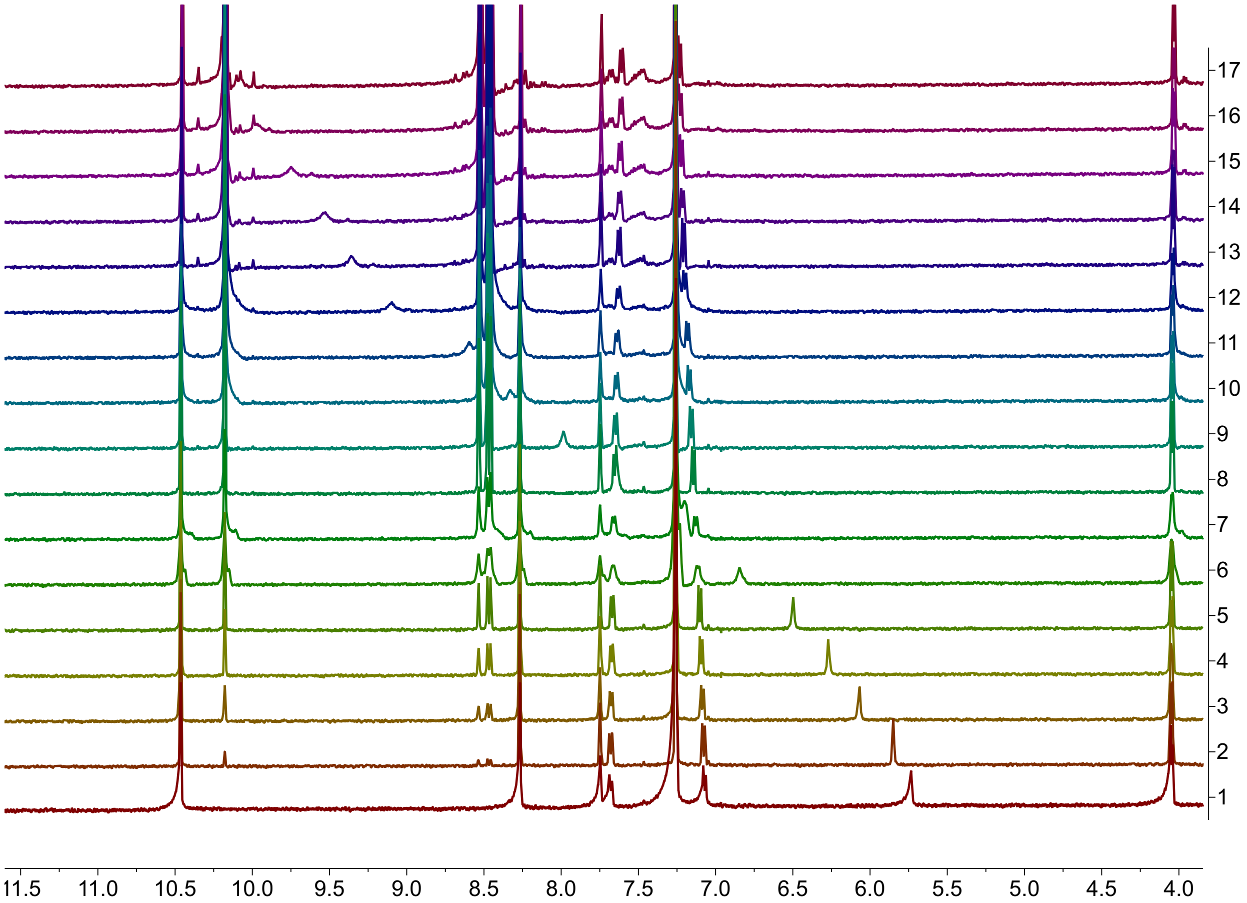

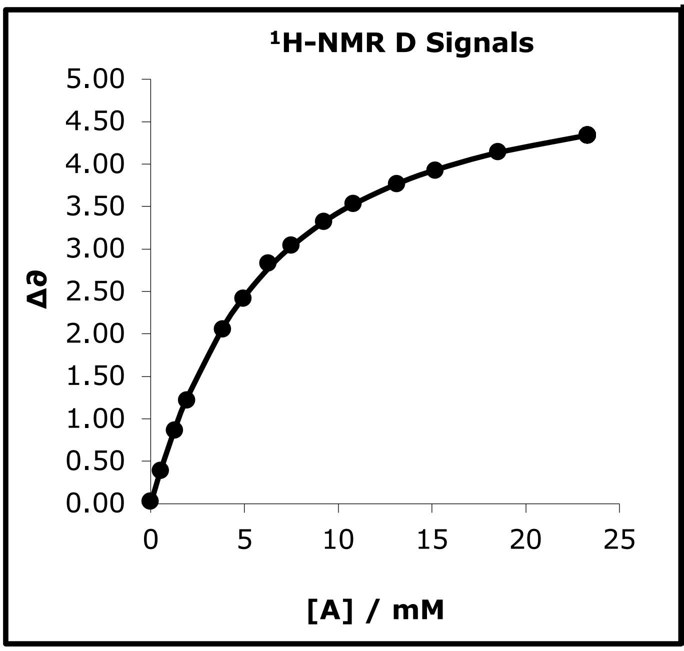

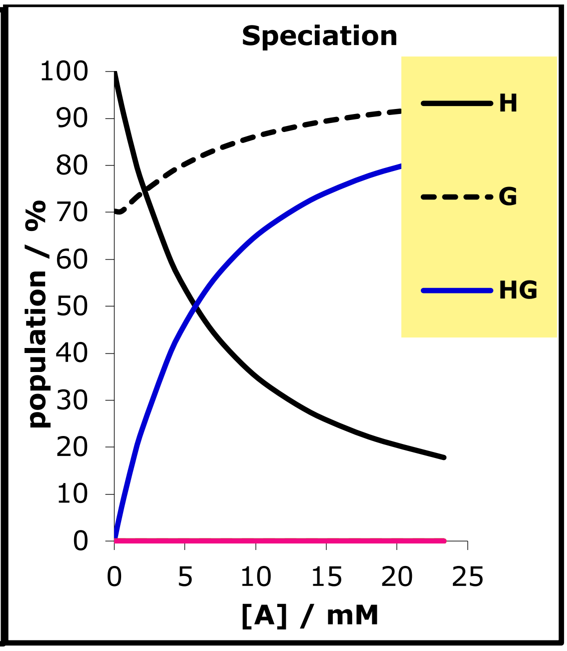

Plot of the change in chemical shift of the **D** ^1H signal as a function of [**A**] (the line represents the best fit to a 1:1 binding isotherm) and speciation as a function of [**A**].

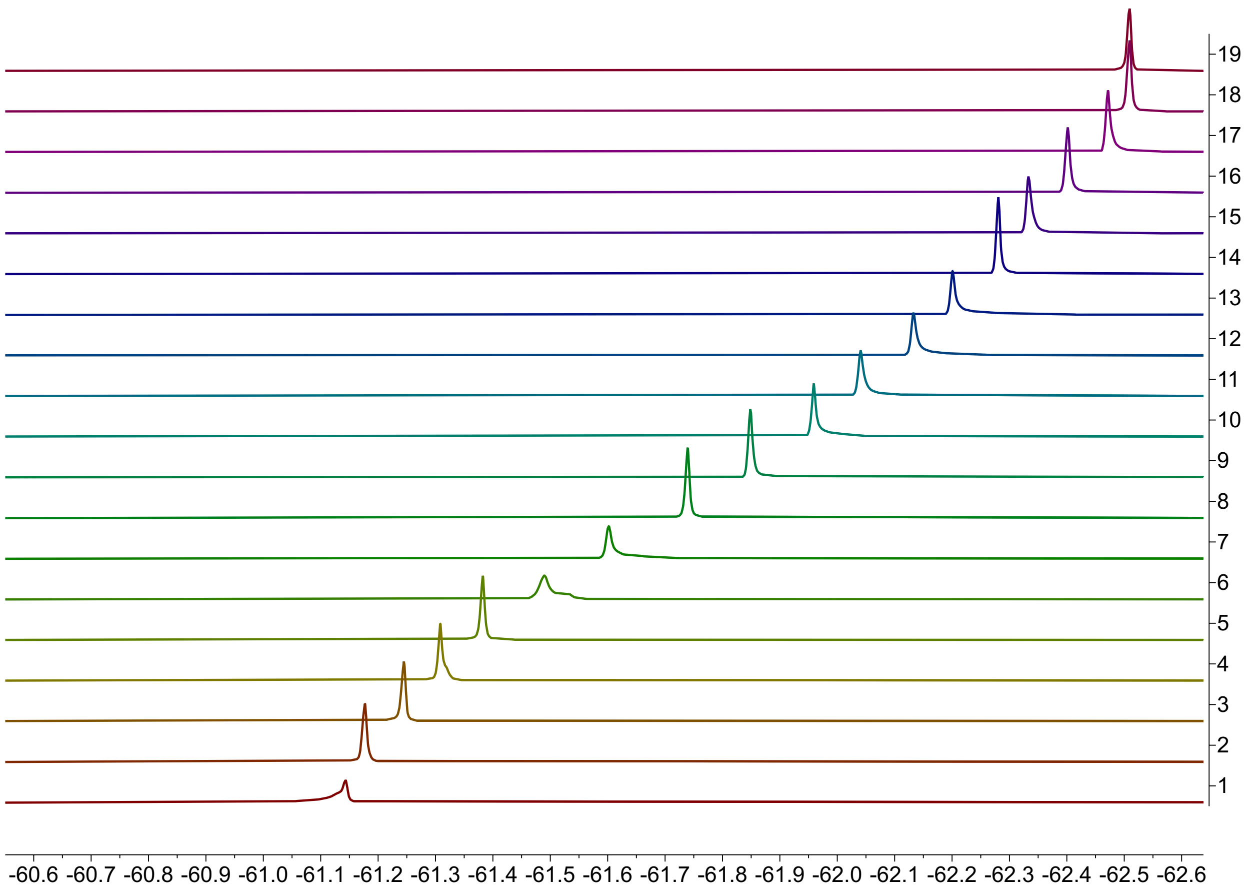

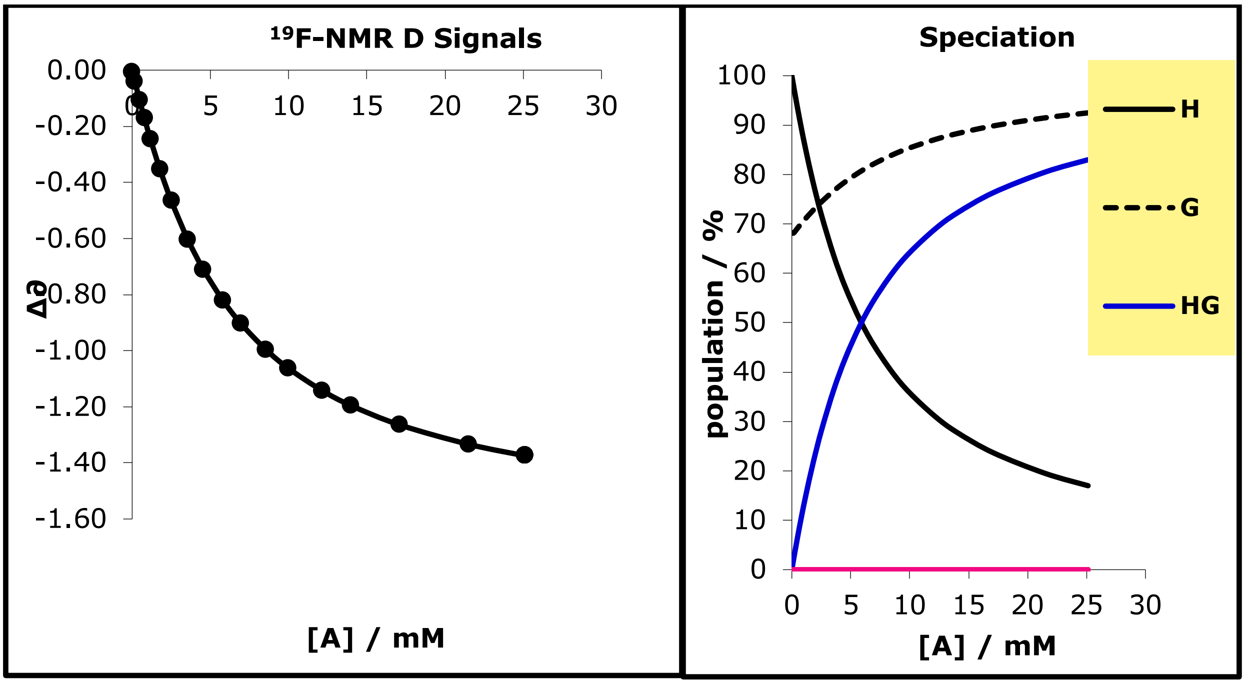

Plot of the change in chemical shift of the **D** ^{19}F signal as a function of [**A**] (the line represents the best fit to a 1:1 binding isotherm) and speciation as a function of [**A**].

11.2 Titration DD vs AA in chloroform (^{1}H and ^{19}F-NMR).

Titration of **AA** (10.08 mM) into **DD** (2.28 mM) in $CDCl_3$ (^{1}H and ^{19}F-NMR)

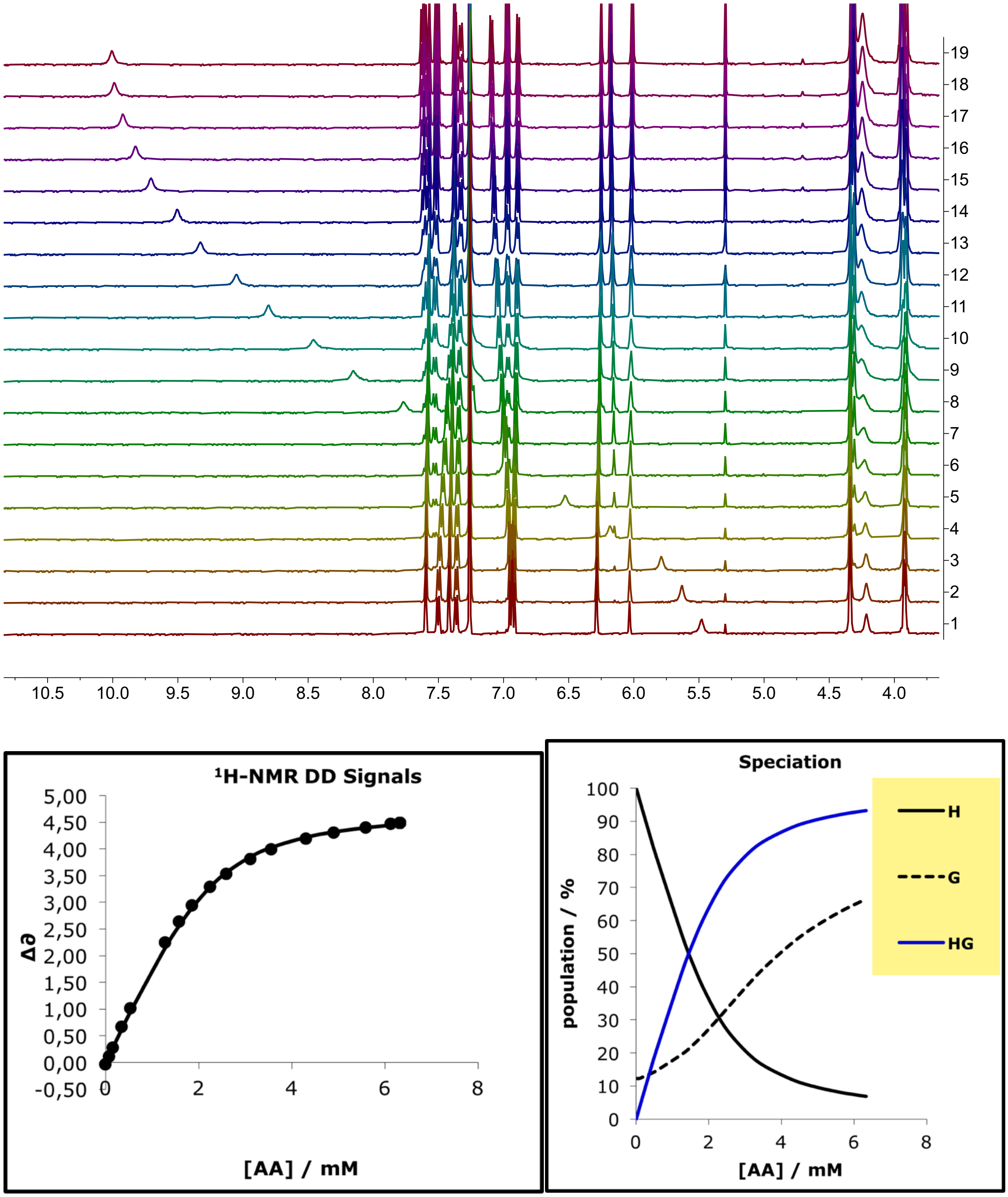

Plot of the change in chemical shift of the **DD** ^{1}H signal as a function of [**AA**] (the line represents the best fit to a 1:1 binding isotherm) and speciation as a function of [**AA**].

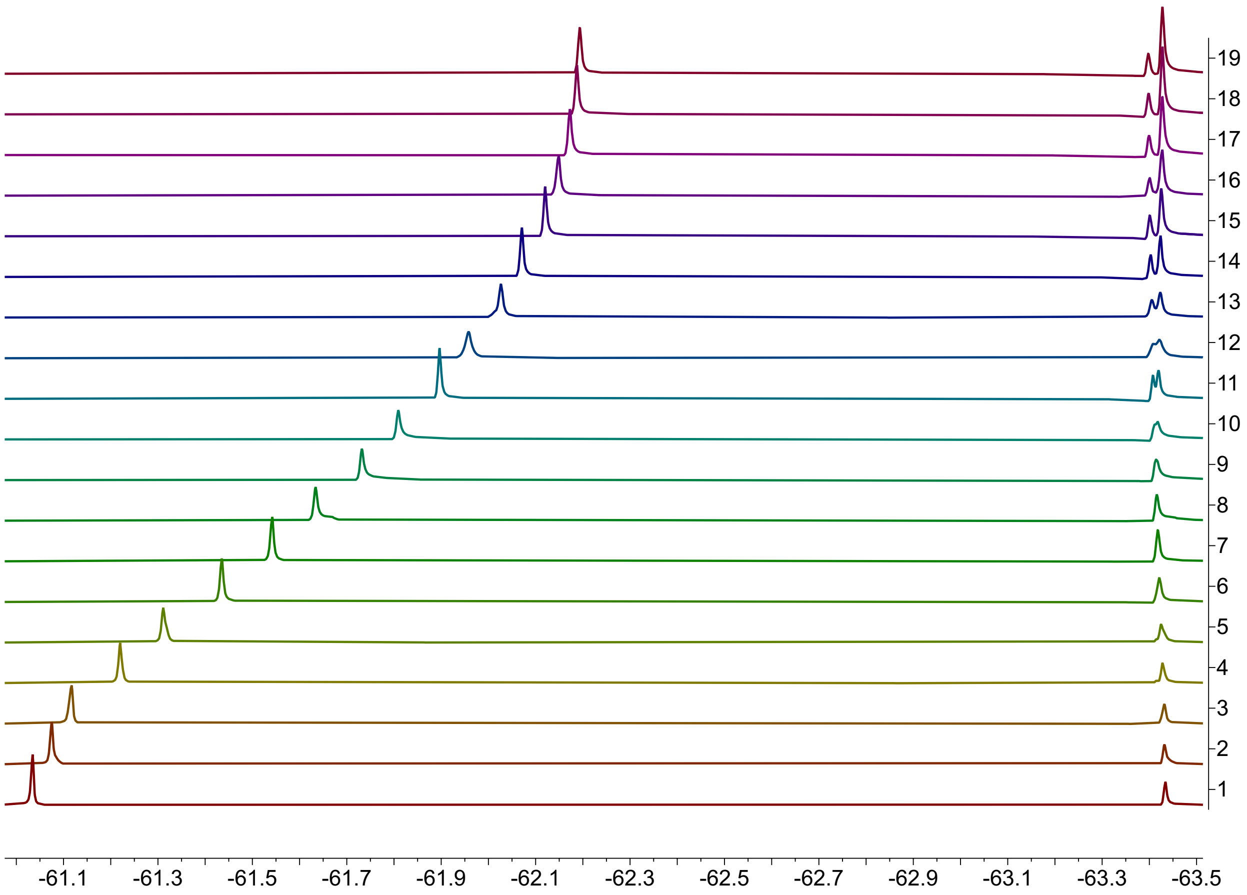

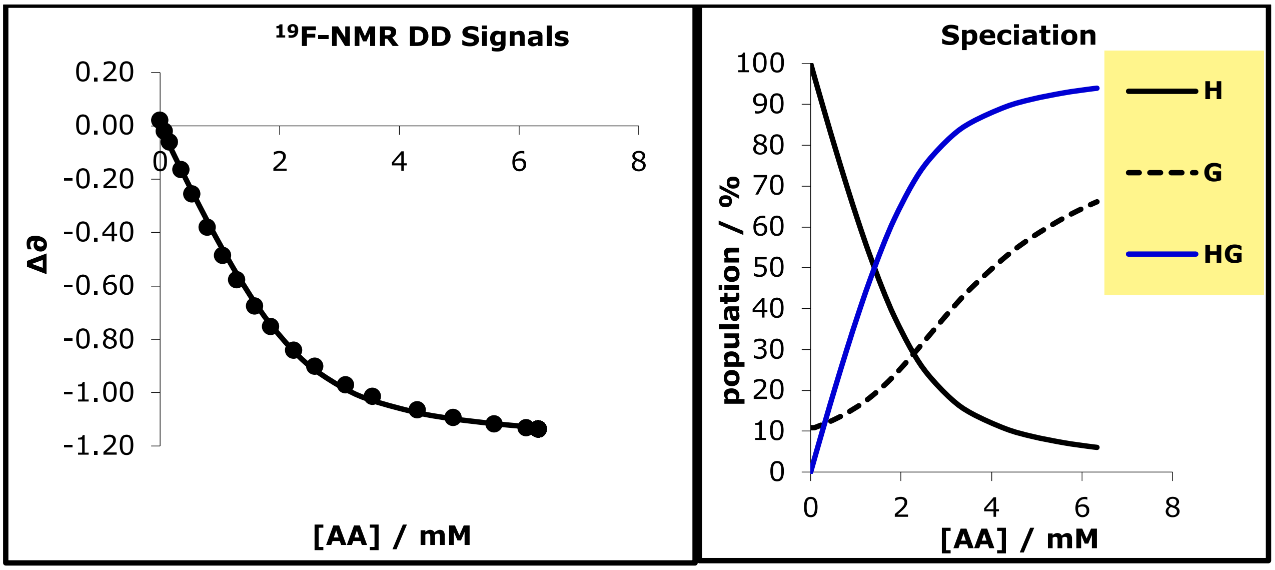

Plot of the change in chemical shift of the **DD** ^{19}F signal as a function of [**AA**] (the line represents the best fit to a 1:1 binding isotherm) and speciation as a function of [**AA**].

11.3 Titration DDD vs AAA in chloroform (^{19}F-NMR).

Titration of **AAA** (1 mM) into **DDD** (0.1 mM) in $CDCl_3$ (^{19}F-NMR)

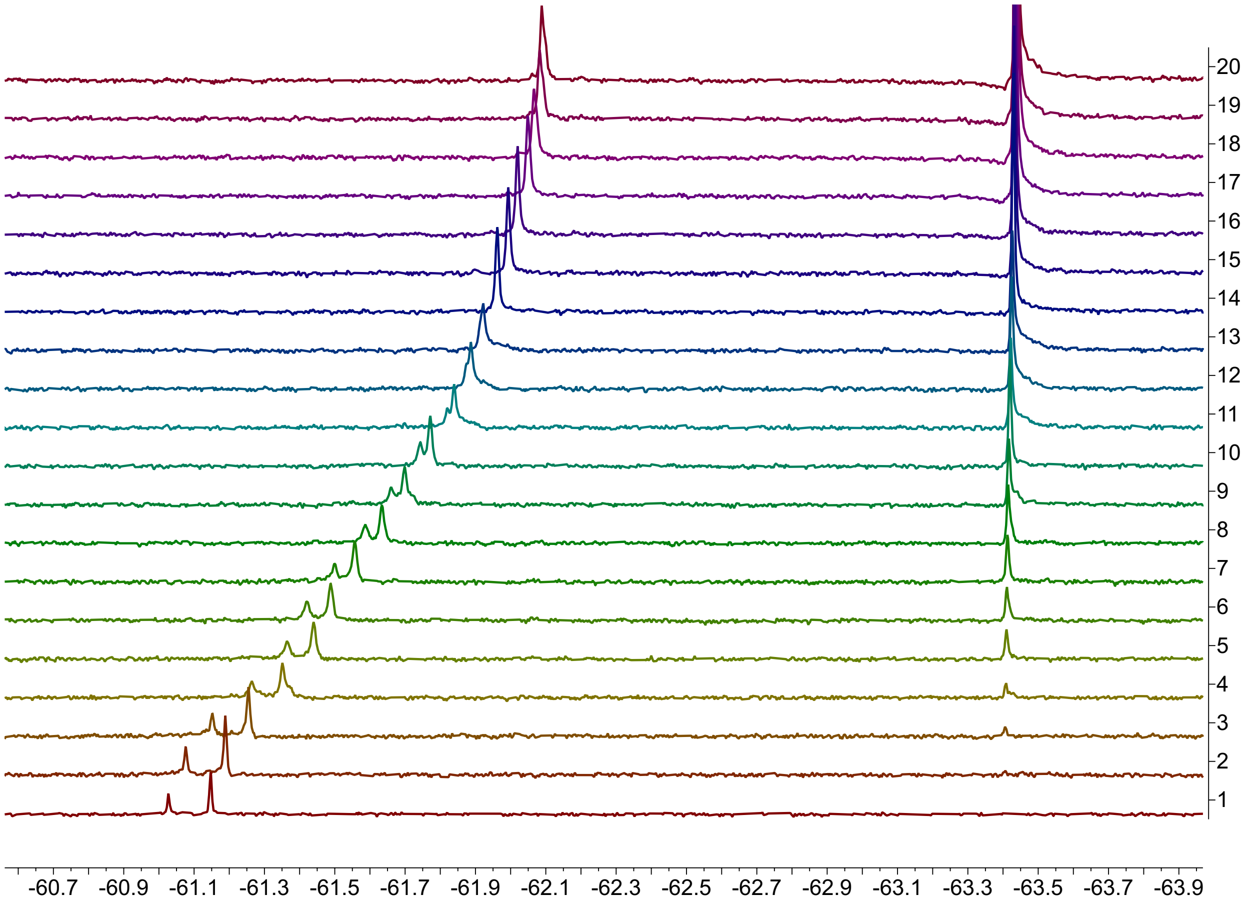

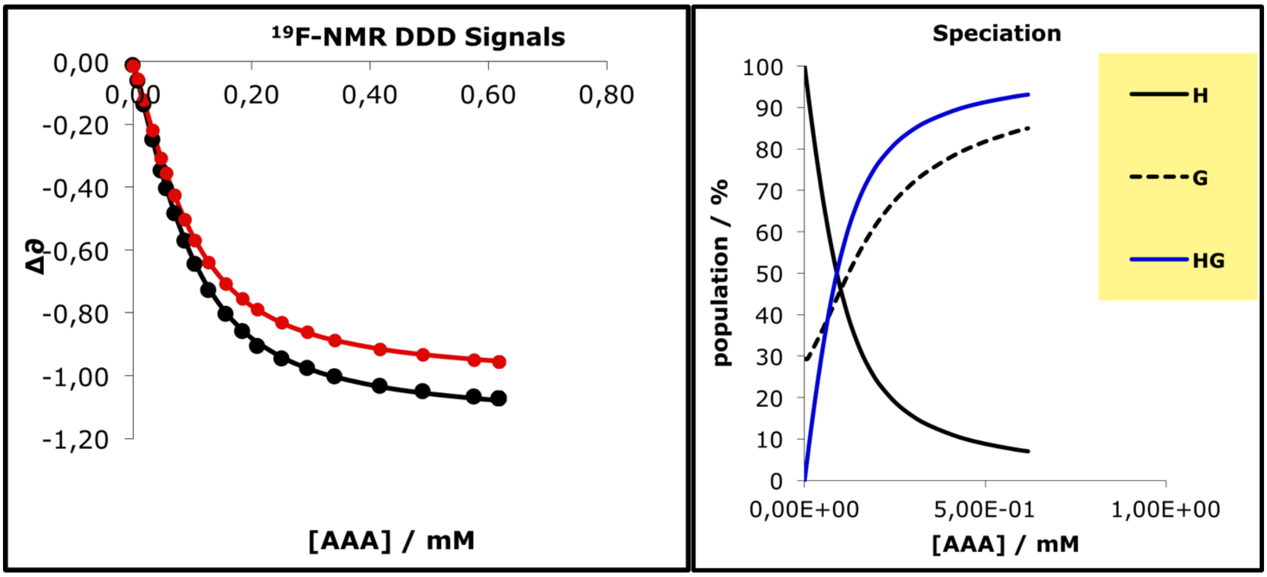

Plot of the change in chemical shift of the **DDD** ^{19}F signals as a function of [**AAA**]: red for the internal **D** monomer, black for the two external **D** monomers (the lines represent the best fit to a 1:1 binding isotherm) and speciation as a function of [**AAA**].

11.4 Titration D vs A in toluene (^{1}H and ^{19}F-NMR).

Titration of **A** (6.70 mM) into **D** (2.31 mM) in Toluene (^{1}H and ^{19}F-NMR).

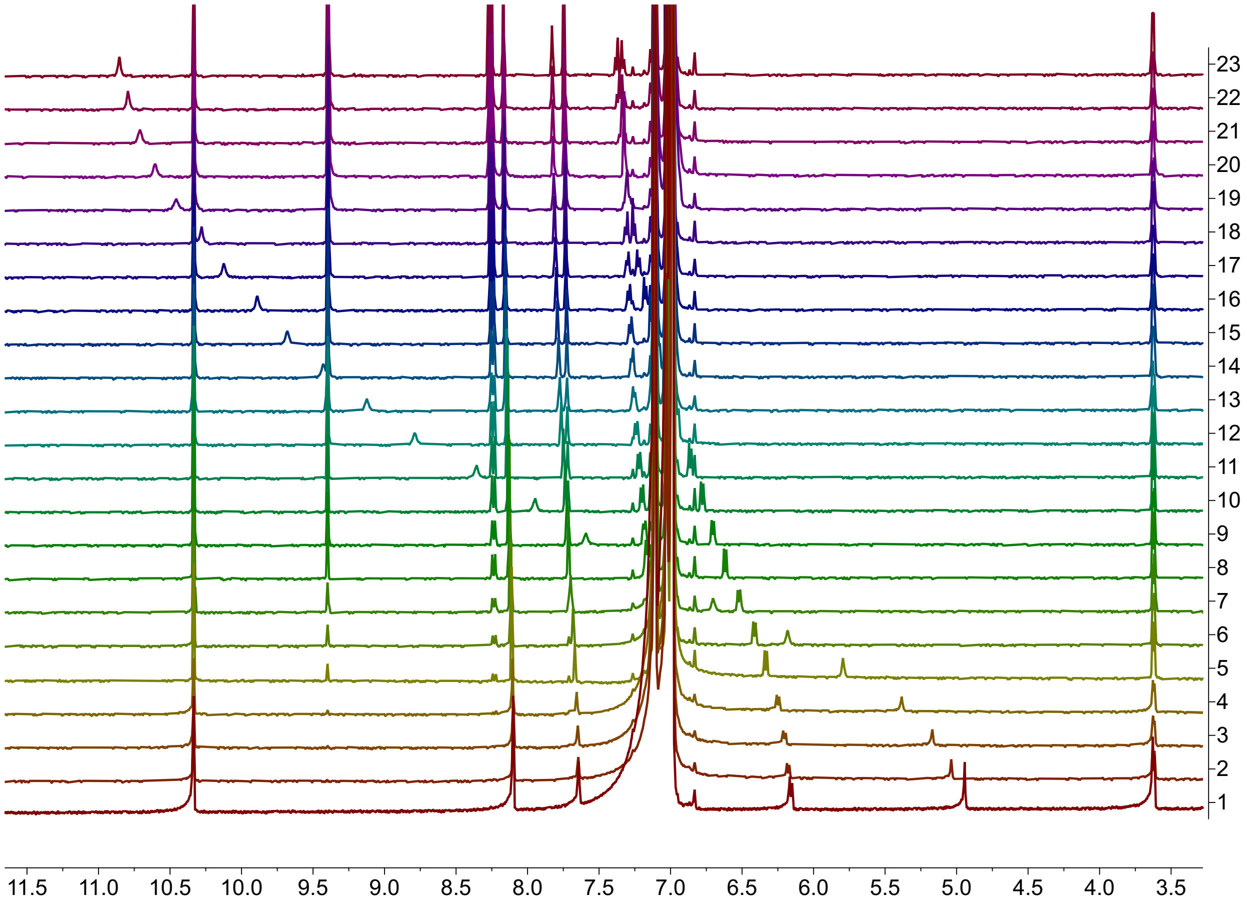

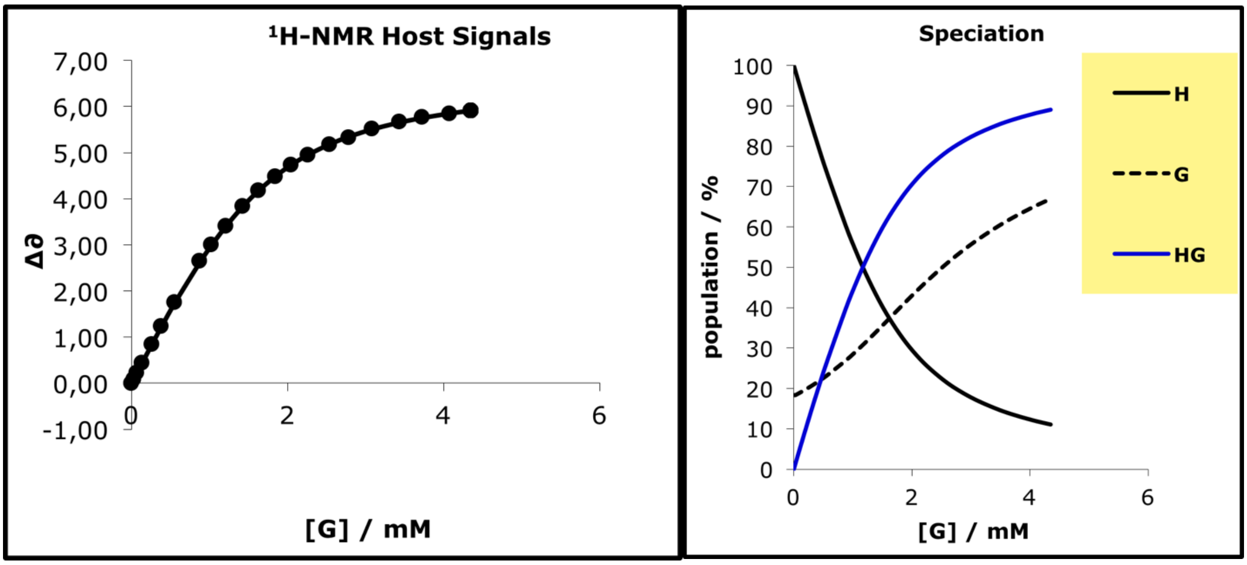

Plot of the change in chemical shift of the **D** ^{1}H signal as a function of [**A**] in toluene (the line represents the best fit to a 1:1 binding isotherm) and speciation as a function of [**A**].

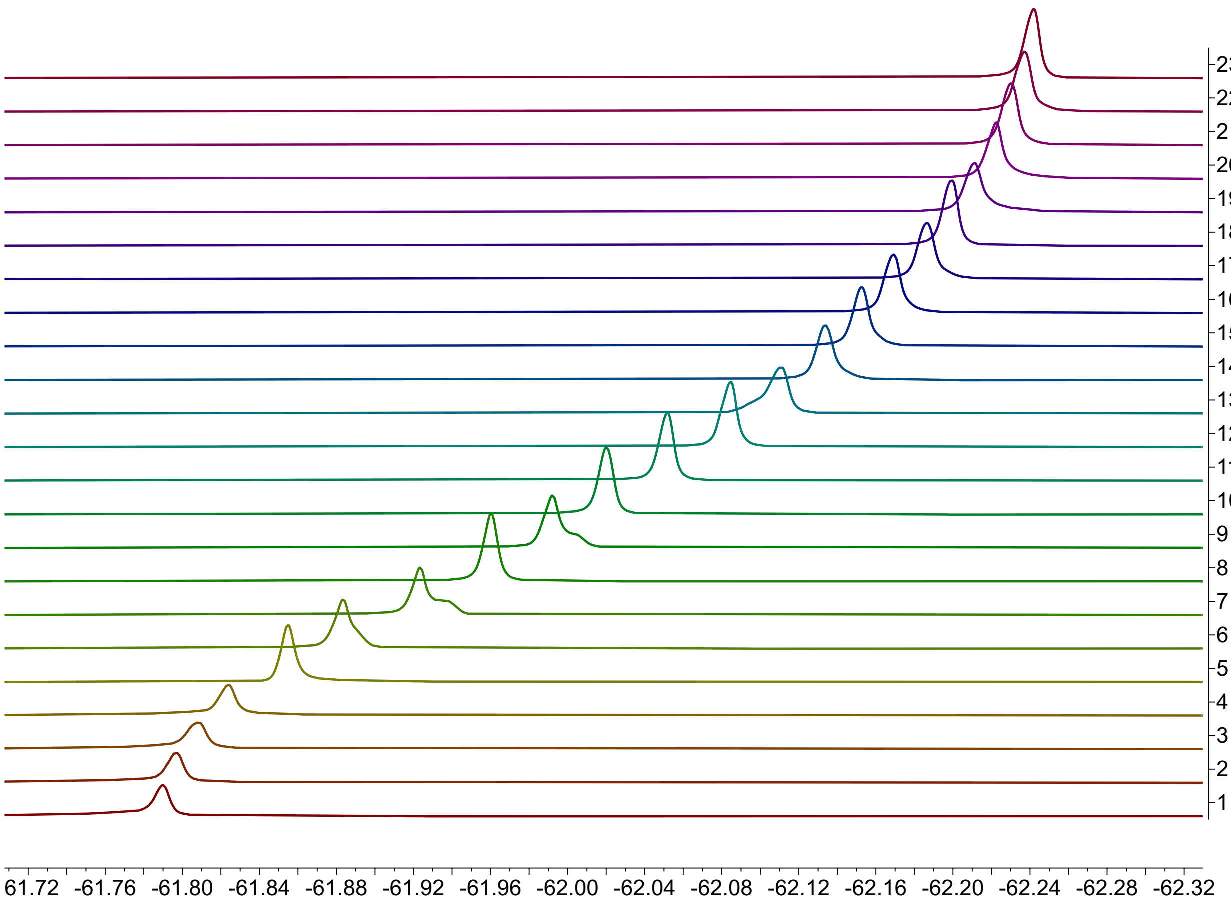

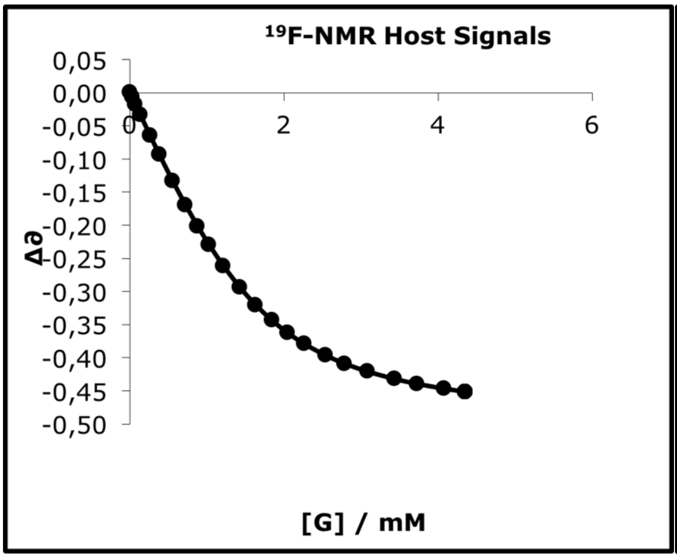

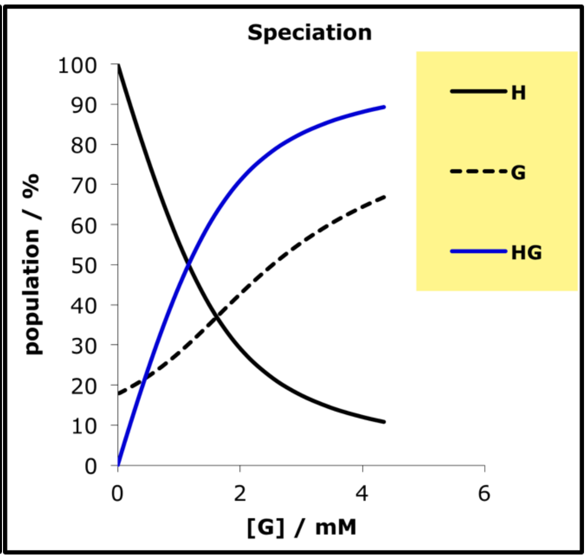

Plot of the change in chemical shift of the **D** ^{19}F signal as a function of [**A**] in toluene (the line represents the best fit to a 1:1 binding isotherm) and speciation as a function of [**A**].

11.5 Titration DD vs AA in toluene (^{1}H and ^{19}F-NMR).

Titration of **AA** (1.00 mM) into **DD** (0.10 mM) in Toluene (^{19}F-NMR).

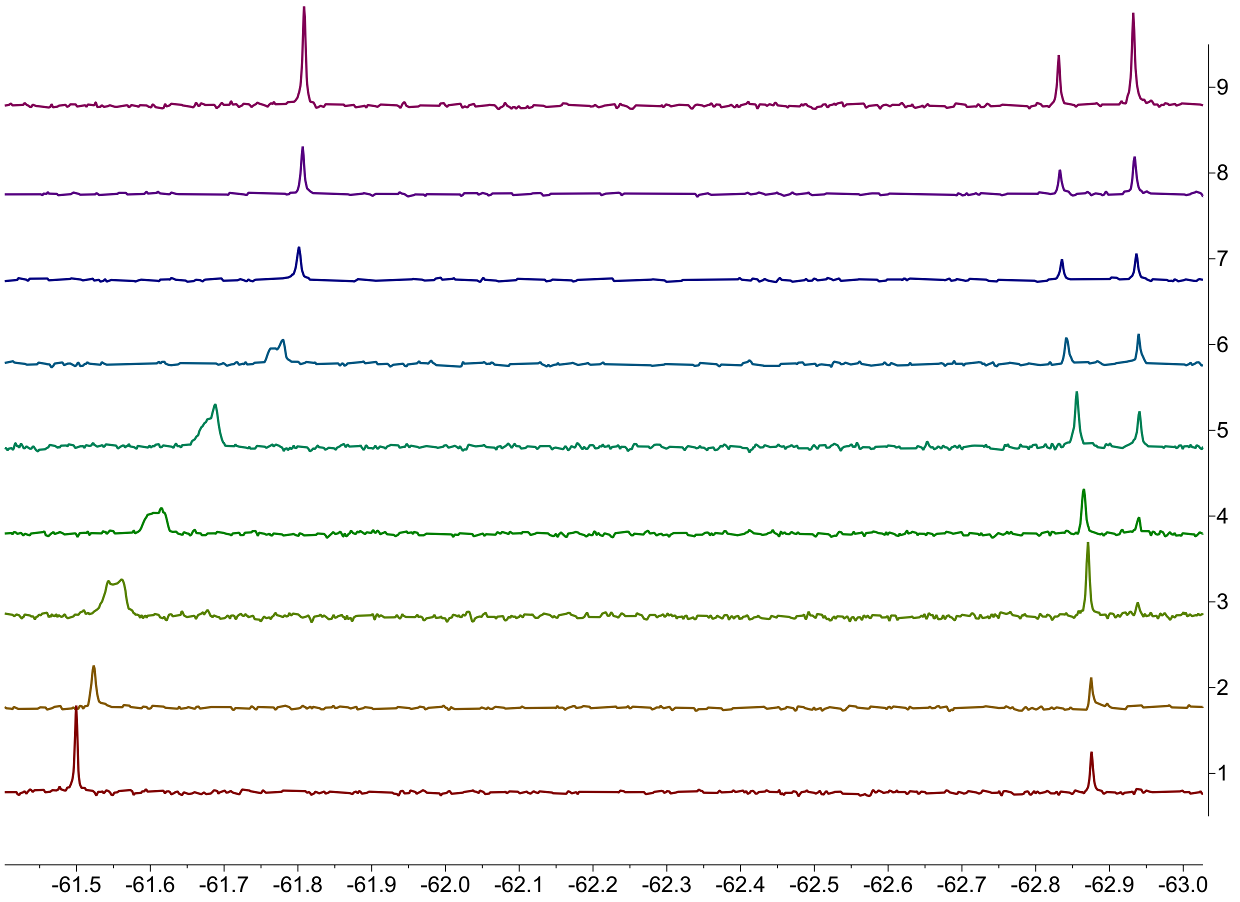

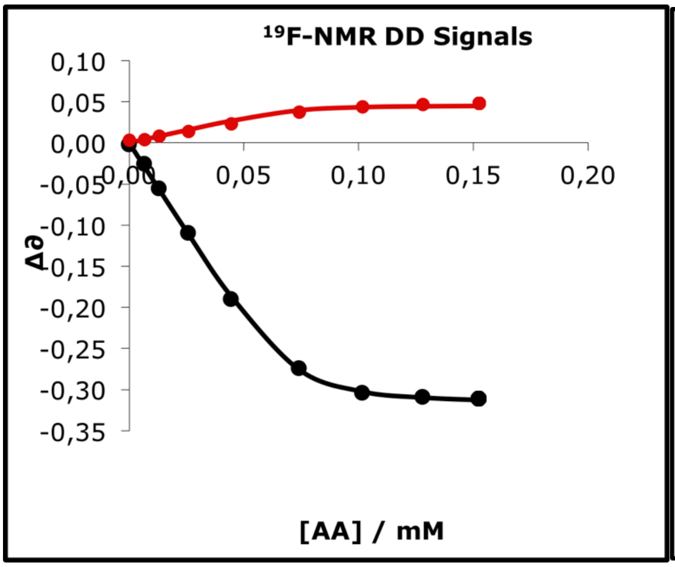

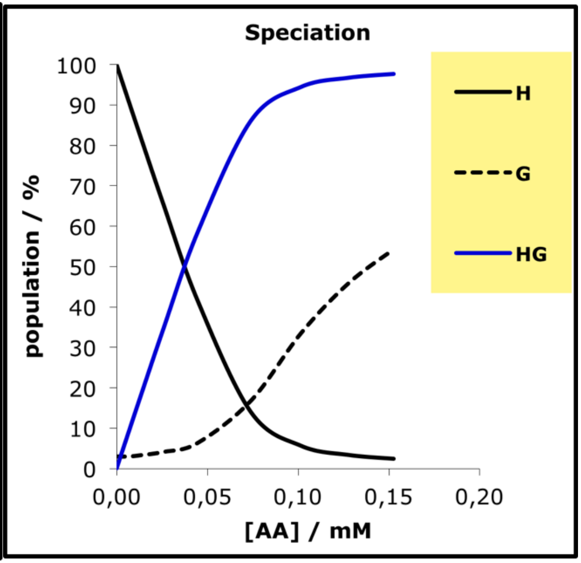

Plot of the change in chemical shift of the **DD** ^{19}F signals as a function of [**AA**]: red for the aniline backbone CF_3, black for the **D** monomers CF_3 (the lines represent the best fit to a 1:1 binding isotherm) and speciation as a function of [**AA**].

11.6 Titration DD vs dimer imine AA^{IMINE} in toluene (^{1}H and ^{19}F-NMR).

Titration of **AA**^{IMINE} (1.00 mM) into **DD** (0.099 mM) in Toluene (^{19}F-NMR).

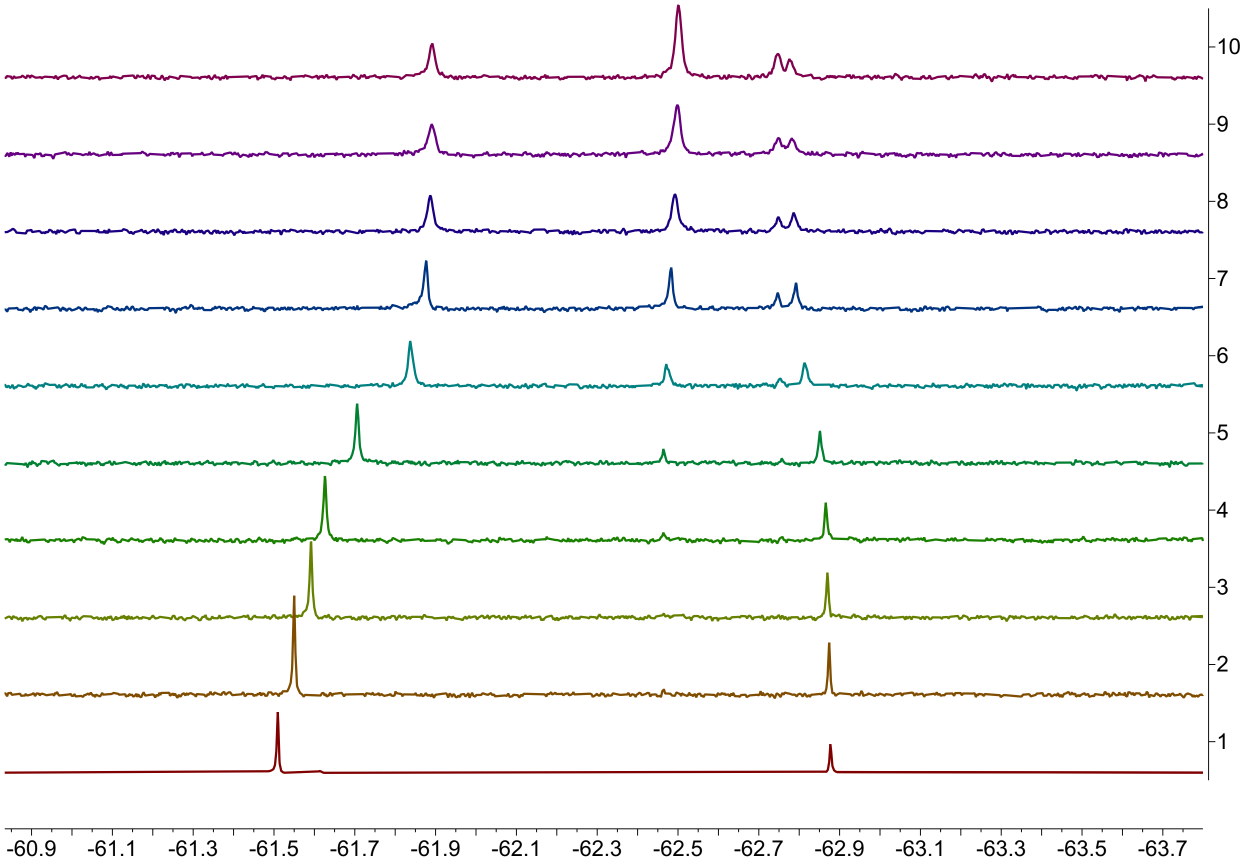

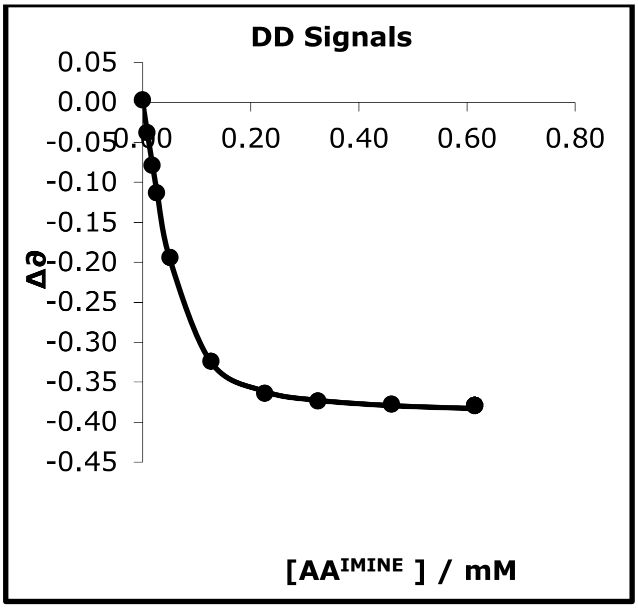

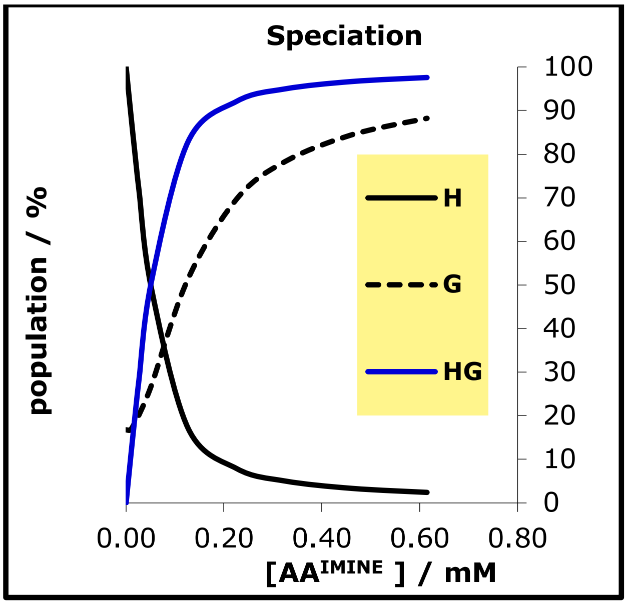

Plot of the change in chemical shift of the **DD** ^{19}F signals as a function of [**AA**^{IMINE}]: black for the **D** monomers CF_3 (the lines represent the best fit to a 1:1 binding isotherm – K = 7,43 E+04) and speciation as a function of [**AA**^{IMINE}].

12.References

1 D. Reinhard, L. Schöttner, V. Brosius, F. Rominger, M. Mastalerz, *Eur. J. Org. Chem.* **2015**, 3274.
2 L. Gonzalez-Bulnes, I. Ibanez, L. M. Bedoya, M. Beltran, S. Catalμn, J. Alcamí, S. Fustero, J. Gallego *Angew. Chem. Int. Ed.*, **2013**, *52*, 13405.
3 D. Reinhard, L. Schöttner, V. Brosius, F. Rominger, M. Mastalerz, *Eur. J. Org. Chem.* **2015**, 3274.
4 D. Nűñez-Villanueva and C. A. Hunter, Chem. Sci., 2017, 8, 206.
